# Body surface potential mapping of the cortico-muscular axis using smart textile electrode arrays

**DOI:** 10.1038/s41467-026-75134-1

**Published:** 2026-07-08

**Authors:** Ruben Ruiz-Mateos Serrano, Charlie Brunt, Xudong Tao, Maciej Zajaczkowski, Antonio Dominguez-Alfaro, Matias L. Picchio, Daniele Mantione, Emmanuel M. Drakakis, David Mecerreyes, George G. Malliaras

**Affiliations:** 1 Institute for Biomedical Innovation, University of Cambridge, Cambridge, UK; 2 Department of Bioengineering, Faculty of Engineering, Imperial College London, London, UK; 3 POLYMAT, University of the Basque Country UPV/EHU, Av.Tolosa 72, 20018 Donostia-San Sebastian, Gipuzkoa Spain; 4 IKERBASQUE, Basque Foundation for Science, Bilbao, Spain; 5 POLYMAT, Department of Mining-Metallurgy Engineering and Materials Science, School of Engineering, University of the Basque Country (UPV/EHU), Bilbao, Spain; 6 Electrical Engineering Division, Department of Engineering, University of Cambridge, Cambridge, UK; 7 Present Address: Instituto de Microelectrónica, IMSE-CNM, (CSIC Universidad de Sevilla), Av. Américo Vespucio 28, 41092 Sevilla, Spain

**Keywords:** Biomedical engineering, Electrophysiology, Preclinical research, Sensorimotor processing

## Abstract

Cutaneous electrophysiology is a fundamental non-invasive technique for assessing electrically active organs such as the brain, heart, and muscles. Standard approaches, however, are limited in spatial resolution, reducing sensitivity to certain pathological features. The development of body surface potential mapping using electrode arrays has helped overcome these limitations, enhancing the diagnostic power of cutaneous recordings, yet clinical adoption remains constrained by challenges in electrode performance, wiring complexity, wearability, data transmission, and interpretability. Here, we present a hybrid e-textile electrode array system that overcomes these barriers, enabling simultaneous mapping of electrical activity along the cortico-muscular axis. The system combines application-specific conducting polymer coatings to improve electrode performance, a flexible fabrication process for robust connectivity and wearability, and interpretable machine learning algorithms for data analysis. In controlled single-subject experiments, we demonstrate reliable muscle and brain recordings, enabling classification of grasped object shapes and somatosensory stimuli. Simultaneous multi-site recordings along the cortico-muscular axis provide spatial maps of reaction time distributions and allow prediction of muscle activation patterns from cortical activity. This platform establishes a framework for wearable, multi-modal electrophysiological mapping and non-invasive study of cortico-muscular dynamics, representing a step towards practical brain–body interfaces with applications in neurorehabilitation, prosthetics, and human–machine interaction.

## Abstract (web summary)

Cutaneous electrophysiology is limited by spatial resolution and wearability constraints. Here, the authors develop a hybrid e-textile electrode array enabling simultaneous cortico-muscular mapping, improving signal quality and decoding of sensory and motor activity for wearable brain-body interfaces

## Introduction

Voluntary movement and sensorimotor integration depend on dynamic communication between the brain and skeletal muscles. This bidirectional relationship, often referred to as the cortico-muscular axis, is central to motor control and is increasingly recognised as a key target for both basic neuroscience and clinical intervention^1–3^. A widely used technique for investigating this axis is cortico-muscular coherence (CMC), which measures frequency-domain coupling between cortical signals, typically recorded via electroencephalography (EEG) or magnetoencephalography (MEG), and peripheral muscle activity recorded via electromyography (EMG)^4–6^. CMC has advanced our understanding of motor system function in both healthy individuals and patients with neurological disorders^7–13^.

However, CMC presents important limitations. It relies on narrow-band temporal correlations and generally involves only a small number of electrodes, limiting spatial resolution and the ability to capture distributed activity across the body^14^. Furthermore, the amplitude of CMC is highly variable across individuals and experimental conditions, and its physiological interpretation remains uncertain^15–18^. These constraints have limited its use in characterising complex, spatially distributed brain-muscle interactions during naturalistic motor behaviour.

Body surface potential mapping (BSPM) provides a complementary approach that can overcome many of these limitations. By recording dense arrays of voltage signals from the skin surface, BSPM has been successfully applied in cardiology, neurology and neuromuscular medicine to detect phenomena such as ischemic propagation, epileptic foci and muscle fasciculations^19–25^. Despite these advances, BSPM has not been widely used to study the cortico-muscular axis, in part due to challenges related to electrode array scalability, wearability and simultaneous multi-site signal stability^6,26–36^.

Recent advances in smart flexible and textile wearable technologies have produced a wide variety of wearable electrophysiological systems, differing substantially in electrode materials, fabrication strategies, supported modalities, and levels of integration. However, the field remains highly fragmented: most reported systems are optimised for a single modality or anatomical site, often lack spatially dense electrode arrays, and rarely support simultaneous multimodal recordings across different body regions. To clarify this landscape, Supplementary Table S1 provides an extensive comparative review of representative textile-based and flexible wearable electrophysiology systems, spanning early knitted and embroidered electrodes to more recent flexible and hybrid approaches. The table systematically contrasts modality coverage, electrode materials, fabrication methods, support for multimodal and simultaneous recordings, array-based sensing, and the presence of multilayer or via-enabled interconnect architectures. This comparison highlights that, while multimodal sensing has been demonstrated in isolated cases, unified architectures capable of supporting spatially resolved, simultaneous brain-muscle recordings with integrated multilayer interconnects remain uncommon.

In this Article, we describe a hybrid e-textile BSPM system that enables simultaneous, multimodal mapping of electrophysiological activity across the brain and peripheral muscles during voluntary movement and sensory tasks. The platform integrates low-impedance, application-specific conducting polymer coatings, textile-based electrode arrays and wireless data acquisition to perform recordings during naturalistic movement and real-world motor tasks. We use this system to identify spatiotemporal patterns of activation and directional signal flow between the cortex and forearm muscles. This approach offers enhanced spatial sampling and signal detail compared to conventional coherence-based methods, enabling more precise characterisation of cortico-muscular interactions during dynamic behaviour. We further demonstrate the prediction of muscle activation patterns from cortical surface potentials, highlighting structured relationships between brain activity and peripheral motor output. These results show the potential of BSPM as a tool for studying distributed neuromuscular function and for advancing our understanding of sensorimotor integration in both research and clinical contexts.

## Results and Discussion

### Structural design of smart textile electrode arrays

**Fig. 1:**
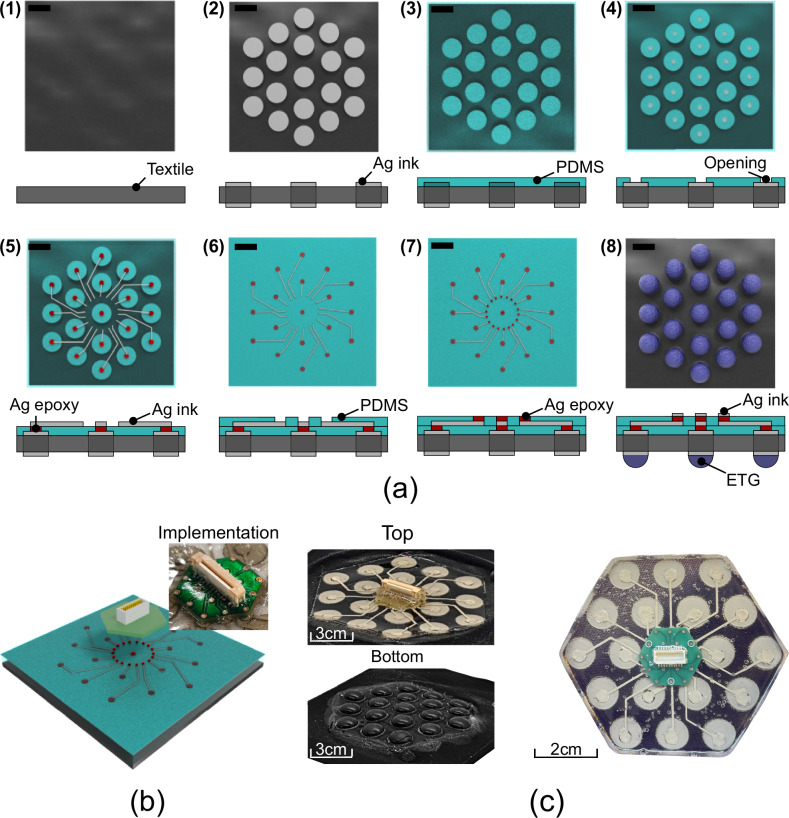
Fabrication process of the smart textile electrode array. **a** Sectional diagram of a hexagonal device illustrating the key fabrication steps, accompanied by top view rendered images corresponding to each stage (layers not at scale). Scale bar: 1 cm. (1) Textile substrate prepared as the base layer, (2) silver electrodes deposited within the textile, (3) electrodes embedded in PDMS for insulation and mechanical stability, (4) PDMS layer laser-rastered to selectively expose electrode contact sites, (5) silver epoxy vias and silver ink tracks deposited to form conductive pathways, (6) PDMS overlayer brushed over the tracks for sealing, (7) second silver epoxy deposition applied to form interface pads for PCB connection, (8) blade-coating of uncured silver ink and PEDOT:LS/Gly:ChCl/gelatin ETG deposited on the opposite side to form the skin-contacting electrodes and complete the multilayer array. **b** 3D rendering of the proposed integration with custom PCB used for interfacing with external electronics, along with real implementation. **c** Final implementation of a hexagonal multilayer e-textile device, showing top and bottom views of the fabricated array.

To address hardware limitations in electrode array implementation for BSPM, we developed a hybrid e-textile fabrication approach (Fig. 1a). This method leverages the porous structure of woven textiles, allowing conductive paste to permeate through the fabric and electrically connect both sides without piercing the substrate^37^. Unlike stitched, embroidered or surface-printed textile electrodes, this process forms intrinsic vertical electrical interconnects through the fabric without mechanically damaging fibres or imposing rigid via structures. Blade-coating conductive paste into the porous textile enables the material to infiltrate and mechanically interlock with the fibre network, while subsequent elastomeric lamination anchors the conductive pathways within the textile matrix. This combined ink-infiltration and elastomeric lamination strategy suppresses delamination, minimises crack formation and reduces electrical degradation under repeated bending and handling. Electrodes are formed on one side of the fabric to interface with the skin, while conductive tracks on the opposite side collect signals. This architecture decouples electrode placement from lateral routing constraints, enabling compact, spatially dense electrode layouts that would be difficult to achieve using conventional textile interconnect strategies. Here, “spatially dense” refers to electrode layouts that exceed what is practically achievable with traditional stitched or embroidered interconnects for a given textile area, rather than to the densities typical of silicon-based microelectrode arrays.

The fabrication workflow proceeds as follows (see Fig. 1a). The fabric is first pre-stretched on a frame (step 1), and two aligned laser-patterned tape masks are applied to define the electrode layout. Silver nanoparticle paste is then blade-coated over both masks, soaking through the textile to form vertical electrical connections (step 2) (Suppl. Figs. S1–S2). Following this preparation, a multi-layer structure is built stepwise to create a robust and electrically isolated device. Inspired by embedded dyeing strategies in textiles^38^, polydimethylsiloxane (PDMS) soaking is used to laminate and insulate the routing side of the fabric (step 3), followed by laser rastering of the PDMS to selectively expose electrode contact sites (step 4). Conductive pathways are formed by depositing silver epoxy vias and blade-coated silver ink tracks (step 5), which are then sealed with a PDMS overlayer to enhance adhesion and mechanical stability (step 6). Interface pads for external PCB connection are established through a second silver epoxy deposition (step 7).

External signal routing is achieved using custom-designed interface printed circuit boards (PCBs) placed above the insulated routing layer (Fig. 1b and Suppl. Fig. S3). The underside of each PCB includes via holes aligned with the contact pads in the textile array, while the top side houses connectors or recording circuitry. Electromechanical coupling is achieved by placing uncured silver paste over the silver epoxy contact pads (step 8), after which the PCB is aligned and brought into contact. The assembly is then sealed with an additional layer of PDMS to improve mechanical stability and environmental isolation, resulting in a final device thickness of 2 mm ± 0.5 mm. This approach enables multi-layer integration through alternating conductive and insulating layers while maintaining compatibility with standard electrophysiology hardware. The complete fabrication workflow is schematically summarised in Fig. 1a, b and described in detail in the Methods section.

An alternative routing strategy based on flexible printed circuit boards (FPCBs) can be employed instead of blade-coated silver tracks to interface the textile electrodes with external electronics. In this configuration, the bottom-side pads of the FPCB align with the Ag epoxy vias above the textile electrodes, while the top side accommodates connectors or recording circuitry (Suppl. Fig. S4 and Methods). The FPCB approach simplifies fabrication and improves connection reliability by leveraging established PCB manufacturing techniques; however, it introduces a trade-off in mechanical behaviour, reducing flexibility and breathability relative to fully laminated silver-track architectures. Selection between FPCB-based routing and laminated conductive tracks can therefore be guided by the specific mechanical, ergonomic, and integration requirements of the intended application.

While the multilayer textile architecture enables scalable and spatially dense routing, the quality and stability of electrophysiological recordings are ultimately governed by the electrode-skin interface^39^. Conventional electrodes, such as Ag/AgCl or bare metallic, are effective in stationary laboratory settings but have several limitations for wearable, flexible, and high-density applications^40^. Their rigid, planar structure impairs conformal contact with curved or moving skin, increasing motion artifacts and reducing signal fidelity. Metals are purely electronic conductors, whereas biological tissues conduct primarily via ions; this mismatch limits transduction efficiency, particularly for gel-free electrodes. Furthermore, gels used with Ag/AgCl electrodes are non-ideal for long-term recordings as they dry out and compromise signal stability, and their size constraints make miniaturised, spatially dense arrays challenging to implement on textiles^41^. To overcome these limitations, we employ conducting polymers, which combine mechanical flexibility with mixed ionic-electronic conductivity, enabling soft, conformal, gel-free electrodes that maintain high signal quality even under repeated motion and over extended durations^42^.

Building on this principle, we developed electrodes composed of poly(3,4-ethylenedioxythiophene) doped with lignin sulfonate (PEDOT:LS), glycerol/choline chloride (Gly:ChCl), and gelatin^43^. PEDOT:LS provides mixed ionic-electronic conductivity, flexibility, biocompatibility, and enhanced volumetric charge transfer^44–47^. Gelatin improves mechanical stability and extends the coating beyond the electrode surface, enhancing skin-electrode contact, while Gly:ChCl, a deep eutectic solvent (DES), is a non-volatile, low-cost, biocompatible, room-temperature liquid salt that enhances ionic conductivity^48,49^. When combined, PEDOT:LS and Gly:ChCl form supramolecular eutectogels (ETGs)^43,50^, which, together with gelatin, yield electrodes with high ionic and electronic conductivity, mechanical flexibility, strong adhesion, and long-term stability exceeding 90 days without SNR degradation^47,51^. Gelatin-based PEDOT ETGs have demonstrated superior overall performance across a broad range of reported PEDOT-based ionic and eutectic material systems, including pure cholinium-based ionic materials^52,53^, PEDOT with ionic liquids and polysaccharides^54,55^, polycarbonates with ionic liquids^56^, and PEDOT blended with ionic liquids^57–59^, owing to their tissue-mimetic softness, conformal skin contact, and ability to reduce motion artifacts^35^. The non-volatile, gel-free nature of ETGs also enables scalable fabrication of high-density, multimodal electrode arrays via additive manufacturing and printing techniques^51,59^, providing a robust and environmentally friendly alternative to conventional metal or gel-based electrodes for long-term, high-quality electrophysiological recordings.

To deposit the conducting polymer coating, the fabric is inverted such that the exposed silver electrodes face upward. Pre-synthesised PEDOT:LS/Gly:ChCl/gelatin eutectogel (ETG) (synthesis described in Methods) is dispensed onto each electrode site in a fixed amount of 0.15 g per 10 mm diameter electrode. The deposited ETG is then heated in a convection oven, causing the material to melt and reflow. Owing to the hydrophobic nature and high viscosity of the ETG, the material adopts a stable, drop-like morphology that remains confined to the electrode footprint without lateral spreading. Upon cooling to room temperature, the ETG resolidifies to form a mechanically robust, gel-free electrode coating that protrudes above the textile surface by approximately 1 mm ± 5 µm, thereby enhancing conformal contact with the skin (see Methods for further details). The completed array, including top and bottom views of the laminated textile device with ETG-coated electrodes, is shown in Fig. 1c.

The hybrid multilayer architecture and ETG coating together confer robust mechanical and electrical performance in the fully assembled device. Mechanical compliance and conformability were quantified by measuring the minimum bending radius (MBR) at which electrical performance remained stable (Suppl. Figs. S5–S7), with four-point bending tests shown in Supplementary Fig. S8 (see Methods for details). Laminated silver-track devices exhibited an MBR of 0.5 mm, whereas the FPCB-based configuration showed an MBR of 1 mm, reflecting the increased stiffness introduced by rigid interconnect layers. These values lie within the 0.5–2 mm range commonly reported for conformable skin-interfacing wearables^60–62^. Despite the millimetre-scale multilayer structure, both configurations conform reliably to anatomical curvatures such as the forearm, chest and forehead without loss of electrical integrity. The system is intentionally non-stretchable to preserve fixed inter-electrode distances (IEDs), ensuring reproducible spatial sampling during use.

Mechanical robustness was evaluated through cyclic bending at curvature levels defined relative to the measured MBR. Devices underwent 100 bending cycles at radii slightly exceeding their respective MBR values (2 mm radius, κ = 500 m^−1^, for laminated silver-track devices; 3 mm radius, κ = 333 m^−1^, for FPCB-based devices), representing subcritical loading relevant to repeated physiological deformation. Electrical continuity testing across five devices per design confirmed all electrodes remained conductive, with no evidence of delamination or cracking (Suppl. Figs. S5–S7 and Methods). Electrical performance of the ETG electrodes has been extensively characterised in previous work; however, additional testing of five independent devices with ten repeated measurements confirmed expected impedance values at 50 Hz with a median of 0.427 MΩ and an interquartile range (IQR) of 0.529 MΩ across 19-electrode hexagonal arrays, with electrode-to-electrode variability of approximately 69.7 kΩ, providing further verification of uniform performance (Suppl. Fig. S9)^35,43^.

Motion artifact resilience was assessed during standardised forearm trials, comprising baseline, fist contraction, wrist and arm movements (Suppl. Fig. S10; see Methods for full protocol). Motion artifact metrics included the motion artifact index (MAI, quantifying RMS deviation during motion relative to baseline), peak-to-peak voltage during movement, and spike rate (fraction of samples exceeding 3× baseline standard deviation). Motion artifact resilience was assessed for Ag/AgCl, ETG, and gold electrodes during standardised forearm trials (Suppl. Fig. S10). Ag/AgCl electrodes exhibited moderate motion artifacts (MAI approximately 4–9, peak-to-peak voltage approximately 10–15 mV), while gold electrodes were more susceptible (MAI approximately 15–24, peak-to-peak voltage approximately 14–18 mV). In contrast, ETG electrodes demonstrated superior robustness, with lower motion artifacts (MAI approximately 2–4, peak-to-peak voltage approximately 8–12 mV), and reduced spike rates, indicating more stable recordings during movement. These results confirm that ETG arrays maintain mechanical integrity, functional electrical continuity, and robust signal quality under repeated bending and realistic motion conditions.

### Evaluation of electrode array performance

To evaluate the signal recording performance of the smart-textile fabrication method, we implemented a five-electrode linear array designed to capture multiple electrophysiological modalities, including electrocardiography (ECG), electromyography (EMG), electrooculography (EOG), and electroencephalography (EEG). These signals span a wide range of amplitudes and frequency content, from predominantly low-frequency ECG, EEG, and EOG ( < 40 Hz) to higher-frequency EMG components (up to approximately 400 Hz), such that no single electrode geometry is optimal across modalities. The array was therefore conceived as a proof-of-concept platform for multi-modal recording rather than a modality-specific optimised design.

**Fig. 2:**
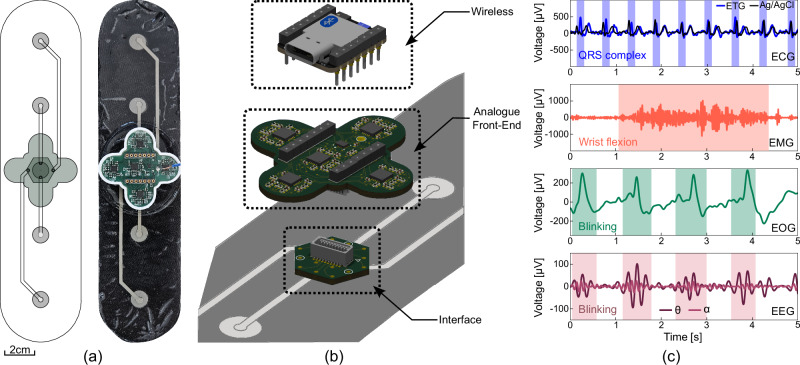
Performance evaluation via wireless multi-site electrophysiology. **a** Design and physical implementation of 5-electrode linear array used for performance evaluation. **b** 3D model and block diagram of custom wireless recording unit, consisting of three PCBs: a textile interface, an analog front-end and a bluetooth low energy transmission module. **c** Electrophysiological signals recorded from a single channel in the array. From top to bottom: electrocardiography signal from a precordial lead (blue) along with reference recording from a commercial Ag/AgCl electrode (black), electromyography signal from wrist flexion recorded on the forearm, electrooculography and electroencephalography signals (theta and alpha bands) during blinking, both recorded on the forehead.

The array (Fig. 2a) comprises five circular electrodes with a 5 mm radius and a 5 cm centre-to-centre inter-electrode distance (IED), arranged longitudinally. Five electrodes were chosen to assess variability in signal quality across positions within the array. Electrode size reflects a trade-off between signal-to-noise ratio (SNR) and spatial averaging: larger electrodes improve SNR but average spatially distributed signals, while smaller electrodes preserve local activity. A 5 mm radius is suitable for signals up to approximately 100 Hz, making it appropriate for ECG, while partially averaging higher-frequency EMG and being smaller than ideal for EEG, which could tolerate larger electrodes. This intermediate geometry enables reliable demonstration of multi-modal signal acquisition. The longitudinal layout and 5 cm IED were selected to support versatile placement across different anatomical sites, including the forehead (EEG/EOG), forearm (EMG), and precordial chest region (ECG). This design prioritises flexibility and cross-modality comparability over strict adherence to modality-specific electrode spacing guidelines (see Methods).

Conductive tracks route signals from the electrodes and converge into a hexagonal interface pattern, to which an interface PCB is bonded (see Suppl. Fig. S3). The hexagonal geometry preserves fixed IEDs through equilateral spacing of via holes and was chosen to match the structure of the interface PCB, enabling reuse with arrays of different channel counts. As shown in Fig. 2b, the underside of the interface PCB contains via holes aligned with the converging tracks, while the top side hosts a board-to-board connector for downstream electronics.

To acquire, process and transmit electrophysiological signals, a dedicated analogue front-end (AFE) PCB was designed and fabricated. The AFE PCB connects directly with the interface PCB sealed in the textile array via a matching board-to-board connector (see Suppl. Figs. S11–S15). Instrumentation amplifiers with high input impedance increase signal amplitude while minimising distortion and maximising SNR. The amplified signals are subsequently filtered using active bandpass filters (see Suppl. Fig. S16), digitised by an analogue-to-digital converter and routed to female header pins for connection to a commercially available Bluetooth low energy module. Digitised signals are transmitted wirelessly to a receiver connected to a computer running a custom user interface, enabling real-time visualisation and recording into Excel files (see Suppl. Figs. S17–S23). The system is powered by a low-profile lithium-ion battery positioned between the AFE PCB and the Bluetooth module, supporting continuous operation for up to five hours and recharging via a USB-C port.

As shown in Fig. 2c, the array successfully recorded electrophysiological signals spanning a broad range of frequencies and amplitudes. For clarity, only one representative channel is shown for each modality. Recordings were performed in separate trials using the same electrode array sequentially repositioned on different anatomical locations, as the array footprint cannot simultaneously cover all relevant body regions. For each modality, the array was placed at a location where the target signal was dominant, precordial placement for ECG, forearm placement for EMG and forehead placement for EEG and EOG, thereby naturally minimising cross-talk from other physiological sources (see Suppl. Figs. S24-S25).

From top to bottom, the figure displays precordial ECG (along with a reference Ag/AgCl control), forearm EMG recorded during 4 s of sustained 90° wrist flexion, EOG during voluntary blinking cycles and EEG activity in the theta (4–7 Hz) and alpha (8–12 Hz) bands (see Methods for more details). The ECG trace exhibits well defined P waves, QRS complexes, and T waves, indicating stable cardiac signal acquisition, with a heart rate of approximately 108 bpm estimated from R–R intervals. The SNR of the ETG textile electrode was calculated to be 19.5 dB, compared to 15.4 dB measured using a reference commercial Ag/AgCl electrode (see Fig. 2c and Methods for SNR computation). These values are consistent with previously reported performance for ETG electrodes of similar size, confirming our electrodes achieve signal quality comparable to standard clinical electrodes four times larger in size^35,43^. The EMG signal shows characteristic burst-like activity associated with muscle contraction. A processed EMG envelope is provided in Suppl. Fig. S26. EEG and EOG signals were recorded simultaneously from the forehead. Large-amplitude deflections associated with eyelid motion correspond to EOG activity, while underlying EEG activity was isolated using band-pass filtering and median detrending to suppress ocular and low-frequency artefacts (see Methods). Eye closure resulted in increased alpha and theta power, consistent with reduced visual input and cortical relaxation. Across all modalities, appropriate band-pass filtering was applied to further reduce residual cross-talk from non-target physiological signals, ensuring that the reported signals reflect the intended electrophysiological sources.

### Spatiotemporal features for enhanced classification

**Fig. 3:**
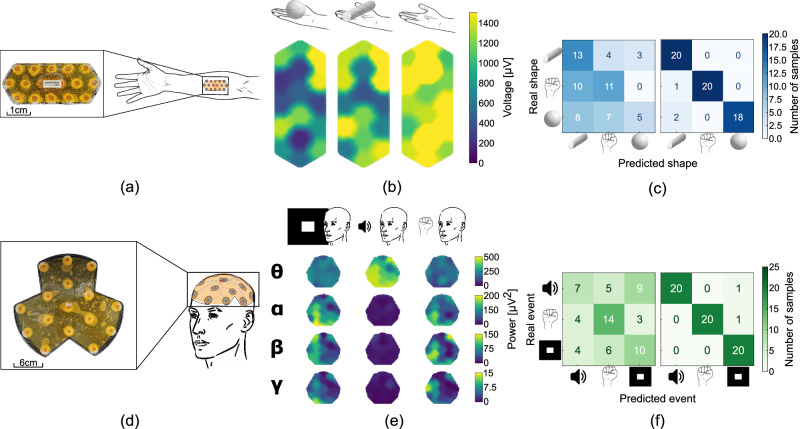
Classification of forearm EMG and scalp EEG activity using spatially distributed features. **a** Design, implementation and relative anatomical placement of the 16-channel EMG electrode array over the forearm. **b** Interpolated BSP maps of EMG envelopes for each channel during three grasp conditions: spherical grasp, cylindrical grasp, and unladen hand (left to right). **c** Confusion matrices showing classifier performance on the test set using single-electrode SNR features (left) versus spatially distributed SNR features from all 16 electrodes (right). **d** Design, implementation and placement of the 16-channel EEG electrode array configured as a headcap. **e** Interpolated BSPMs of EEG power across four canonical frequency bands, theta, alpha, beta and gamma, recorded during visual, auditory and motor stimuli (left to right). **f** Confusion matrices showing classifier performance on the test set using single-electrode EEG power features (left) versus spatially distributed power features from the full array (right).

To demonstrate the benefits of spatially distributed features from BSPM, a forearm EMG array was used to map EMG activity during object grasping tasks. The array consists of 16 circular electrodes with a 5 mm diameter and a 10 mm IED, embedded in a flexible lycra Velcro band that is aligned with the primary forearm flexor muscles (Fig. 3a and Suppl. Figs. S27, S28). The number of electrodes was set by the maximum supported by the readout circuitry. The electrode size and spacing were selected to optimally recover muscular BSP maps in accordance with established design guidelines (see Methods for details)^36^. A single participant performed three grasp conditions: spherical, cylindrical, and unladen (no object) (see Methods for experimental protocols). Each condition produced distinct spatial patterns of muscle activation. The unladen condition yielded the most uniform and consistently high-amplitude activity, suggesting unconstrained engagement of the forearm musculature. In contrast, the cylindrical and spherical grasps resulted in more heterogeneous spatial distributions, with similar activation patterns. The cylindrical grasp elicited higher amplitudes and broader spatial coverage, likely reflecting increased muscle recruitment enabled by reduced postural constraints compared to the more restrictive spherical grasp (Fig. 3b).

To evaluate the discriminative power of these spatial EMG patterns, we trained a logistic regression classifier using SNR features extracted from each electrode. As a control, an identical model was trained using SNR from only a single, centrally located electrode (see Methods). The single-channel model performed only marginally better than chance (0.33), achieving repeated cross-validation accuracy, precision, recall and F1 scores of 0.40 ± 0.08, 0.40 ± 0.14, 0.39 ± 0.08 and 0.34 ± 0.08, respectively. By contrast, incorporating SNR features from all 16 electrodes markedly improved classification performance, achieving 0.98 ± 0.02 accuracy, 0.98 ± 0.02 precision, 0.98 ± 0.03 recall and 0.98 ± 0.02 F1 score (Fig. 3c). These results demonstrate that grasp-specific EMG activation patterns are spatially distinctive and that leveraging spatial information significantly enhances decoding performance in single-subject experiments.

We applied a similar strategy to classify neural responses to visual, auditory, and motor stimuli using EEG recorded with a 16-channel array integrated into a headcap (Fig. 3d and Suppl. Figs. S29, S30). Circular electrodes are 20 mm in diameter and spaced 50 mm apart, covering frontal, central, parietal, temporal and occipital regions while leaving space to accommodate the subject’s ears. Electrode size and spacing were optimally chosen for EEG BSPM recordings in accordance with established design guidelines (see Methods for details)^36^. We filtered the EEG signals into theta (4–8 Hz), alpha (8–12 Hz), beta (12–30 Hz) and gamma (30–40 Hz) activity bands and computed band powers. Each stimulus elicited distinct spatial distributions of band power (Fig. 3e). Visual stimuli increased occipital alpha and suppressed gamma activity, consistent with alpha rebound and reduced visual processing. Auditory stimuli elicited widespread theta activity, especially in central and posterior regions. Motor activity resulted in dominant beta power over frontal and posterior areas, with focal gamma activation in the left hemisphere and residual occipital alpha activity.

For classification, frequency-domain power features from all electrodes were used to train a logistic regression model. A control model using only data from the Cz electrode performed poorly, with accuracy, precision, recall, and F1 scores of 0.45 ± 0.08, 0.45 ± 0.09, 0.43 ± 0.07, and 0.41 ± 0.07, respectively, only marginally above chance. The model trained with spatio-spectral features from all 16 electrodes achieved 0.95 ± 0.04 accuracy, 0.95 ± 0.04 precision, 0.94 ± 0.04 recall, and 0.94 ± 0.04 F1 score (Fig. 3f). These findings confirm that neural responses to sensory and motor stimuli are both spatially and spectrally distributed, and that multi-site, wearable electrode arrays can capture these patterns effectively in within-subject experiments. Additional analyses were performed to characterise the underlying electrophysiological structure of the EEG responses (Suppl. Figs. S32–S35). Event-related potential (ERP) analysis across anatomically grouped cortical regions revealed consistent but low-amplitude evoked responses ( < 0.4 µV), indicating subtle yet reproducible cortical engagement across visual, auditory and motor conditions (Suppl. Fig. S32). Spectral decomposition using periodic–aperiodic separation demonstrated structured oscillatory peaks within alpha and beta frequency ranges superimposed on a typical 1/f background, with more pronounced high-frequency components during motor activity (Suppl. Fig. S33), supporting the physiological basis of the extracted spectral features. To further distinguish time-locked versus non-phase-locked contributions, classification was repeated using evoked and induced activity separately; near-perfect performance was obtained using evoked features, whereas induced activity alone yielded substantially lower accuracy (Suppl. Fig. S34), indicating that stimulus-locked dynamics dominated decoding performance. Movement-related ERD/ERS analysis confirmed canonical motor oscillatory patterns, including pre-movement desynchronisation and post-movement rebound in alpha and beta bands (Suppl. Fig. S35), reinforcing the neurophysiological plausibility of the recorded signals.

We note that these results are based on a single participant, and future studies with additional subjects will be required to assess generalisability across individuals. To further investigate feature relevance, correlation circle analyses were performed for both EMG and EEG datasets (Supplementary Fig. S31), revealing that classification performance arises from distributed contributions across multiple channels rather than dominance by a single electrode. Electrode position can influence model performance because different electrodes capture activity from distinct muscle fibres or cortical regions, producing variations in BSP maps. During EMG measurements, array placement was maintained as consistently as possible across trials; however, small displacements of approximately 1 cm, particularly along the forearm axis, were unavoidable. Importantly, the trained algorithms remained robust to these minor positional variations, suggesting that spatially distributed features mitigate local variability in electrode placement. A similar principle applies to EEG recordings, where small shifts in cap placement did not compromise classification performance. Together, these findings indicate that leveraging spatial information across the electrode array enhances robustness to positional variability while preserving high classification performance.

### Mapping and prediction of cortico-muscular axis dynamics

We demonstrate the simultaneous use of independently optimised spatially dense cutaneous electrode arrays for concurrent acquisition of cerebral and muscular activity. This multimodal approach enables the real-time recording of brain and muscle signals during voluntary movement, offering a new paradigm for integrative electrophysiology. Because EEG and EMG signals are recorded from physically separate arrays, with independent digitisation and transmission paths, there is no cross-talk between modalities. Two anatomically distinct movements, hand flexion and wrist extension, were chosen for a controlled single-subject experiment to explore antagonistic motor control strategies and enable assessment of directional relationships between EEG spectral features and EMG spatial activation patterns. This setup underscores the versatility of the smart textile arrays and illustrates the feasibility of capturing inter-organ electrophysiological dynamics with high spatiotemporal resolution. By allowing concurrent monitoring, this system enables more complex analyses beyond conventional classification tasks, including predictive modelling of neuromuscular coordination and spatiotemporal mapping of cortico-muscular reaction times. Importantly, these analyses quantify predictive and temporal associations rather than establishing causal interactions between cortical and muscular activity, as no perturbational or interventional design was employed. Quantitative performance metrics and control analyses are provided to demonstrate that observed relationships are not attributable to shared motion artefacts or other confounding factors (see Methods and Suppl. Fig. S10).

**Fig. 4:**
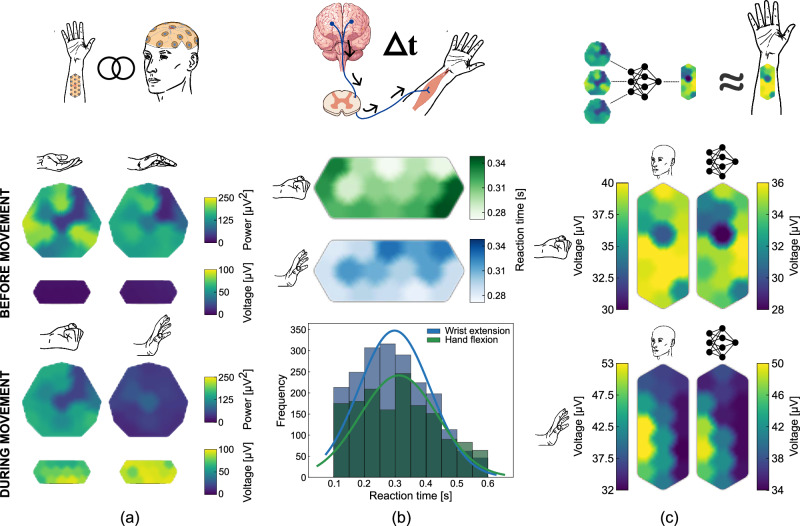
Multimodal electrophysiology for cortico-muscular mapping and prediction. **a** Top: Schematic of simultaneous EEG and EMG recordings with electrode array placement. Middle: Concurrent beta-band EEG power maps and muscular BSPMs recorded immediately before movement onset. Bottom: Same maps during movement execution. **b** Spatio-temporal analysis of reaction time from simultaneous cortical and muscular BSPM. Top: Schematic illustrating the path of motor commands from cortical planning to muscular contraction. Middle: Reaction time maps for hand flexion and wrist extension show average delays of ~300 ms and intra-array variation up to ~ 6 ms. Bottom: Histograms of reaction times across all trials with Gaussian fits for both movement types. **c** Cortico-muscular activity prediction using multimodal BSPM. Top: Schematic of prediction pipeline using PLS regression to estimate EMG maps from cortical EEG features. Bottom: Predicted vs. actual muscle activity maps for hand flexion and wrist extension.

Using a standardised task protocol (see Methods), we observed distinct activation patterns in both EEG and EMG signals across movements. Beta-band cortical activity was elevated prior to motion onset and decreased during execution, consistent with motor-related desynchronisation phenomena (EDR), supporting temporal ordering of cortical and muscular signals (Fig. 4a). EMG maps revealed stronger and more spatially diffuse responses during wrist extension, while hand flexion produced lower-amplitude, more localised activity. The lower muscular SNR observed during hand flexion is primarily due to the positioning of the array (see Suppl. Figs. S28 and S30): electrodes were placed on the right lateral side of the forearm with the palm facing up, which positions them closer to muscle fibres predominantly involved in wrist extension (extensor carpi radialis longus and brevis, extensor carpi ulnaris) than those involved in hand flexion, which are located deeper on the ventral side of the forearm. Despite targeting different joints, these antagonistic movements evoked distinguishable muscle activation maps, yet both showed similar beta desynchronisation dynamics. Spatial variations in EEG amplitude, particularly during movement planning, influenced the resulting EMG signal intensity. These findings reveal spatial relationships in cortico-muscular interactions and support the hypothesis that distinct activation sequences underlie opposing motor outputs.

Event-based cortico-muscular coherence (CMC) analysis was additionally performed using conventional magnitude-squared coherence between EEG and EMG signals, revealing significant beta-band coupling (15–30 Hz), consistent with established markers of functional communication within the motor system (Suppl. Fig. S36). Low-frequency components were excluded to minimise contamination from movement artefacts, supporting interpretation of the observed coherence as physiological rather than mechanically driven. While more advanced multivariate approaches such as canonical maximisation of coherence have been proposed to characterise interactions between multidimensional neural datasets^6^, the present analysis employed standard pairwise CMC to provide a direct and interpretable assessment of cortico-muscular coupling compatible with established literature. Importantly, rather than introducing a new coherence metric, our contribution lies in demonstrating that spatially distributed, flexible BSPM electrode arrays can capture coherent cortico-muscular dynamics with sufficient fidelity to enable downstream decoding and spatial analysis, extending beyond prior work that primarily focused on methodological optimisation of coherence estimation.

The simultaneous EEG-EMG acquisition enabled precise measurement of reaction times, the temporal delay between cortical motor planning and muscular contraction, at high resolution (Fig. 4b). Reaction times averaged between 275 and 350 ms across movements, with inter-electrode variability below 6 ms, suggesting tightly synchronised muscle activation. Spatial maps of reaction time highlighted opposing activation sequences between flexion and extension: delays clustered in inferior-lateral regions for hand flexion and in superior-central areas for wrist extension (Fig. 4b). Reaction time distributions, ranging from 100 to 600 ms, were approximately Gaussian with means near 300 ms (Fig. 4b). Broader distributions for flexion are likely due to uncertainty from lower EMG SNR and more complex recruitment patterns. These results demonstrate the utility of simultaneous multimodal electrophysiology in quantifying not just signal presence, but inter-organ timing and spatial synchrony.

To further probe cortico-muscular coupling, we employed partial least squares (PLS) regression to predict EMG activation maps from EEG cortical power features. By using spatio-spectral EEG features as inputs and EMG BSP maps as targets, the model captured inter-system signal relationships with high fidelity (Fig. 4c).

Prediction accuracy was evaluated using three complementary metrics: Pearson correlation, Spearman rank correlation, and cosine similarity. These metrics capture distinct aspects of predictive performance, quantifying linear correspondence, rank order consistency, and spatial alignment between predicted and actual EMG maps, respectively. To contextualise these results, chance-level performance was estimated by randomly permuting the EEG–EMG pairings across trials, yielding near-zero Pearson and Spearman correlations and a cosine similarity of approximately 0.5 for unit-normalised maps. Separate models for hand flexion and wrist extension achieved robust performance across test samples, with average Pearson correlations of 0.83 ± 0.20 and 0.80 ± 0.21, Spearman correlations of 0.80 ± 0.19 and 0.73 ± 0.21, and cosine similarities of 0.95 ± 0.29 and 0.99 ± 0.01, respectively. Predicted EMG maps preserved spatial activation patterns despite minor amplitude underestimation (Fig. 4c). These results demonstrate that regression-based decoding of motor commands from EEG captures genuine spatiotemporal dependencies beyond chance, providing robust predictions within a single subject. Unlike discrete classification, this approach supports continuous, high-resolution estimation of muscular output, highlighting the potential for wearable systems to infer motor intent and dynamics in real time, while acknowledging that generalisation across subjects remains to be validated.

We have reported the development of a multimodal electrophysiology platform based on a scalable smart textile fabrication process that enables the implementation of conformable and robust electrode arrays for cutaneous electrophysiology mapping. We first validated system performance through the recording of ECG, EOG, EMG, and EEG signals using a 5-electrode linear array. We then demonstrated the advanced capabilities of BSPM by enabling machine learning-based classification of object shapes and sensorimotor cues using two 16-electrode arrays optimised for EMG and EEG. Finally, by concurrently deploying independently optimised EEG and EMG arrays, we achieved simultaneous mapping of cortical and muscular activity during voluntary movement within a single subject. This approach enabled the capture of reaction time distributions along the cortico-muscular axis and prediction of spatial EMG activation patterns from cortical spectral features, illustrating the potential for regression-based decoding of motor intent. Our multimodal electrophysiology system could provide a powerful platform for investigating inter-organ physiological dynamics. Future work will focus on expanding the range of behavioural tasks and subjects, integrating electrical stimulation, assessing model generalisability across subjects and developing closed-loop neuroprosthetic feedback systems. Long-term monitoring in real-world conditions, including evaluation under laundering, perspiration exposure and temperature cycling, will also be addressed in future studies aimed at chronic deployment of this technology.

## Methods

### Fabrication of smart textile electrode arrays

A 100 µm-thick blend of lycra and nylon fabric (Nylon Lycra, UK Fabrics Online) was pre-stretched using a custom-designed frame (see Supplementary Fig. S1). Double-sided tape (152212, 3 M; 120 µm thickness) was laser-patterned into electrode geometries using a laser cutter (VLS2.30, Universal Laser Systems) and manually aligned and adhered to both sides of the fabric. Silver nanoparticle paste (LS-453-6B, Asahi Kagaku) was blade-coated over the masks using a 15 cm × 12 cm × 5 cm stainless-steel squeegee with a 1 mm metallic edge. Blade-coating was performed manually in a single pass under constant pressure, allowing the paste to permeate through the porous textile and form vertical electrical connections between both sides of the fabric. The coated fabric was cured in a standard oven at 105 °C for 30 minutes. After cooling, the electrode mask was removed from one side of the fabric, yielding a final electrode silver thickness of approximately 200 µm. A new double-sided tape mask was then applied to define the overall outline of the array.

Uncured polydimethylsiloxane (PDMS; Sylgard 184, Dow Corning; 10:1 base-to-curing-agent ratio by weight) was poured over the electrodes and spread with a squeegee to remove excess material, forming a uniform insulation layer approximately 120 µm thick and shaped by the tape mask. This PDMS layer was thermally cross-linked in an oven at 105 °C for one hour. To ensure complete electrical insulation, particularly at the edges of the silver layer, a second identical tape mask was applied and a second PDMS deposition was performed, yielding a final insulation thickness of approximately 240 µm. After curing, circular openings were rastered into the PDMS layer above the electrode sites using the same laser cutter (VLS2.30). The opening diameter was set to 50% of the electrode diameter to tolerate laser-to-mask misalignment on the order of hundreds of micrometres without compromising electrical access to the electrode.

A low-tack, silicone release agent-free surface protection tape (“Bluetape”, 1009R-6.0, SPS Ltd; 70 µm thickness) was laser-patterned with holes matching the rastered openings and applied to the PDMS surface. Silver epoxy (8330S-21G, MG Chemicals) was applied to the exposed openings using a swab and blade-coated to ensure complete filling. The tape mask was then removed and the epoxy cured in an oven at 105 °C for 30 min.

A second laser-patterned Bluetape mask was applied to define the conductive track layout. Silver nanoparticle paste was blade-coated onto the exposed PDMS surface using the same stainless-steel squeegee and cured at 105 °C for 30 min, resulting in conductive tracks with a thickness of approximately 60 µm. To prevent delamination and improve mechanical robustness under bending, uncured PDMS was carefully brushed over the tracks, leaving the central contact pads exposed, and subsequently cross-linked at 105 °C for one hour.

A third patterned Bluetape mask was applied to expose the central pads, and silver epoxy was swabbed onto the exposed regions. After mask removal, the epoxy was cured at 105 °C for 30 min, producing contact pads with a thickness of approximately 70 µm. A fourth patterned Bluetape mask, identical to the third, was then applied, and silver nanoparticle paste was blade-coated over the epoxy pads. While still uncured, a custom interface PCB was manually aligned and placed on top to ensure contact between the PCB pads and the device interface. The silver paste was subsequently cured at 105 °C for 30 min.

A final contour-defining double-sided tape mask was applied to guide a final PDMS deposition that sealed the PCB and reinforced the conductive tracks against bending-induced stress. This PDMS layer had a thickness of approximately 2 mm. After thermal cross-linking at 105 °C for one hour, all tape masks were removed, yielding an overall device thickness of 2 mm ± 0.5 mm.

To improve electrode–skin coupling, a conducting polymer eutectogel (ETG) composed of PEDOT:LS, glycerol/choline chloride (Gly:ChCl), and gelatin was deposited on the opposite side of the textile. The ETG material was solvent-free and processed by melt deposition. ETG was melted into approximately 3 mm-thick sheets and cut using a 100 µm-thick stainless steel mould matching the electrode geometry, providing a mass of approximately 0.15 g of ETG per 10 mm diameter electrode (approximately 0.00191 g/mm^−2^). Each ETG piece was placed directly onto the corresponding electrode site and heated in an oven at 105 °C for 10 minutes. Due to the hydrophobic nature of the ETG, the material melted and reflowed into a stable, drop-like geometry confined to the electrode area, without lateral spreading or leakage beyond the electrode boundary. Upon cooling, the ETG solidified to form a conformal, mechanically stable coating. The resulting ETG protrusion above the textile surface was measured using a stylus profilometer (Veeco Dektak 6 M Stylus Surface Profilometer), yielding a height of 1 mm ± 5 µm.

For single-layer designs, flexible printed circuit boards (FPCBs; PCBWay) can replace blade-coated silver tracks. In this configuration, bottom-side pads on the FPCB align with the Ag epoxy vias above the textile electrodes, while the top side houses the required connectors or circuitry. An additional layer of silver epoxy is applied to both the textile contact pads and the FPCB pads, and the assembly is co-cured at 105 °C for 30 minutes to establish electromechanical connections. A final layer of uncured PDMS is applied across the assembly to seal the external connector and enhance mechanical stability.

Reliable electrical performance was confirmed throughout fabrication and use by continuity testing and by verifying stable electrophysiological recordings following repeated handling, applied pressure, and device attachment, ensuring robust electrical integrity under realistic experimental conditions.

### Synthesis of PEDOT:LS ETG coating material

Choline chloride-glycerol DES was prepared by mixing ChCl and Gly at a 1:2 molar ratio. The mixture was heated to 90 °C under continuous magnetic stirring until a clear and homogeneous liquid was obtained. Upon cooling to room temperature, the DES remained liquid and exhibited an ionic conductivity of 2.6 mS cm^−1^.

PEDOT:LS was synthesised via oxidative chemical polymerisation of 3,4-ethylenedioxythiophene (EDOT) in the presence of lignin sulfonate (LS), following a previously reported procedure^50^. Polymerisation was carried out at room temperature for 8 h using iron(III) chloride as a catalyst and ammonium persulfate as the primary oxidant, with an initial EDOT:LS weight ratio of 90:10. The resulting PEDOT:LS dispersion was freeze-dried to obtain a dark-blue powder for subsequent processing.

ETGs were prepared by dispersing PEDOT:LS powder (2% w/v) into the ChCl/Gly DES (2:1 molar ratio) and mixing until homogeneous. Gelatin (20% w/v) was then added, and the mixture was heated to 90 °C under stirring to ensure complete dissolution and uniform incorporation. The resulting solution was cooled and stored at 4 °C overnight to promote gelatin triple-helix formation through hydrogen bonding, yielding a mechanically stable PEDOT:LS-based eutectogel suitable for electrode coating.

### Mechanical testing and cyclic bending

Mechanical compliance and robustness of the gel-free smart textile electrode arrays were evaluated using four-point bending tests performed on a Tinius Olsen 1st testing machine. A 4 mm displacement corresponded approximately to the MBR for silver-track devices (0.5 mm radius). Devices incorporating FPCB tracks exhibited higher bending stiffness than silver-track devices, while both configurations remained within accepted limits for wearable applications. The MBR was quantified by gradually displacing devices over metal rods of defined radii (10, 8, 6, 5, 4, 3, 2, 1, and 0.5 mm) until loss of electrical performance and fracture were observed after 10 s of holding. Cyclic bending tests were conducted at radii slightly exceeding the MBR to represent subcritical loading conditions relevant to repeated physiological deformation (2 mm radius, κ = 500 m^−1^, for laminated silver-track devices; 3 mm radius, κ = 333 m^−1^, for FPCB-based devices). Devices were manually bent for 100 cycles, with each bending and relaxation phase lasting approximately 1 s. Electrical continuity was verified using a standard DC multimeter connected via a custom PCB breakout board, with testing performed every 10 cycles. Three independent devices were tested per configuration for three different measurements, and no evidence of delamination, cracking, or loss of continuity was observed (Suppl. Figs. S5–S7).

### Electrical performance and impedance testing

Electrical continuity of all electrodes was confirmed during fabrication and following mechanical tests. To provide additional verification, the impedance of ETG-coated electrodes was measured on human skin at 50 Hz using a potentiostat (Autolab). Five independent devices were measured, each with ten repeated recordings across 19-electrode hexagonal arrays. Average impedance values at 50 Hz with a median of 0.427 MΩ and an interquartile range (IQR) of 0.529 MΩ across 19-electrode hexagonal arrays, with electrode-to-electrode variability of approximately 69.7 kΩ (Suppl. Fig. S9). These measurements are consistent with previously published characterisation of ETG electrodes, providing additional confirmation of uniform electrical performance.

### Motion artifact characterisation

Motion artifact resilience was assessed for Ag/AgCl, ETG, and gold electrodes during standardised forearm trials covering the flexor carpi ulnaris and radialis. The trial consisted of sequential phases of baseline (10 s), fist contraction (10 s), wrist side-to-side movement (10 s), arm up-and-down movement (10 s), erratic circular arm movements (10 s), and recovery (10 s) (Suppl. Fig. S10). For each electrode type, three independent devices were tested, with three recordings per device. Motion artifact metrics were computed from raw voltage traces, with baseline defined as the initial 10 s. Key metrics included:

- Motion Artifact Index (MAI): standard deviation during motion divided by baseline standard deviation.
- Peak-to-peak voltage: maximum voltage excursion during motion phases.
- Spike rate: fraction of samples exceeding three times baseline standard deviation.

ETG electrodes demonstrated superior robustness, with MAI of approximately 2–4, peak-to-peak voltages of approximately 8–12 mV, and spike rates of 0.16–0.21. Ag/AgCl and gold electrodes were measured as controls. Ag/AgCl electrodes exhibited intermediate artifacts (MAI 4–9, peak-to-peak 10–15 mV, spike rate 0.26–0.56), while gold electrodes were most susceptible (MAI 15–24, peak-to-peak 14–18 mV, spike rate 0.37–0.45). These results confirm that ETG arrays maintain mechanical integrity, functional electrical continuity, and robust signal quality under repeated bending and realistic motion.

### Environmental conditions and reproducibility

All mechanical and motion tests were performed at room temperature without controlled humidity. Data were collected across multiple devices and trials, and all quantitative metrics are reported as mean ± standard deviation to provide a clear representation of variability and reproducibility.

### Interpretation framework and causal assumptions

Analyses relating EEG spectral features to EMG spatial activation were designed to identify statistical associations between cortical and muscular signals rather than establish causal relationships. Reaction-time measurements and EEG-to-EMG mapping analyses therefore quantify temporal and correlational relationships only. No directed connectivity models, causal inference frameworks, or perturbation-based validation were employed.

To reduce potential confounds arising from shared motion artefacts, preprocessing steps included bandpass filtering, exclusion of low-frequency components dominated by movement artefacts, and comparison across electrode materials with different motion sensitivities. Consequently, results should be interpreted as evidence of predictive relationships within a controlled single-subject framework rather than proof of causal neural drive.

### Computation of optimal electrode array parameters

Optimal electrode array parameters for 16-channel EMG and EEG arrays were computed strictly following the design framework and analytical procedure described in ref. ^36^. All assumptions, parameter choices and calculations reported here directly implement the instructions provided in that reference.

Circular electrodes were selected, as prescribed in ref. ^36^ to account for non-uniform spatial propagation of bioelectric fields and to minimise orientation-dependent effects.

For EMG recordings, a maximum signal frequency of 400 Hz was assumed, consistent with the known spectral content of surface EMG^63^. For EEG recordings, the maximum frequency of interest was set to 40 Hz, encompassing the canonical theta, alpha, beta and low-gamma bands analysed in this study^64^. Electrode diameters were chosen using the selection criteria specified in ref. ^36^, to balance SNR and spatial averaging effects, yielding optimal values of 5 mm for EMG and 20 mm for EEG.

Inter-electrode distance (IED) was calculated using the spatial sampling criterion outlined in ref. ^36^ to avoid spatial aliasing while preserving spatial resolution. Calculations assumed an average conduction velocity of 4 m/s for bioelectric signal propagation along the skin surface, in accordance with prior literature^36^. Based on these assumptions, the resulting optimal IEDs were 10 mm for EMG and 50 mm for EEG.

### Recording support electronics

For the experiments shown in Figs. 3, 4, electrophysiological signals were recorded using an RHS Stim/Recording System (INTAN Technologies) at a 30 kHz sampling rate. Signals were acquired in a monopolar configuration, with the reference and ground shorted and connected to a commercial electrode (Meditrace) placed over a bony region. The INTAN front-end provided analogue bandpass filtering and gain adjustment; hardware filtering settings were chosen to accommodate the wide frequency range required for EEG and EMG recordings. IntanRHX Software v3.2.0 was used for data collection.

For Fig. 2, recordings were acquired using a custom wireless PCB at a 1 kHz sampling rate (Supplementary Figs. S5–S17). This system was also configured in a monopolar montage with reference and ground shorted to a commercial electrode placed in a bony area. The wireless PCB includes analogue bandpass filtering (0.5–100 Hz) and gain adjustment prior to digitisation. The lower sampling rate was chosen to match the bandwidth requirements of ECG signals (its original intended use) and to reduce power consumption for wireless operation.

### Experimental protocols and data collection

All experiments were conducted on a single healthy adult male participant. Recordings shown in Fig. 2 were obtained from a participant in his 20 s, while those in Figs. 3–4 were obtained from a participant in his 40 s. Ethical approval was obtained in accordance with University of Cambridge requirements, and informed consent was provided prior to participation. Repeated trials were used to assess within-subject consistency rather than inter-subject variability; quantitative metrics reported in the Results (e.g. SNR, impedance, band power) are averaged across trials.

Figure 2 (multimodal signal acquisition):

For all recordings in Fig. 2, the same five-electrode linear textile array was repositioned across anatomical sites in separate trials. To ensure stable placement and consistent skin contact across these distinct locations, the array was affixed using a medical-grade adhesive film (3 M 152212, 120 µm thickness). No conductive gels were used.

For ECG recordings, the array was positioned longitudinally over the precordial region, approximately spanning V2–V6. A commercial Ag/AgCl reference electrode (Meditrace) was placed on a bony region at the right ankle to minimise interference. Three 2 min recordings were acquired while the participant was seated at rest with controlled breathing.

For EMG recordings, the array was placed circumferentially around the forearm, over the extensor muscle compartment, with placement guided by palpation during voluntary contraction. A commercial reference electrode was placed posterior to the elbow on a bony region. The participant rested the arm on a table while seated, with the right palm facing upward. Following a 10 s baseline period, the participant performed a single sustained 90° wrist flexion lasting 4 s, followed by 5 s of rest. Three trials were recorded. The task timing is explicitly annotated in Fig. 2, and a processed EMG envelope is provided in Suppl. Fig. S26.

For EOG/EEG recordings, the array was positioned longitudinally along the forehead. A commercial reference electrode was placed on a bony area behind the ear. The participant was instructed to blink naturally at regular, self-paced intervals (approximately 500 ms per blink, with ~1 s between blinks) for 30 s, followed by 30 s of rest, across three 2 min trials. EEG and EOG were recorded simultaneously at this location. Appropriate bandpass filtering was applied to minimise cross-talk from non-dominant physiological signals.

In all Fig. 2 experiments, the adhesive fixation ensured that the array moved with the body segment rather than sliding on the skin. No visible electrode detachment or lateral displacement was observed during recordings.

Figure 3 (EMG BSPM during grasping and EEG responses to sensory and motor stimuli):

For EMG recordings in Fig. 3, a 16-electrode EMG array was positioned over the forearm flexor–extensor region, with placement guided by palpation during voluntary contraction to ensure coverage of the primary muscles involved in grasping. The array was secured using a Velcro strap that applied uniform circumferential pressure. No adhesives or conductive gels were used. The strap mechanically coupled the array to the forearm, minimising relative displacement during movement. The participant performed three grasping conditions: spherical grasp, cylindrical grasp, and unladen (imaginary) grasp. Each condition consisted of 10 trials, with alternating 5 s contraction and 5 s rest periods, separated by 10 s rest intervals between trials.

For EEG recordings in Fig. 3, a separate 16-electrode EEG array was integrated into a textile headcap and secured using laces to provide uniform pressure across the scalp. No adhesives or gels were used. EEG recordings were performed in independent trials from the EMG experiments. After a 10 s baseline period, three types of stimuli were presented: visual, auditory, and motor. Visual stimuli consisted of a white rectangular shape displayed on a black background; auditory stimuli consisted of a single musical note played through speakers; motor stimuli consisted of voluntary fist closure. Each stimulus was presented for 2 s, followed by 2 s of rest, and each condition was repeated for 10 trials of 10 cycles each. Stimulus presentation and timing were controlled using custom scripts.

Figure 4 (simultaneous EEG–EMG during motor tasks):

For Fig. 4, EMG arrays were secured to the forearm using Velcro straps, while the 16-electrode EEG array was integrated into a headcap with laces to provide uniform pressure across the scalp (similarly to Fig. 3). No adhesives or gels were used. Sustained hand flexion was performed for 2 s followed by 2 s of rest for 10 cycles, across 10 trials. A similar protocol was followed for 90° wrist extension. EEG and EMG recordings were acquired simultaneously. Task design avoided large shear forces at the electrode–skin interface, and the arrays remained mechanically stable throughout the protocol.

Across all experiments, electrode fixation relied on either adhesive films (Fig. 2) or pressure-based attachment (Figs. 3–4), ensuring consistent skin contact while allowing the devices to adjust to body movements. Motion artefact robustness was further evaluated through dedicated tests reported in Supplementary Fig. S10.

### Preprocessing of recorded electrophysiological signals

All electrophysiological signals were processed offline using custom scripts (Python). Raw data were visually inspected prior to analysis to verify signal integrity and identify gross artefacts. Unless otherwise stated, all filters were implemented as zero-phase, fourth-order Butterworth filters to avoid phase distortion.

For all modalities except EOG, a 50 Hz notch filter was applied to suppress power-line interference. Signals were then detrended using a median filter to remove slow baseline drift.

ECG signals were band-pass filtered between 0.5 and 100 Hz to preserve P waves, QRS complexes, and T waves while attenuating low-frequency motion artefacts and high-frequency noise. Heart rate was estimated from R–R intervals detected using a peak-finding algorithm applied to the filtered signal.

EMG signals were band-pass filtered between 5 and 400 Hz, full-wave rectified, and subsequently smoothed using a moving-average window of 500 samples to generate EMG envelopes. The envelope was used for temporal alignment and qualitative comparison of muscle activation patterns; a representative envelope is provided in the Supplementary Information.

EOG signals were band-pass filtered between 0.5 and 10 Hz to isolate slow ocular potentials associated with blinking and eye movements. No notch filtering was applied to EOG to avoid distorting low-frequency blink dynamics.

EEG signals were band-pass filtered into canonical frequency bands: theta (4–8 Hz), alpha (8–13 Hz), beta (13–30 Hz), and gamma (30–40 Hz). For spectral analysis, a fast Fourier transform (FFT) was applied to segmented epochs after filtering, and band power was computed by integrating the power spectral density within each frequency range. Median detrending was applied prior to band-pass filtering to reduce low-frequency ocular and motion-related artefacts.

For all modalities, identical preprocessing pipelines were applied across trials and conditions to ensure consistency and comparability.

All signal processing, feature extraction, and statistical analyses were performed using custom scripts written in Python (v3.10). External auditory and visual stimuli for EEG experiments were generated using a dedicated custom script (“External stimuli.ipynb”), ensuring precise timing control during stimulus presentation. Data processing and analysis were implemented in additional custom scripts (“Wireless_BSPM.ipynb” and “Additional_figures.ipynb”). All custom code is publicly available at 10.5281/zenodo.20041637.

### EEG signal processing and analytical procedures

#### Evoked Response Potentials (ERPs)

Continuous EEG data were acquired at 30 kHz, down-sampled to 1 kHz and preprocessed to remove artifacts. Epochs were defined relative to stimulus onset, spanning −200 to +800 ms. Stimuli consisted of repeated cycles of 2 s stimulation followed by 2 s rest, yielding 100 pseudo-trials per condition. Electrodes exhibiting excessive noise (A-004, A-025, A-030, A-031) were excluded.

Channels were grouped according to anatomical regions (Anterior Frontal, Frontal, Central, Temporal, Parietal, Occipital). Within each region, trial-wise data were averaged across channels to isolate regional responses. ERPs were computed as the mean across trials, and variability was quantified using the standard error of the mean (SEM). This approach captures phase-locked neural activity associated with sensory and motor stimuli.

#### Spectral decomposition and periodic vs. aperiodic components

Power spectral densities (PSDs) were estimated using Welch’s method with 2 s windows and 50% overlap over the 1–100 Hz range. For each stimulus, channel spectra were averaged to obtain a global PSD. To separate oscillatory (periodic) activity from the broadband (aperiodic) background, the spectra were parameterized using the FOOOF algorithm, which decomposes log-power spectra into an aperiodic 1/f component, characterized by slope and offset, and oscillatory peaks representing canonical neural rhythms. Model parameters were set with peak width limits of 1–12 Hz, a maximum of six peaks, and a minimum peak height of 0.1. This approach enables independent quantification of oscillatory activity while avoiding confounds from broadband spectral shifts.

#### Evoked vs. induced activity

Phase-locked and non-phase-locked neural responses were separated by first extracting epochs for all trials. The ERP was calculated as the trial-average waveform, with evoked activity defined as the flattened ERP. Induced activity was obtained by subtracting the ERP from individual trials, thereby removing phase-locked components while retaining trial-specific oscillatory variability. Feature matrices derived from both evoked and induced activity were subsequently used for multiclass classification using multinomial logistic regression (lbfgs solver, maximum 1000 iterations). Classification performance was evaluated using five-fold cross-validation, providing confusion matrices and per-stimulus as well as overall accuracies.

#### Event-Related Desynchronisation/Synchronisation (ERD/ERS)

Motor-related ERD/ERS was quantified from sensorimotor EEG channels. Movement events were detected automatically by identifying peaks in the broadband envelope, smoothed with a Gaussian kernel (σ = 0.2 s). Epochs spanning −2 to +4 s relative to event onset were extracted.

Band-limited signals were obtained using fourth-order Butterworth filters in canonical alpha (8–12 Hz) and beta (13–30 Hz) frequency bands. Instantaneous power was calculated via the Hilbert transform, log-transformed, and averaged across channels. Power was baseline-normalized using the interval −1.5 to −0.5 s relative to movement onset:

1 $${ERD}/{ERS}=\frac{P(t)-{P}_{{{\rm{baseline}}}}}{{{\rm{| }}}{P}_{{{\rm{baseline}}}}{{\rm{| }}}}\times 100$$

Trial-averaged time courses and standard deviations were computed for each frequency band, providing robust estimates of task-related desynchronization and synchronization.

All analyses were implemented in Python using standard scientific libraries. Channel selection, filtering, Hilbert transformation, and spectral parameterisation followed fixed parameters across trials and conditions. Random seeds were fixed to ensure reproducibility, and all feature extraction pipelines were validated against raw data to confirm accuracy of ERP, ERD/ERS, and spectral decomposition metrics.

### Feature extraction

All feature scaling and dimensionality reduction steps were performed using parameters derived exclusively from the training data within each cross-validation fold to avoid data leakage.

EMG and ECG SNR were computed per channel as the ratio of signal power during contraction to noise power during rest, expressed in decibels. Power was computed as the mean squared amplitude of the processed waveform, and SNR was calculated as:

2 $${{\rm{SNR}}}({{\rm{dB}}})=10{\log }_{10}\left(\frac{\sum {{\rm{| }}}s{{{\rm{| }}}}^{2}/N}{\sum {{\rm{| }}}n{{{\rm{| }}}}^{2}/N}\right)$$

where s and n are the signal and noise segments of equal length.

EEG band power was computed after bandpass filtering (0.5–100 Hz) and resampling to 1 kHz. Welch’s method (2-s non-overlapping windows) was used to estimate power spectral density, and band power was obtained by integrating the PSD within canonical frequency bands: delta (0.5–4 Hz), theta (4–8 Hz), alpha (8–13 Hz), beta (13–30 Hz), and gamma (30–100 Hz). The first 10 s of each recording were excluded as baseline.

Reaction time was defined as the latency between peak pre-movement beta activity and EMG onset, where EMG onset was detected using a threshold above baseline noise.

### Feature importance analysis using correlation circles

To explore the contribution of spatially distributed features to classification performance, correlation circles were generated using principal component analysis (PCA) applied to the feature matrices used for classification. Feature vectors (per-electrode SNR for EMG and band-power features for EEG) were standardised to zero mean and unit variance prior to analysis.

PCA was performed on the standardised feature matrix, and correlation circle coordinates were computed from the loading vectors scaled by the square root of the corresponding eigenvalues. These coordinates represent the correlation between each original feature and the principal component axes. Features positioned near the unit circle indicate strong representation within the dominant variance structure.

Correlation circles were used as exploratory visualisations to assess spatial feature importance and identify electrodes contributing most strongly to variance relevant to classification (Suppl. Fig. S31). These analyses complement the classification results by providing qualitative insight into spatial feature contributions rather than formal feature selection.

### Machine learning model descriptions

The logistic regression model used in this study was implemented using the LogisticRegression class from the scikit-learn library. The model estimates the probability that an input sample belongs to each class based on a linear combination of features:

3 $$P\left(y=1 | x\right)=\frac{1}{1+{e}^{-({w}^{{{\top }}}x+b)}}$$

where w is the weight vector and b is the bias term. The predicted class label is obtained by applying a threshold of 0.5 to the output probability. Model parameters were optimised by minimising the regularised logistic loss. Hyperparameters (regularisation type and strength) were selected to balance model complexity and generalisation.

Performance was assessed using repeated stratified k-fold cross-validation (10 folds, 5 repeats), with accuracy, precision, recall, and F1-score reported.

### PLS regression (EEG → EMG mapping)

Partial least squares regression (PLSR) was implemented using the PLSRegression class from scikit-learn. PLSR extracts latent components that maximise covariance between the predictor matrix X (EEG features) and the response matrix Y (EMG maps). The model iteratively computes weight vectors w and c, latent scores $t={Xw}$ and $u={Yc}$, and loading matrices p and q, such that the covariance between t and u is maximised. Assuming a linear relationship $u={b}\; {t}$, the final model can be expressed as:

4 $$\hat{Y}={XW}{({P}^{T}W)}^{-1}{Q}^{T}=X{B}_{{\mbox{PLS}}}$$

where W, P, and Q are weight and loading matrices, and ${B}_{{\mbox{PLS}}}$ is the regression coefficient matrix. Model performance was evaluated using a 20% hold-out test set and reported using ${R}^{2}$ and RMSE.

### Machine learning model training

All machine learning models were trained and evaluated using an 80:20 train-test split. For classification tasks (EMG-based shape prediction and EEG-based action prediction), stratified sampling ensured proportional representation of all classes in both training and testing sets. Model performance was further assessed using 10-fold cross-validation repeated five times on the full dataset, with metrics averaged across folds and repetitions. No hyperparameter tuning beyond default values was performed.

For regression tasks (EEG-to-EMG mapping), a standard 80:20 train-test split was applied, and model performance was evaluated on the held-out test set. Predictive accuracy was quantified using coefficient of determination (R²), root-mean-square error (RMSE), Pearson correlation, Spearman rank correlation, and cosine similarity, computed for each test sample and averaged across the set.

Random seeds were fixed to ensure reproducibility. This approach ensures robust evaluation while minimizing potential biases due to class imbalance (for classification) or sample ordering (for regression).

### Cortico-muscular coherence analysis

Cortico-muscular interactions between EEG and EMG signals were evaluated using frequency-domain coherence analysis. EEG and EMG signals recorded simultaneously during motor tasks (Fig. 4) were segmented into task-aligned epochs corresponding to active movement periods. Prior to analysis, EEG and EMG signals were bandpass filtered using the preprocessing pipeline described above and resampled to a common sampling rate.

Magnitude-squared coherence was computed using Welch’s method, estimating cross-spectral density between EEG and EMG channels using fixed-length windows with 50% overlap. Coherence spectra were obtained as:

5 $${C}_{{xy}}\left(f\right)=\frac{{{\rm{| }}}{S}_{{xy}}(f){{{\rm{| }}}}^{2}}{{S}_{{xx}}(f){S}_{{yy}}(f)}$$

where ${S}_{{xy}}(f)$ is the cross-spectral density between EEG and EMG signals and ${S}_{{xx}}(f)$, ${S}_{{yy}}(f)$ are the corresponding auto-spectral densities.

Analysis focused on the beta-band (15–30 Hz), which is commonly associated with cortico-muscular coupling during sustained motor activity. Standard pairwise coherence was employed rather than multivariate canonical coherence approaches (e.g., canonical maximisation of coherence) to preserve direct interpretability of spatial contributions from individual electrodes and maintain consistency with the spatial BSPM framework used throughout this study.

CMC analysis was performed primarily to characterise spectral relationships between modalities rather than to infer directional or causal interactions.

### Classification and performance evaluation

Logistic regression models were used to classify EMG and EEG conditions in Fig. 3, either from single-channel features or full-array feature sets. Classification performance was quantified using accuracy, precision, recall, and F1 score, computed for each fold of repeated 10-fold cross-validation and reported as mean ± standard deviation. Confusion matrices were generated to visualise class-wise performance.

For regression analyses in Fig. 4, EEG-derived predictions of EMG maps were evaluated on the held-out test set. Performance metrics included Pearson correlation, Spearman rank correlation, and cosine similarity, computed for each test sample across all EMG channels and reported as mean ± standard deviation. R² and root-mean-square error (RMSE) were additionally calculated to quantify goodness-of-fit.

### Ethics

Every experiment involving human participants have been carried out following a protocol approved by an ethical commission. Each participant gave informed written consent. On-body human trials were conducted at the University of Cambridge, UK, in compliance with the Ethics Committee of the Department of Engineering (06/09/2018).

## Supplementary information

Supplementary Information

Transparent Peer Review file

## Source data

Source Data

## Data Availability

All data supporting the findings of this study are available within the article and its supplementary files. Any additional requests for information can be directed to, and will be fulfilled by, the corresponding author. Source data are provided with this paper. The code used to process and analyse the data in this study is publicly available at 10.5281/zenodo.20041637. The electrophysiological datasets generated in this study consist of human EEG and EMG recordings and are not publicly available due to ethical and data protection constraints, including participant consent limitations and compliance with applicable data privacy regulations. Access to a de-identified minimum dataset sufficient to reproduce the analyses may be granted upon request for non-commercial research purposes, subject to institutional data-sharing agreements and ethical approval where required. Requests should be directed to the corresponding author. Applicants must provide a brief research proposal outlining the intended use of the data. Requests are typically reviewed within 2–4 weeks. Approved users will be granted access to the data for a defined period and under conditions that prohibit re-identification or onward sharing. Source data are provided with this paper.

## Code availability

The codes used for collecting data are available from the corresponding author upon reasonable request.
