# Supplementary material for: Body surface potential mapping of the cortico-muscular axis using smart textile electrode arrays
Source: Nat Commun. 2026 Jul 8;17:8441. doi: 10.1038/s41467-026-75134-1 (PMC13478180; doi:10.1038/s41467-026-75134-1)
Supplement: Supplementary file 1 [file 41467_2026_75134_MOESM1_ESM.pdf]

Supporting information

*Ruben Ruiz-Mateos Serrano, Charlie Brunt, Xudong Tao, Maciej Zajaczkowski, Antonio Dominguez-Alfaro, Matias L. Picchio, Daniele Mantione, Emmanuel M. Drakakis, David Mecerreyes, George G. Malliaras**

R. Ruiz-Mateos Serrano, C. Brunt, X. Tao, A. Dominguez-Alfaro, G. G. Malliaras
Electrical Engineering Division, University of Cambridge, Cambridge, CB3 0FA, UK
E-mail: gm603@cam.ac.uk

M. Zajaczkowski, E.M. Drakakis
Department of Bioengineering, Faculty of Engineering, Imperial College London, London, W12 7TA UK

M. L. Picchio, D. Mantione, D. Mecerreyes
POLYMAT, University of the Basque Country UPV/EHU, Avda. Tolosa 72, Donostia-San Sebastian, Gipuzkoa, 20018 Spain

IKERBASQUE, Basque Foundation for Science, Bilbao, 48009 Spain

Reference	Modality	Electrode Materials	Fabrication Method	Multimodal (Y/N)	Simultaneous recording (Y/N)	Multi-electrode array (Y/N)	Multi layers / Vias (Y/N)
[1], 2006	ECG	Stainless steel yarns	Knitting	N	N	N	N
[2], 2006	ECG	Silver-coated polyester yarn	Embroidery	N	N	N	N
[3], 2007	EMG	Silver-based yarn	Sewing	N	N	Y	N
[4], 2007	ECG + Respiration + Motion	Stainless steel threads and textile yarns.	Knitting	Y	Y	N	N
[5], 2009	ECG	Silver and gold textile electrodes	Sewing/Printing	N	N	N	N
[6], 2009	ECG	Silver-coated yarns	Knitting	N	N	N	N
[7], 2010	EMG	Stainless-steel yarn	Knitting	N	N	Y	N
[8], 2010	ECG + Bio-impedance	Silver paste	Screen printing	Y	N	Y	Y
[9], 2011	ECG	Silver-plated nylon conductive thread	Sewing	N	N	N	N
[10], 2011	ECG + Motion	Cu-sputtered textile; Cu/Ni plated fabric;	Sputtering, electroless plating,	Y	N	N	N

		stainless-steel yarn	knitting, embroidery				
[11], 2012	EEG	Silver-plated yarn	Knitting	N	N	N	N
[12], 2013	ECG	Silver-coated yarn	Knitting	N	N	N	N
[13], 2013	EOG	Silver-coated nylon textile	Sewing	N	N	N	N
[14], 2014	ECG + EMG + EOG	Silver paste	Screen printing	Y	Y	Y	N
[15], 2014	EMG + EOG	Silver polymer paste	Screen printing	Y	Y	Y	N
[16], 2014	ECG	Polypyrrole–coated cotton	Polymerization on textile	N	N	N	N
[17], 2015	ECG	Reduced graphene oxide	Dip coating	N	N	N	N
[18], 2015	ECG	PEDOT:PSS solution	Brush coating	N	N	N	N
[19], 2015	EOG	Silver/polyamide dry fabric electrodes	Sewing	N	N	N	N
[20], 2015	EEG	Conductive paste	Screen printing	N	N	N	N
[21], 2015	ECG	Ag/Ti-coated yarn	Embroidery	N	N	N	N
[22], 2016	EMG	Silver-plated copper yarn	Sewing	N	N	N	N
[23], 2016	ECG	Silver-plated conductive textile	Sewing	N	N	N	N
[24], 2016	ECG	PEDOT:PSS-based cotton	Sewing	N	N	N	N
[25], 2017	ECG	Graphene oxide + Ag ink	Inkjet printing	N	N	N	N
[26], 2017	EEG	Ag/AgCl-coated thread	Knitting	N	N	Y	N
[27], 2017	ECG	PEDOT:PSS	Inkjet printing	N	N	N	N
[28], 2018	EMG	Silver yarn	Knitting	N	N	N	N
[29], 2018	EMG + EEG	Ag-based composite	Inkjet printing	Y	N	N	Y
[30], 2018	EOG	Graphene oxide coated textile	Dip coating	N	N	N	N
[31], 2018	EMG	Silver-plated yarn	Knitting	N	N	Y	N
[32], 2018	ECG	PEDOT:PSS-coated knitted textile/ Silver-coated yarn	Dip coating/Sewing	N	N	N	N
[33], 2018	ECG	Silver-plated chitosan fabrics	In situ reduction of silver ions on fabrics	N	N	N	N

[34], 2018	ECG + Motion	Ag-plated yarn	Knitting	Y	Y	N	N
[35], 2018	ECG	Ag ink/PEDOT:PSS on textile	Screen printing	N	N	N	N
[36], 2018	EMG	Medtex P-130 conductive fabric	Sewing	N	N	N	N
[37], 2021	ECG	Silver/carbon yarns & Carbon/PEDOT:PSS based inks	Knitting & Screen printing	N	N	N	N
[38], 2022	EMG	Silver ink / PEDOT:PSS	Screen printing / Dip coating	N	N	Y	Y
[39], 2022	ECG + EMG	CNT-based nonwoven fabrics	Electrospinning + Dip coating + Spray coating	Y	N	N	N
[40], 2022	ECG	PEDOT:PSS coated cotton textile	Drop coating	N	N	N	N
[41], 2022	ECG	Reduced graphene oxide on textile	Spray printing	N	N	N	N
[42], 2023	EMG	Polyester conductive thread (CleverTex)	Embroidery	N	N	N	N
[43], 2023	ECG	Graphene-based composite on textile	Casting on fabric	N	N	N	N
[44], 2023	ECG	EGaIn on textile	Dip-coating	N	N	N	N
[45], 2023	ECG + EMG + sweat sensing	EGaIn + Au	Spray coating	Y	N	N	N
[46], 2024	ECG + EMG	PEDOT:PSS/PEO composite ink	Screen printing	Y	Y	Y	N
[47], 2025	EEG	HC-12 conductive Madeira thread	Embroidery	N	N	Y	N
[48], 2025	ECG + IPG	Silver-based conductive yarn	Knitting	Y	Y	Y	N
This work	ECG + EMG + EOG + EEG	Silver nanoparticle paste / PDMS	Screen printing	Y	Y	Y	Y

Table S1. Comparison of textile-based electrode platforms for electrophysiological biopotential monitoring

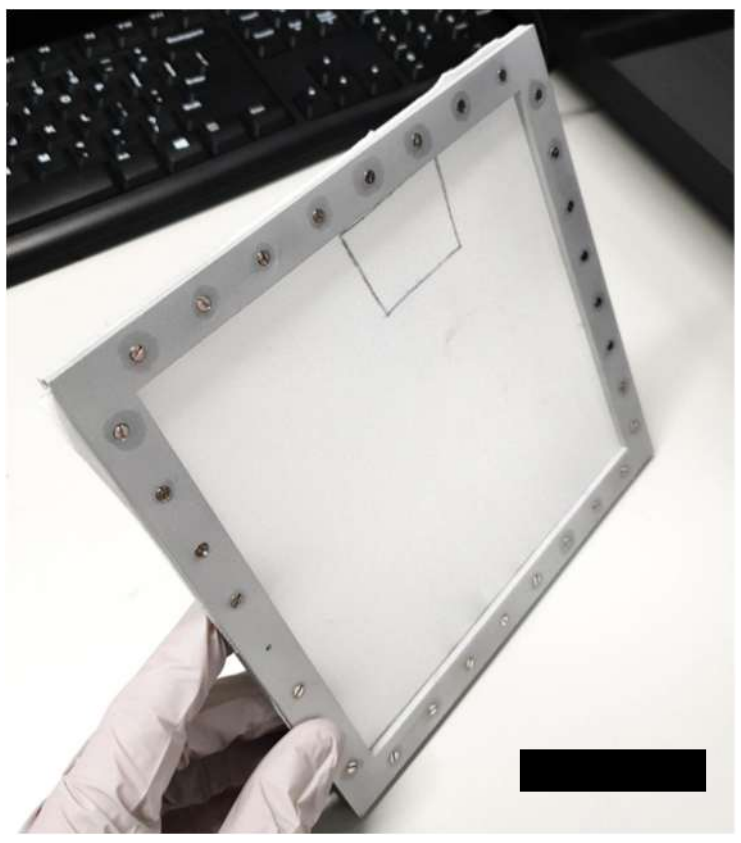

Figure S1. Stainless steel pin frame used for uniform textile pre-stretching. Scale bar = 5cm

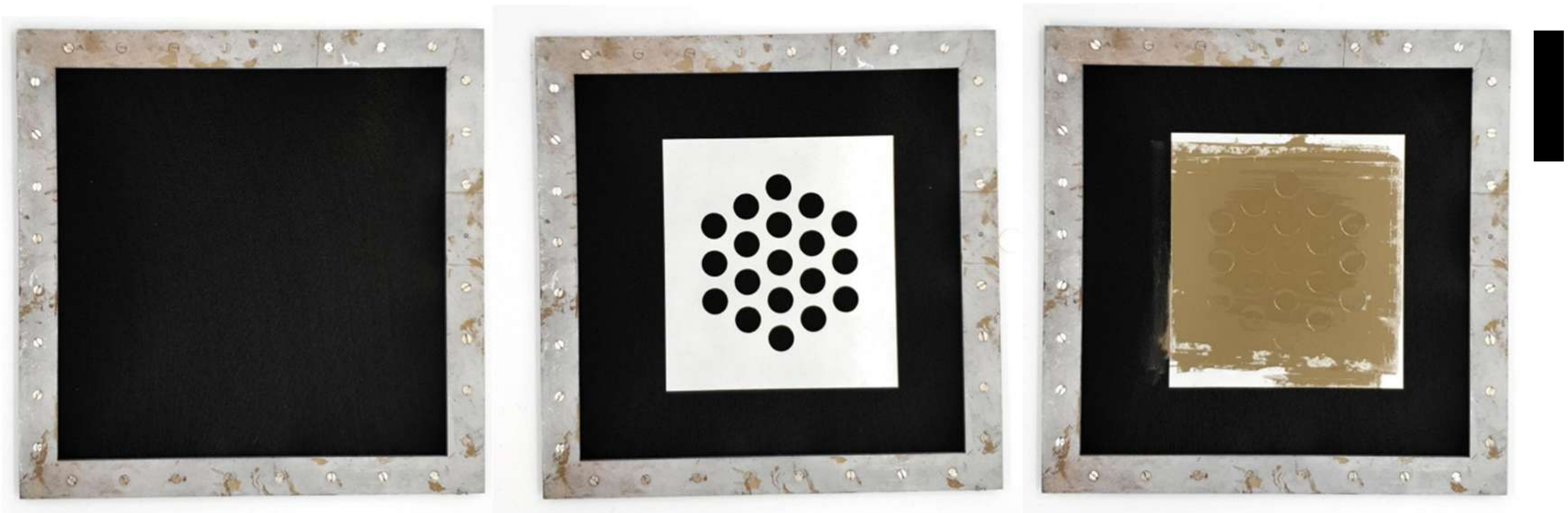

Figure S2. Double-sided medical grade laser-patterning mask for blade-coating. Scale bar = 3.5cm

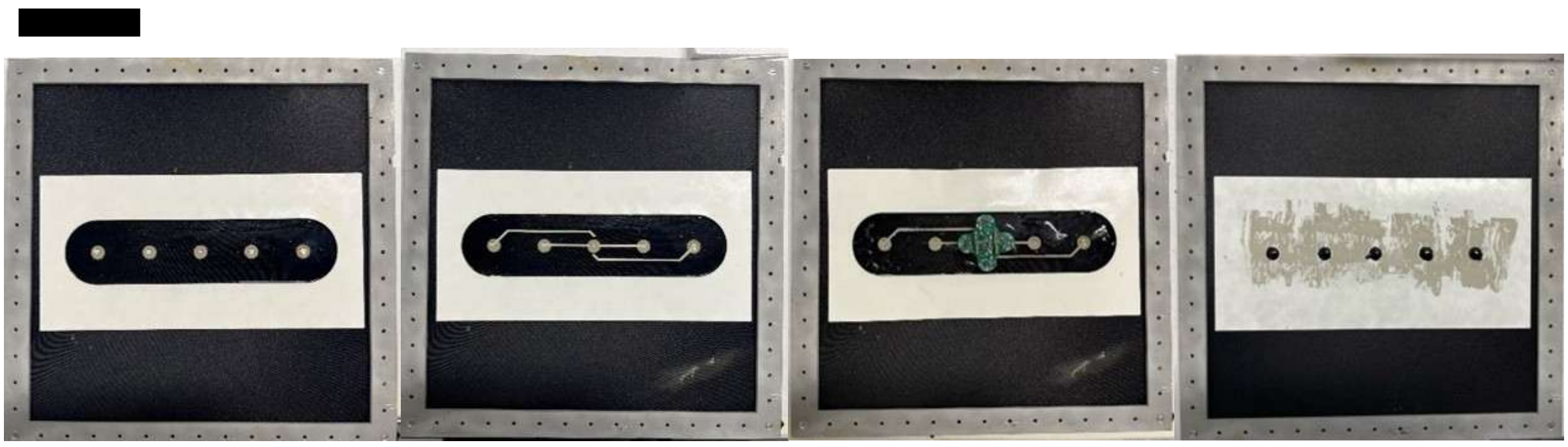

Figure S3. Fabrication steps for 5 channel linear electrode array. From left to right, silver ink deposition of electrodes, silver ink screen-printing of tracks, interface PCB placement and eutectogel electrode coating deposition. Scale bar = 10cm

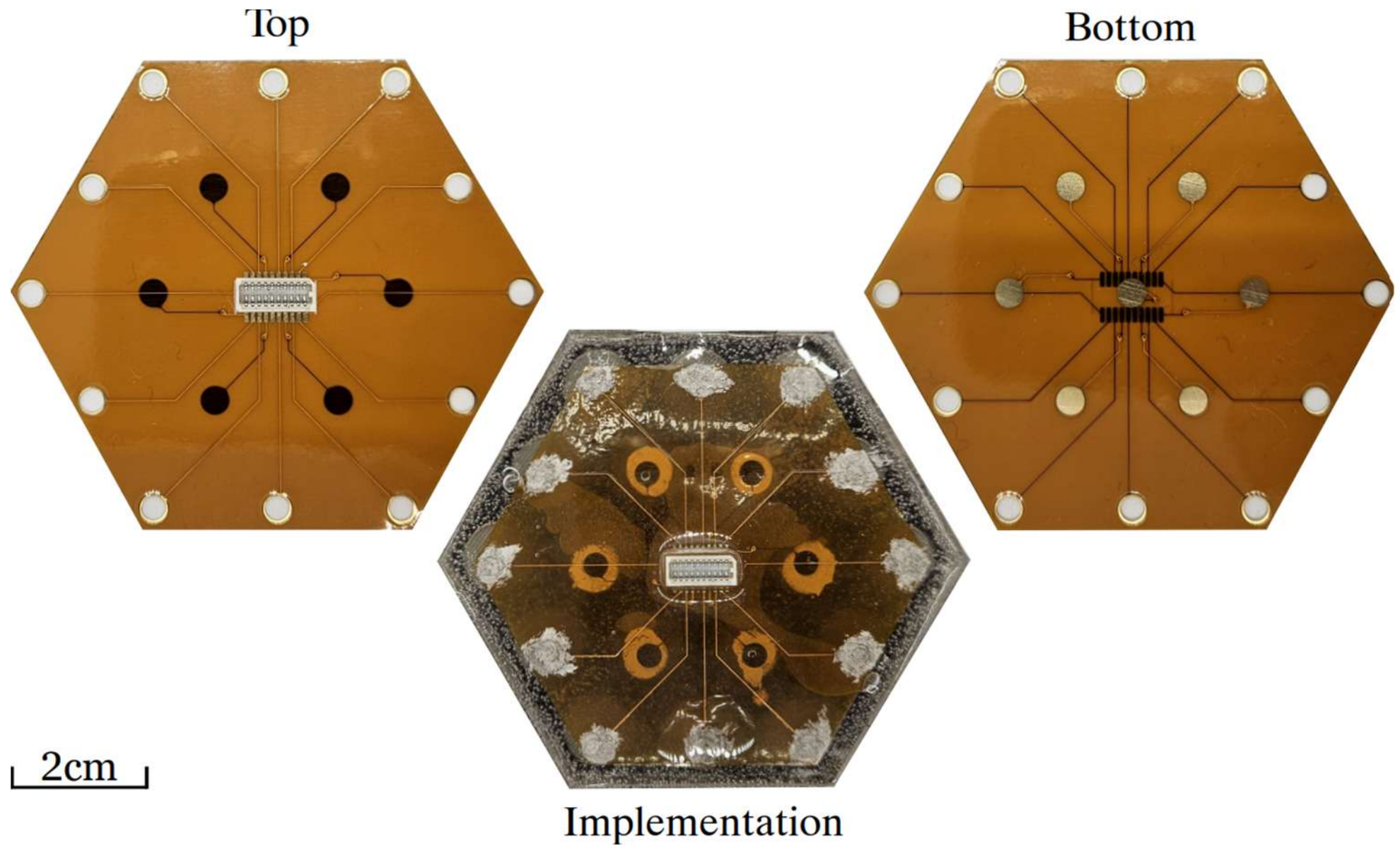

Figure S4. Flexible PCB implementation of the e-textile fabrication process

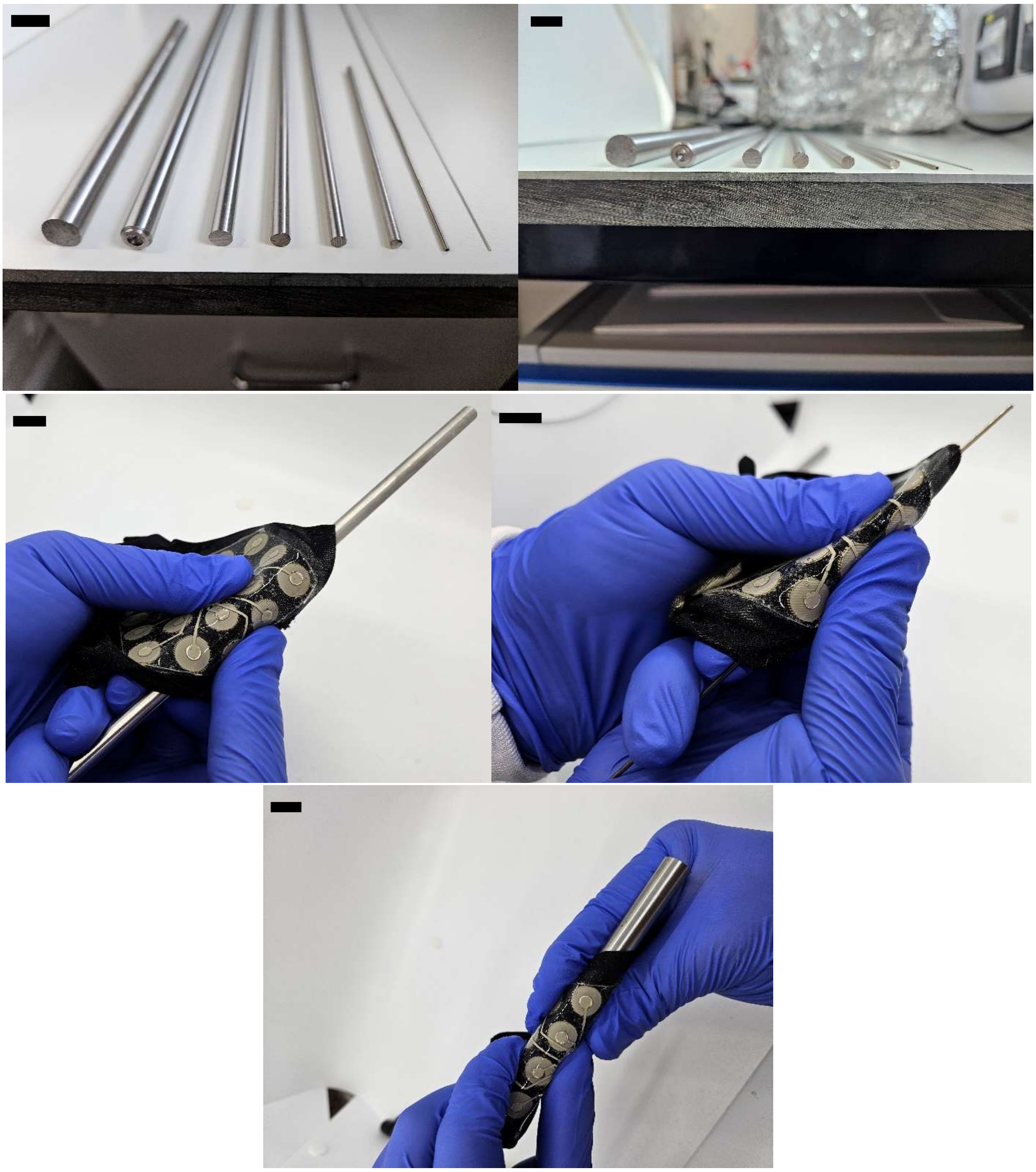

Figure S5. Experimental setup for minimum bending radius (MBR) and cyclic bending tests. Metal rods of defined radii were used to quantify the conformability and mechanical robustness of flexible devices. The applied radii (left to right) were 10 mm, 8 mm, 6 mm, 5 mm, 4 mm, 3 mm, 2 mm, 1 mm, and 0.5 mm. Scale bar = 1 cm.

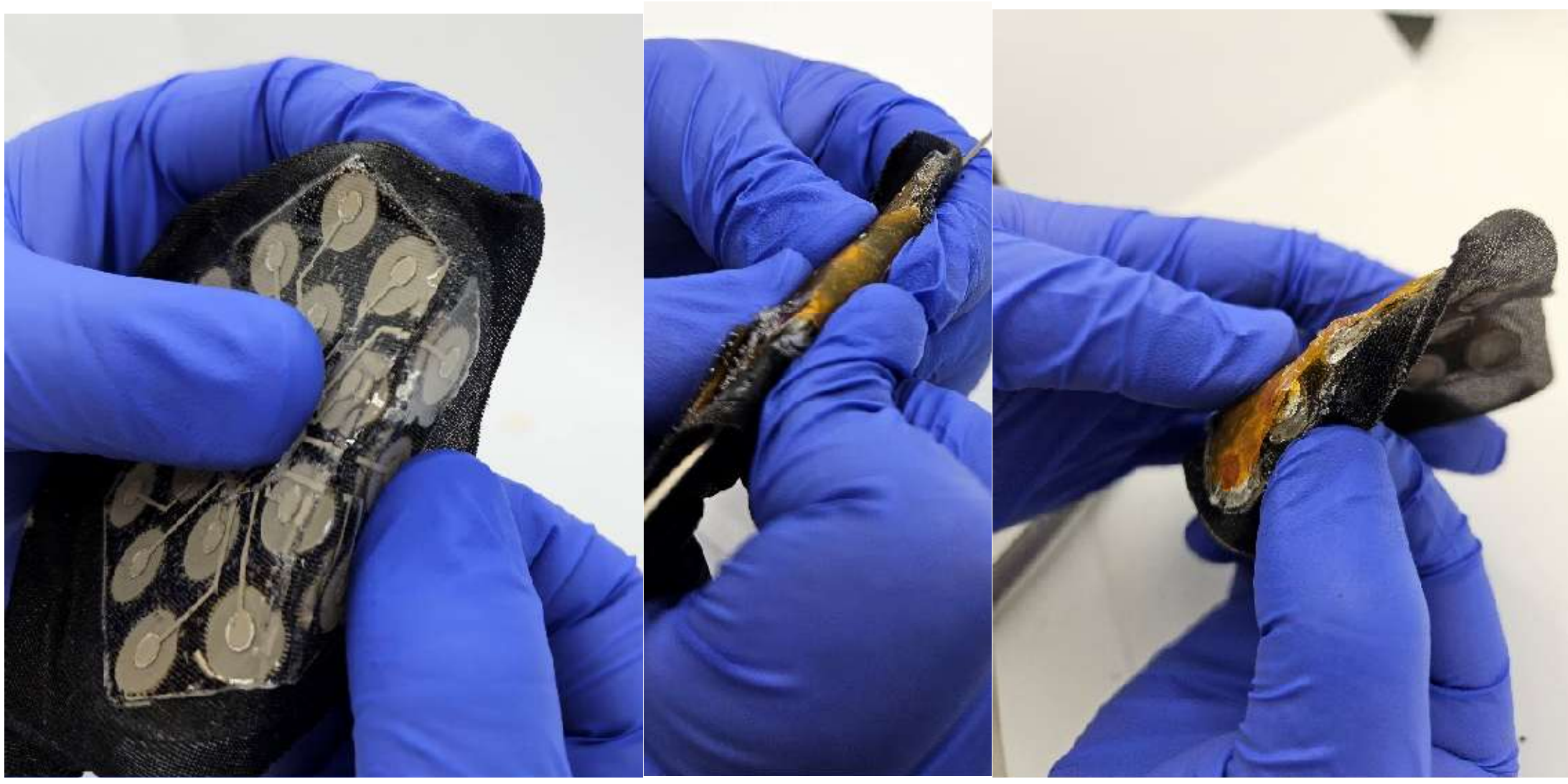

Figure S6. Examples of fracture or delamination during minimum bending radius testing

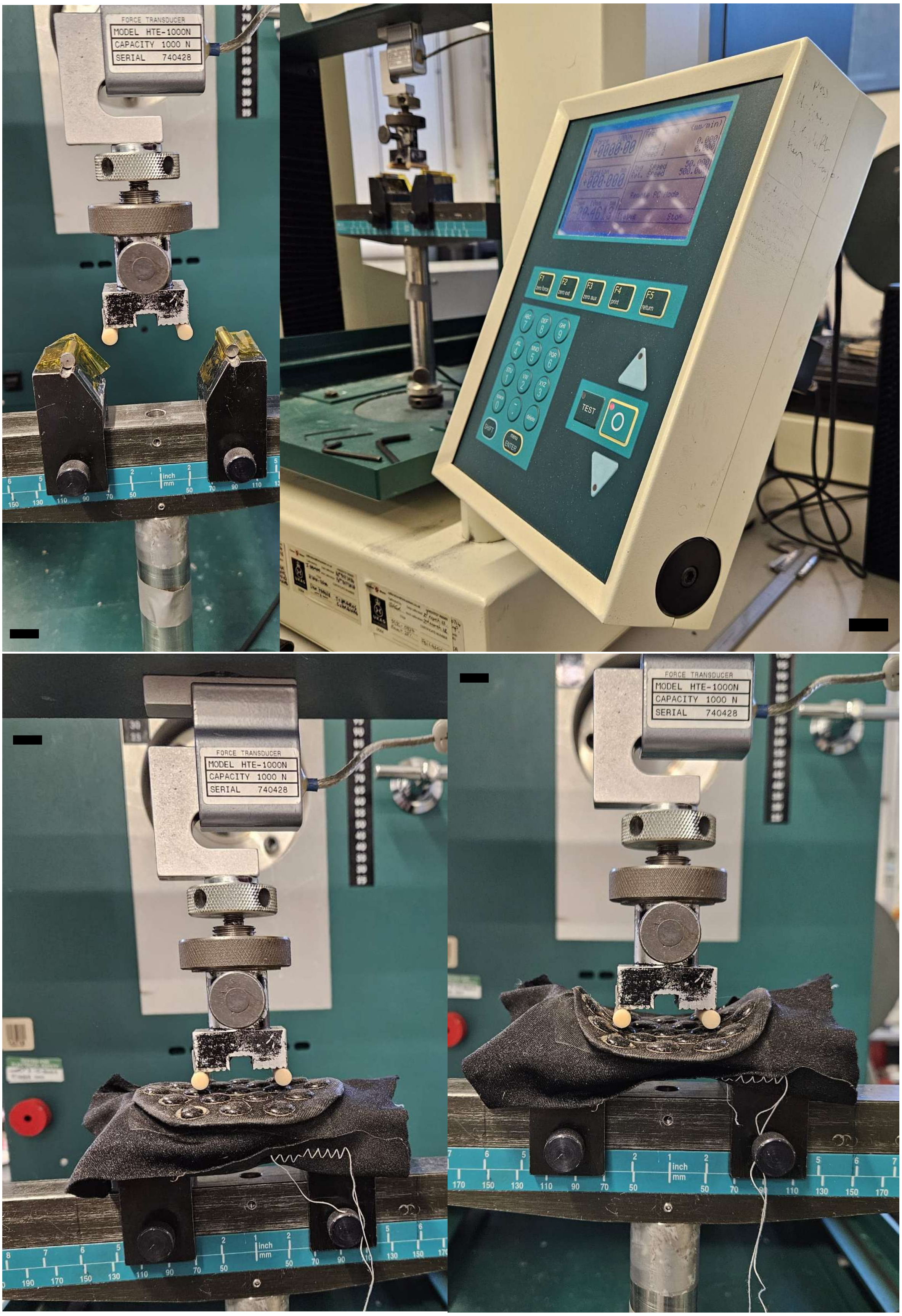

Figure S7. Setup and example of four-point bending tests performed to assess the conformability of the textile technology. Scale bar = 1cm

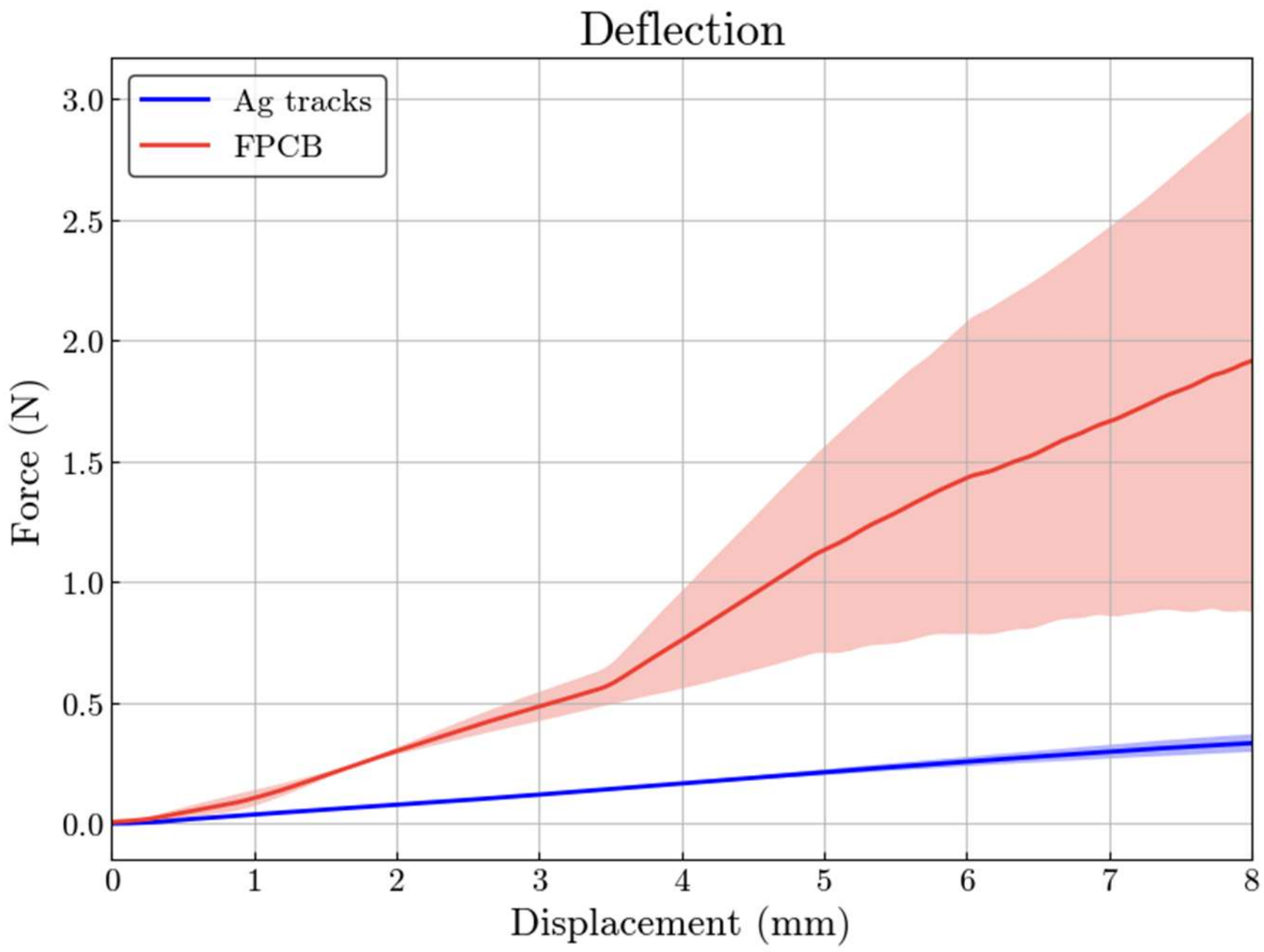

Figure S8. Deflection results from 4-point bending testing. Devices incorporating fPCB tracks exhibit higher bending stiffness relative to silver tracks, while both device types remain within accepted mechanical limits for wearable applications. A displacement of 4 mm corresponds approximately to the minimum bending radius observed for silver track devices (0.5 mm)

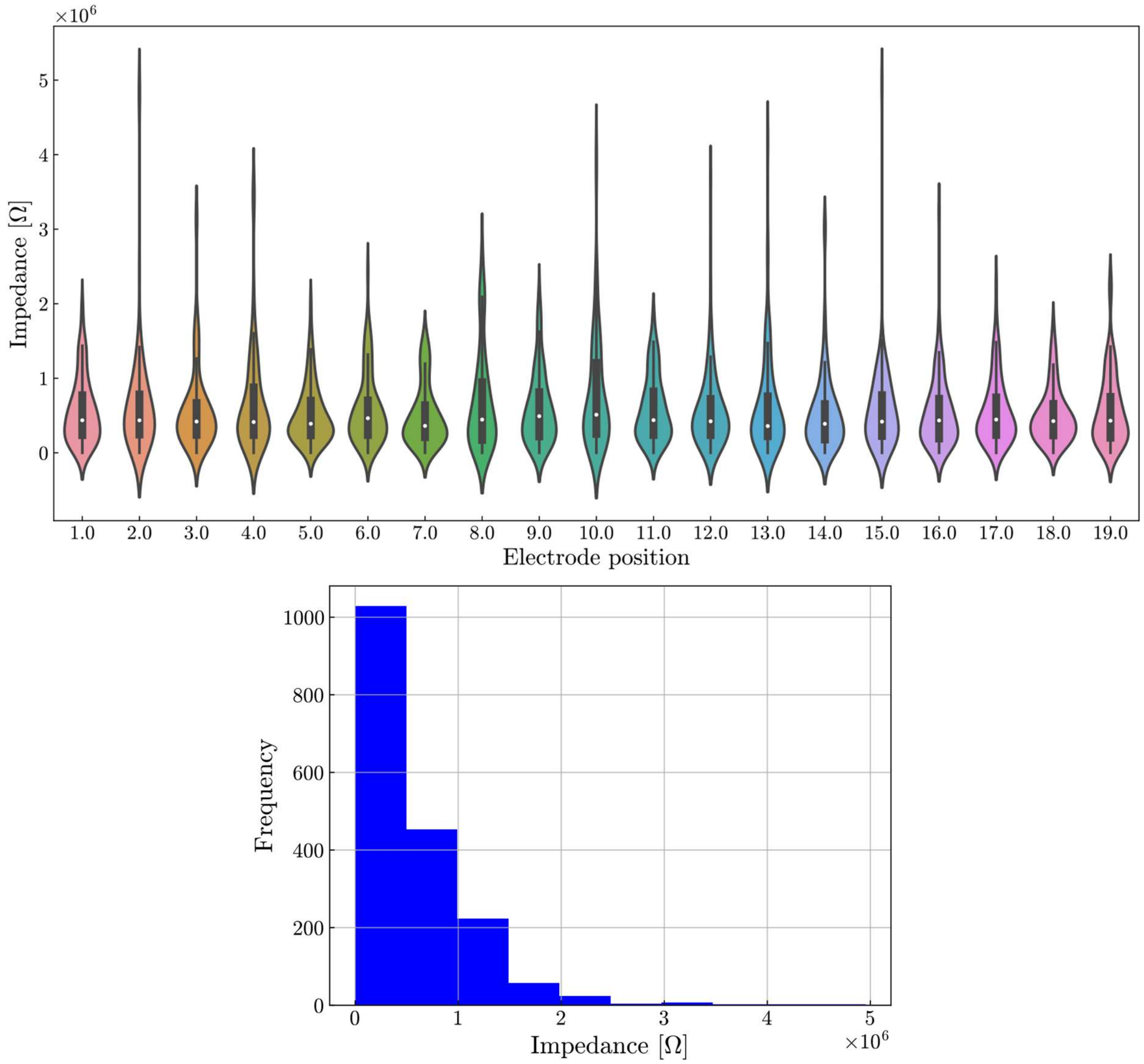

Figure S9. Impedance characterisation of 19-electrode hexagonal devices. Top: Violin plots showing per-electrode impedance distributions across five devices, with ten repeated measurements per device, illustrating electrode-to-electrode and device-to-device variability. Bottom: Histogram of all impedance measurements combined, showing the overall distribution across electrodes, measurements and devices

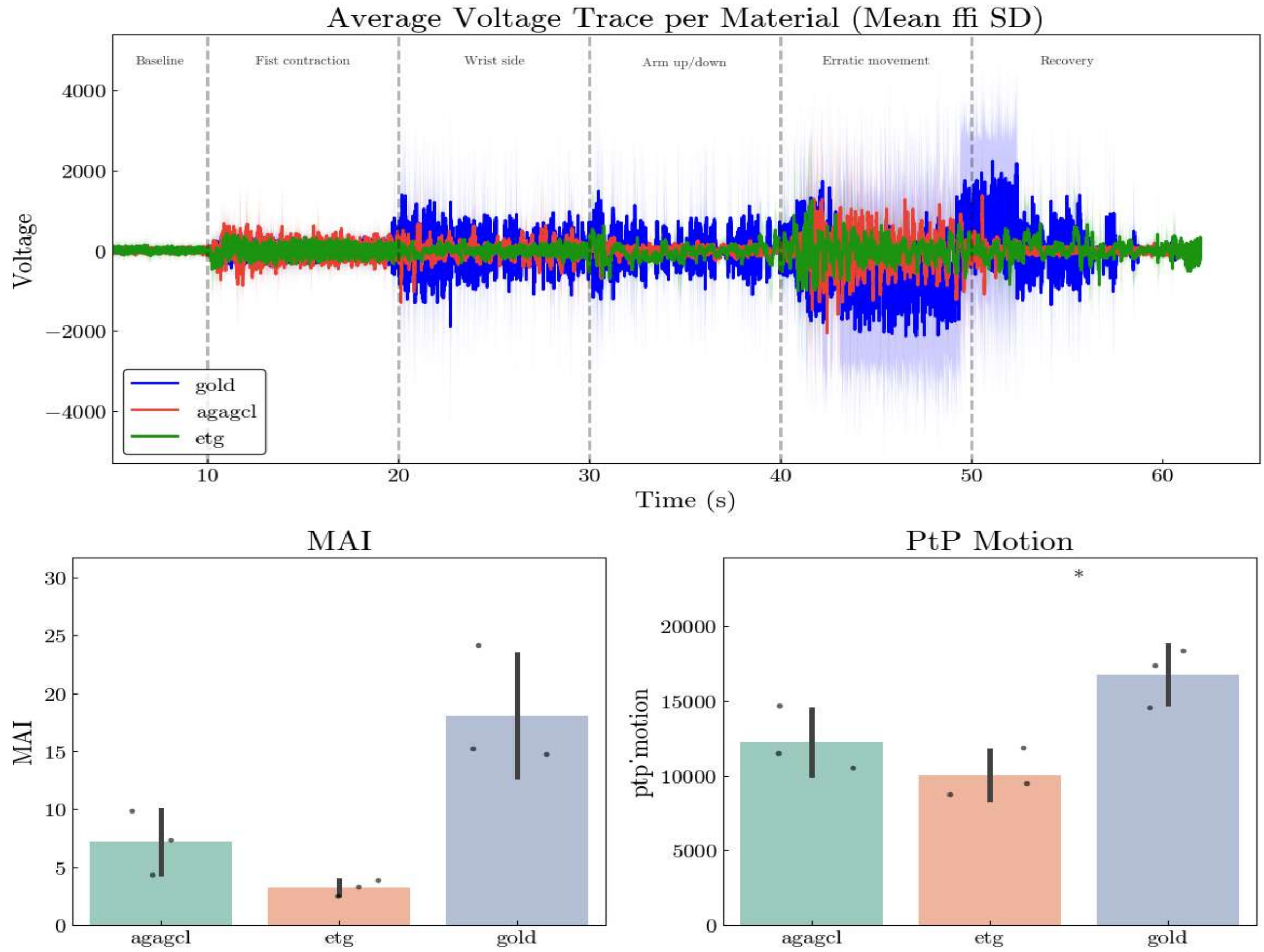

Figure S10. Motion artifact analysis of different electrode materials. Top: Average voltage traces for each material during the standardised forearm trial. Shaded areas indicate ±1 standard deviation across devices and measurements. The trial consisted of sequential phases: baseline (10 s), fist contraction (10 s), wrist side-to-side movement (10 s), arm up-and-down movement (10 s), erratic circular arm movements (10 s) and recovery (10 s). Phase boundaries are indicated with vertical dashed lines and labelled segments. Bottom: Motion artifact metrics derived from the recordings: Motion Artifact Index (MAI), peak-to-peak voltage during motion, and spike rate (fraction of samples exceeding 3× baseline standard deviation). Bars show the mean across devices, with error bars representing the standard deviation. Individual data points are overlaid as dots. Pairwise statistical comparisons between materials are indicated with connecting lines and significance symbols: + $p<0.1$, * $p<0.05$, ** $p<0.01$, *** $p<0.001$ (n=9). Lower values indicate better resilience to motion artifacts

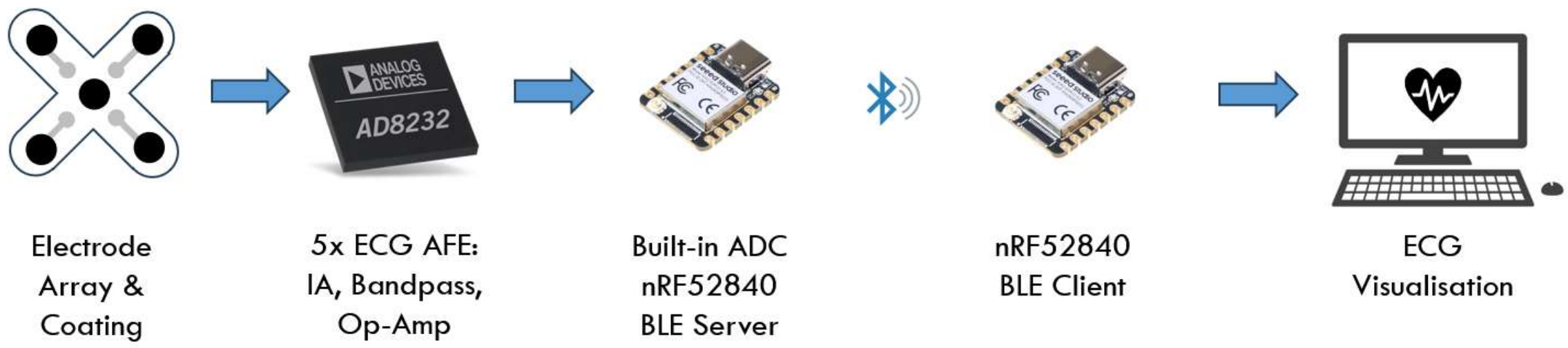

Figure S11. High-level block diagram for linear 5 electrode array. From left to right: electrode array connects to ECG-specific analogue front-end electronics, data is digitised and sent via Bluetooth Low Energy with a custom wireless unit and the information is displayed in a monitor

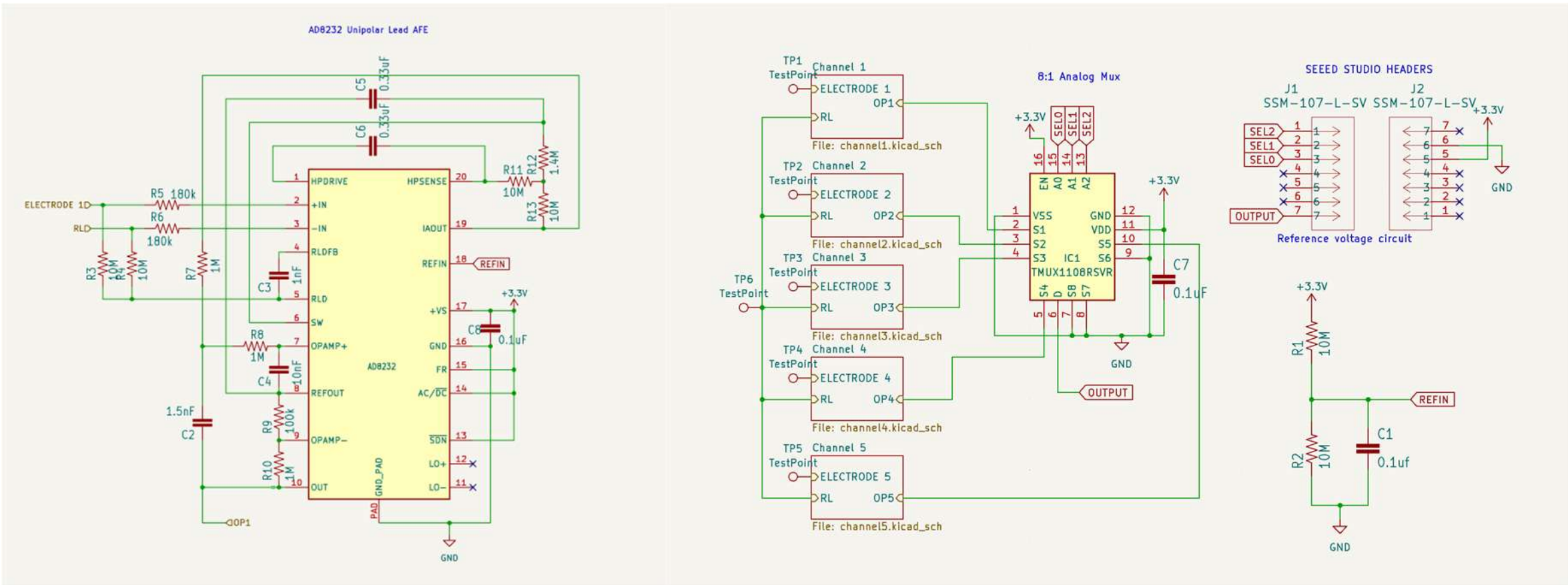

Figure S12. Analogue front-end schematic for a single ECG channel; five multiplexed channels, development board socket and reference supply

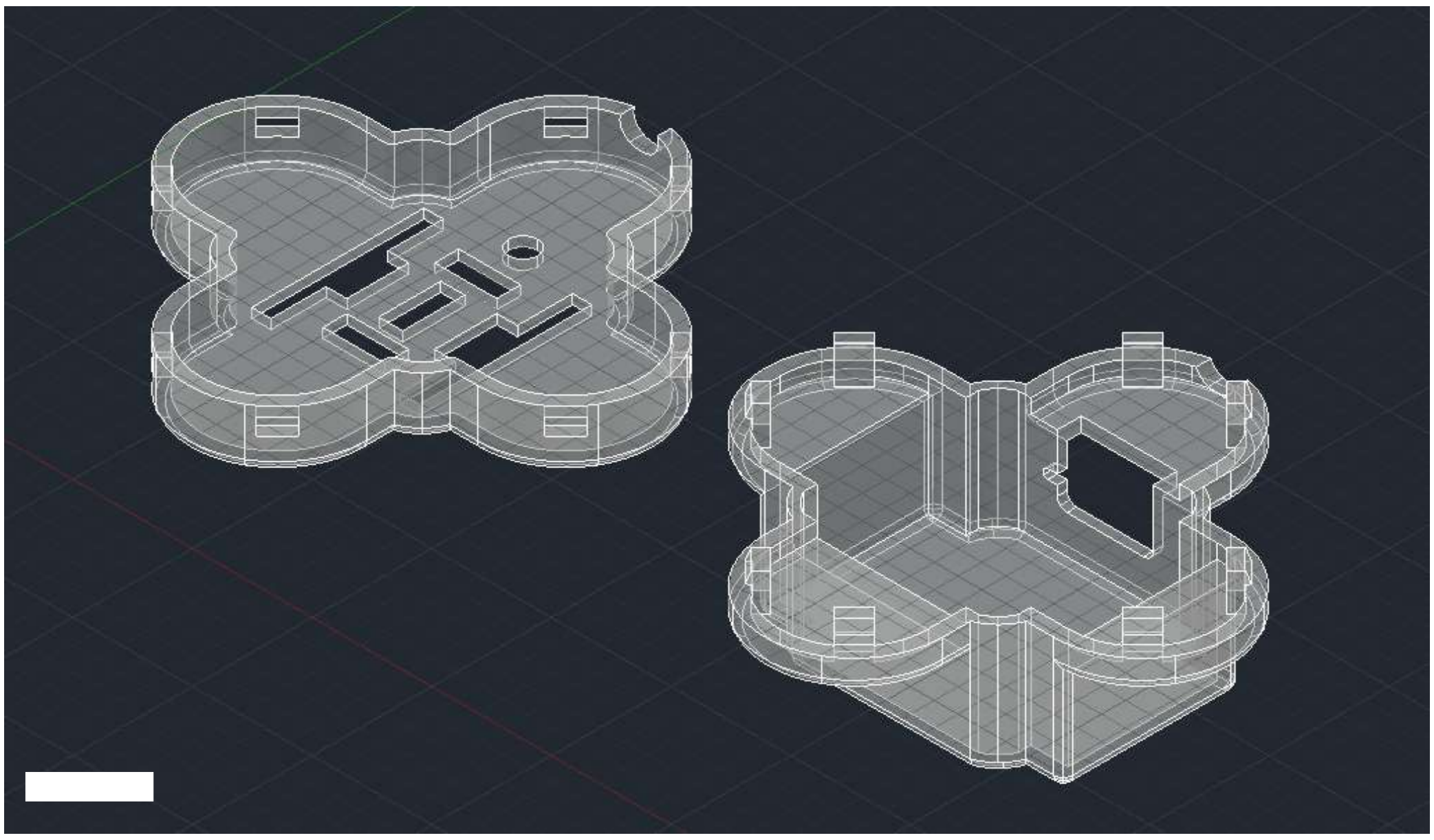

Figure S13. 3D CAD model of electronic enclosure. Scale bar = 1cm

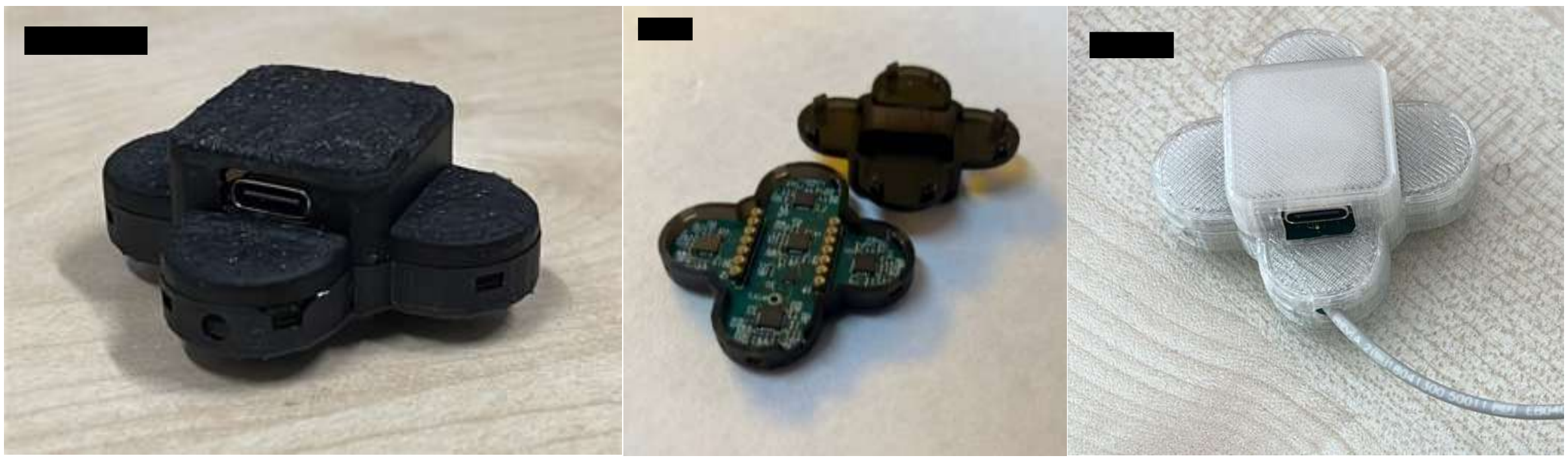

Figure S14. 3D-printed enclosures in resin (black) and PLA (translucent). Scale bar = 1cm

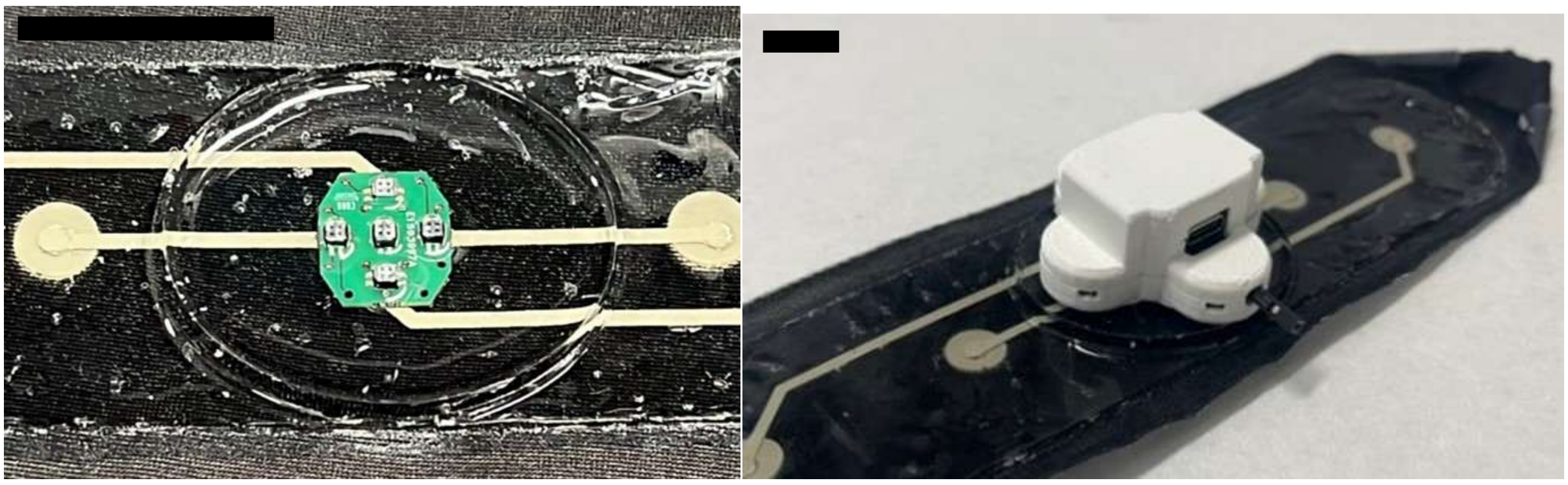

Figure S15. Left: Close-up view of interface PCB after PDMS encapsulation. Right: Final wireless electronic component after enclosure. Scale bar = 1cm

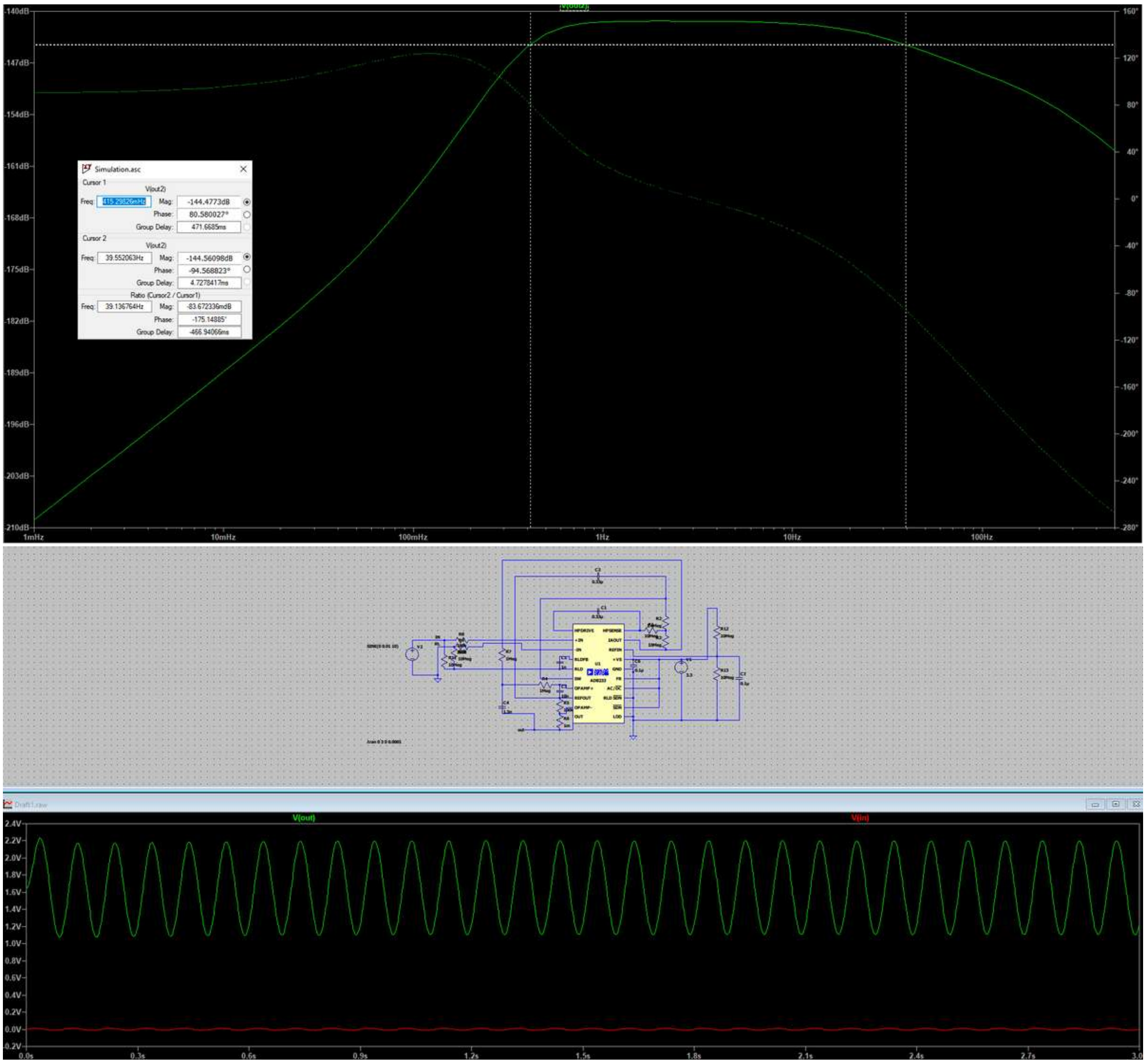

Figure S16. LTSpice simulations showing transient and frequency responses of analogue front-end circuitry (signal magnitude referred to 1V). Red trace input, green trace output

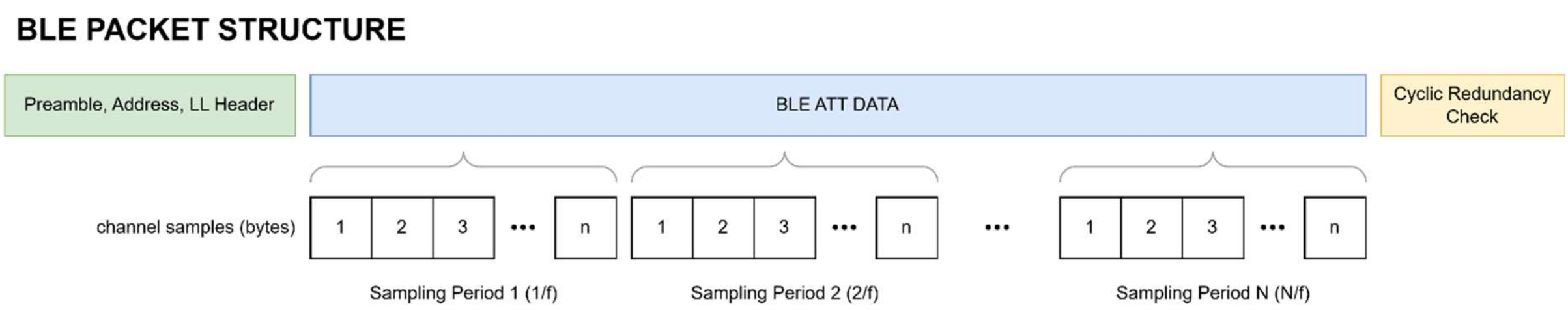

Figure S17. Structure of a BLE packet for the linear 5 electrode array. Characteristic data is carried in the BLE attribute data field

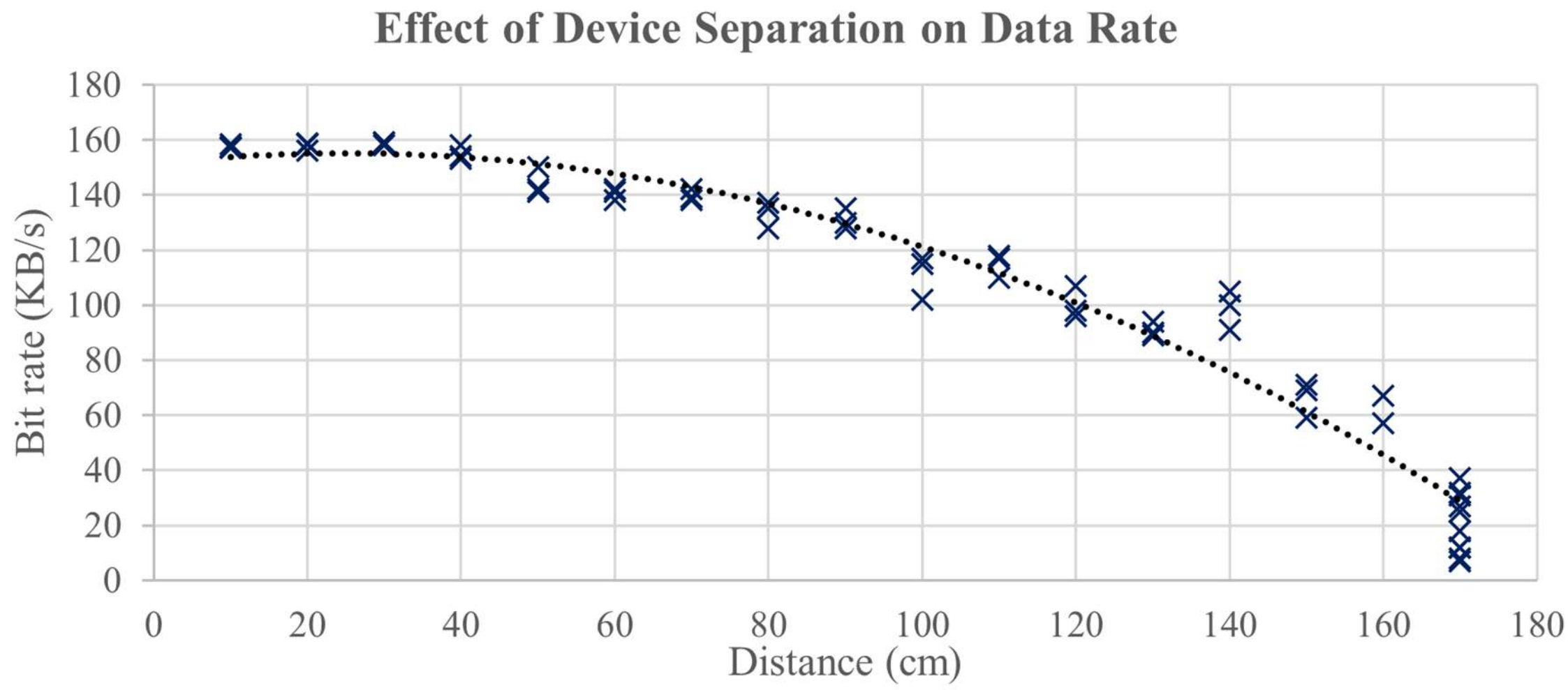

Figure S18. Effect of transmitter-receiver distance on data rate. At 1.7m the connection is lost

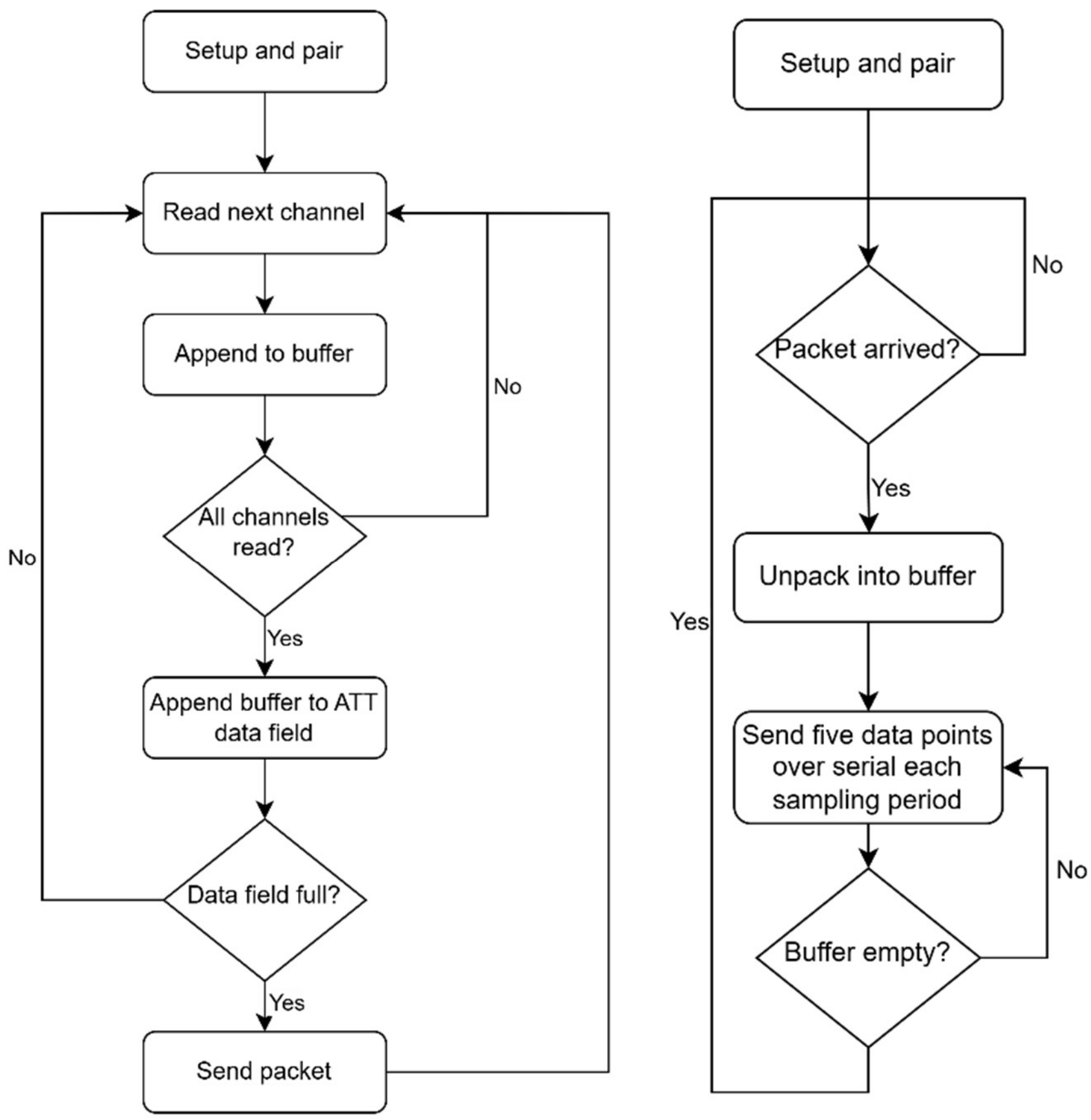

Figure S19. Flowcharts for server (left) and client (right) firmware

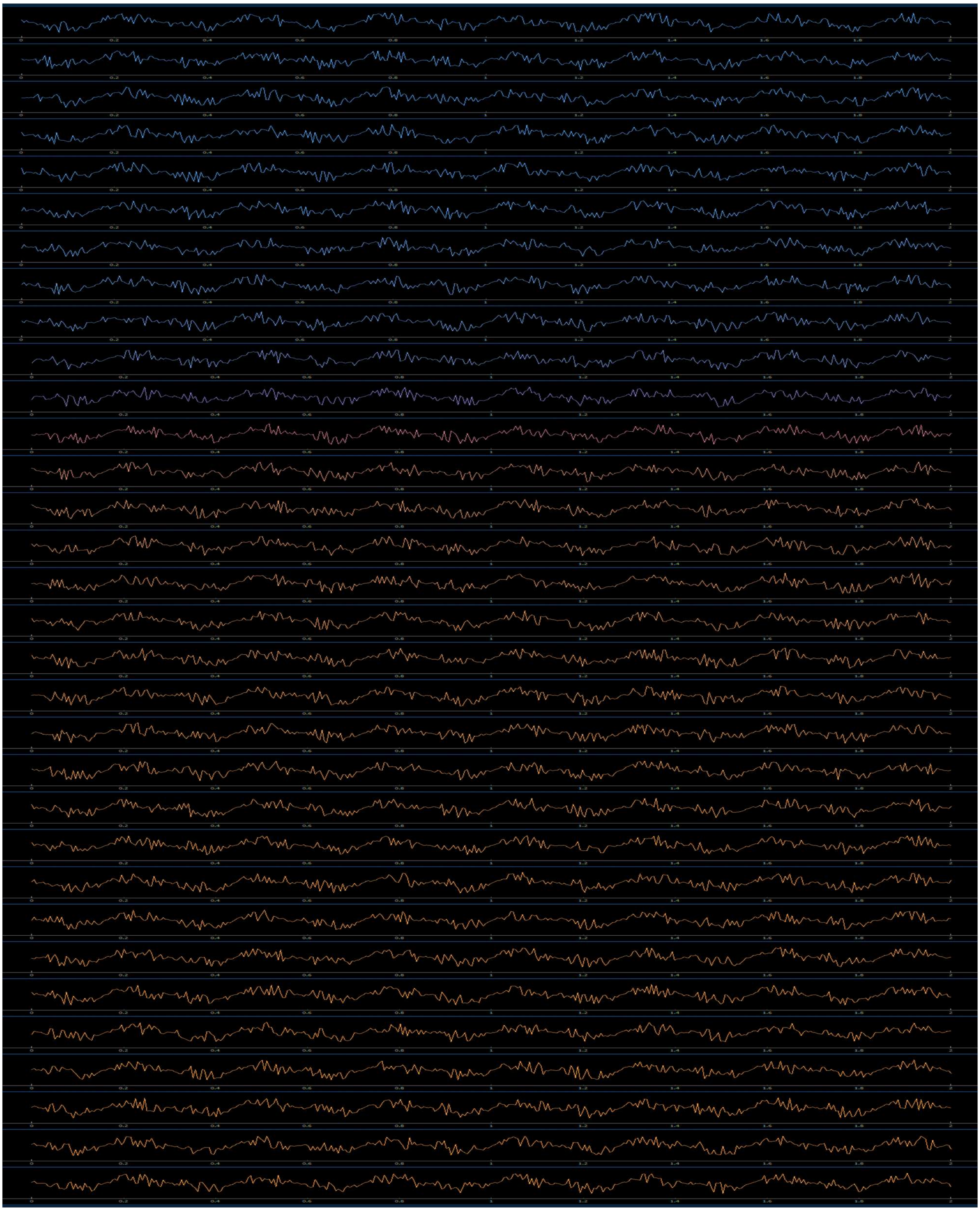

Figure S20. User interface when testing for channel throughput and data display speed. X-axis represents time, Y-axis represents voltage in uV

	A	B	C	D	E	F	G	H	I	J	K	L	M	N	O
1	Timestamp	Channel_1	Channel_2	Channel_3	Channel_4	Channel_5	Channel_6	Channel_7	Channel_8	Channel_9	Channel_1	Channel_1	Channel_1	Channel_1	Channel_1
2	13:48:21	99	173	133	163	155	157	155	155	182	122	185	142	181	147
3	13:48:21	148	161	148	123	97	116	153	116	123	152	98	145	90	145
4	13:48:21	126	161	149	122	119	141	132	154	119	138	152	135	160	122
5	13:48:21	151	118	129	132	148	119	136	123	133	119	156	156	105	140
6	13:48:21	124	134	127	128	127	133	139	127	133	124	119	126	139	119
7	13:48:21	127	130	128	124	129	126	123	124	129	124	131	123	129	126
8	13:48:21	126	125	125	125	126	128	128	124	129	130	126	130	127	124
9	13:48:21	132	128	137	136	118	122	127	124	130	134	132	128	124	117
10	13:48:21	134	116	125	114	116	138	121	107	125	152	131	125	101	127
11	13:48:21	140	147	107	137	119	114	111	120	114	124	140	106	157	144
12	13:48:21	110	134	147	138	123	79	131	118	98	102	94	163	77	148
13	13:48:21	71	99	159	106	79	133	137	135	83	127	102	103	125	85
14	13:48:21	120	163	161	127	99	64	96	86	127	135	95	97	169	86
15	13:48:21	64	64	127	84	64	100	135	98	159	110	121	121	121	121
16	13:48:21	86	122	56	97	94	124	53	51	125	135	97	107	157	125
17	13:48:21	134	127	46	46	105	149	90	90	98	83	120	75	83	90
18	13:48:21	119	80	87	41	158	49	97	52	86	52	99	96	54	130
19	13:48:22	87	99	86	127	94	100	101	119	151	119	86	51	38	88
20	13:48:22	94	84	94	85	35	68	133	68	85	119	88	43	88	134
21	13:48:22	40	89	80	51	80	85	101	51	74	85	94	40	127	77
22	13:48:22	73	127	128	34	77	124	84	51	127	124	85	85	81	93
23	13:48:22	43	66	114	98	114	71	85	84	93	121	63	108	74	113
24	13:48:22	86	135	41	73	123	52	84	70	102	135	108	127	102	100
25	13:48:22	135	99	97	135	112	135	96	130	95	96	130	121	56	96
26	13:48:22	60	135	120	97	135	46	61	98	61	54	83	53	46	98
27	13:48:22	97	78	59	119	59	50	97	92	55	51	51	45	58	83
28	13:48:22	59	102	122	81	102	95	121	129	56	64	58	121	56	85
29	13:48:22	54	119	62	127	99	104	62	85	66	88	88	125	91	120
30	13:48:22	95	90	122	57	90	57	61	103	90	58	93	90	87	103
31	13:48:22	78	102	78	95	94	91	94	127	65	103	103	67	79	127
32	13:48:22	119	121	103	103	104	86	99	99	78	91	86	78	91	79
33	13:48:22	110	87	113	105	102	101	111	105	89	119	127	113	111	111
34	13:48:22	107	103	116	109	103	106	110	108	115	106	127	112	114	109
35	13:48:22	120	124	119	123	120	124	122	127	119	123	124	122	126	121
36	13:48:22	128	131	130	132	129	131	130	130	127	131	135	133	129	130
37	13:48:22	136	145	137	135	142	141	140	130	128	137	128	139	132	138

	P	Q	R	S	T	U	V	W	X	Y	Z	AA	AB	AC
1	Channel_1	Channel_1	Channel_1	Channel_1	Channel_1	Channel_2	Channel_2	Channel_2	Channel_2	Channel_2	Channel_2	Channel_2	Channel_2	Channel_2
2	95	184	152	181	181	121	132	87	132	116	150	90	118	100
3	135	116	144	143	116	102	136	151	143	143	115	115	160	99
4	126	151	167	163	138	161	111	147	104	161	152	119	126	149
5	136	143	102	138	116	151	141	135	116	141	123	107	103	143
6	112	138	112	130	127	140	134	118	128	126	113	139	133	119
7	130	123	126	128	129	125	127	128	126	126	129	128	127	126
8	131	133	124	126	124	122	124	124	126	127	131	131	123	130
9	123	144	117	126	142	126	119	123	117	136	123	140	124	116
10	131	145	118	129	150	132	150	125	100	112	154	117	124	95
11	102	122	158	90	99	106	106	110	128	132	158	98	113	88
12	118	98	109	123	138	139	98	138	152	123	110	134	106	127
13	102	115	72	72	102	119	160	68	115	105	160	101	122	70
14	129	161	101	120	128	155	97	69	161	121	155	135	93	93
15	84	58	86	158	99	61	71	117	130	59	135	118	124	84
16	65	93	159	117	65	93	56	152	121	120	49	120	78	93
17	127	60	156	60	148	45	147	97	102	128	111	59	52	156
18	154	48	110	83	96	94	80	40	154	99	133	94	79	71
19	78	131	119	136	136	78	95	87	82	78	120	86	143	136
20	72	129	89	85	137	129	85	82	76	144	127	72	96	51
21	77	93	79	85	68	92	129	135	34	42	126	75	128	127
22	115	92	74	41	76	80	84	45	101	124	45	96	101	85
23	123	40	123	123	57	74	64	134	35	51	90	124	77	70
24	116	135	86	86	78	51	78	100	119	59	135	59	99	112
25	130	83	90	64	49	73	74	89	83	127	104	57	110	104
26	91	98	127	105	53	90	98	120	83	90	127	127	134	46
27	51	91	95	93	104	94	58	91	45	132	51	53	47	53
28	122	56	96	87	87	97	121	87	60	103	84	87	66	85
29	88	85	105	65	98	91	54	127	94	94	102	94	120	87
30	90	90	119	87	80	127	104	104	101	91	73	121	63	81
31	120	90	83	80	127	123	97	91	103	120	80	84	99	104
32	82	96	100	100	96	100	81	121	86	109	86	89	110	104
33	114	114	109	106	105	118	106	110	92	92	96	106	92	105
34	116	126	107	124	118	116	113	112	122	127	125	118	118	124
35	122	126	125	124	123	127	123	126	125	124	123	125	126	126
36	127	130	127	130	131	129	130	130	130	138	137	138	131	134
37	137	135	137	142	147	128	130	151	140	140	132	137	137	142

Figure S21. Example of data format in which the custom user interface stores recorded data. CSV format, first column represents timestamp and the remaining columns represents each channel in the given custom user order

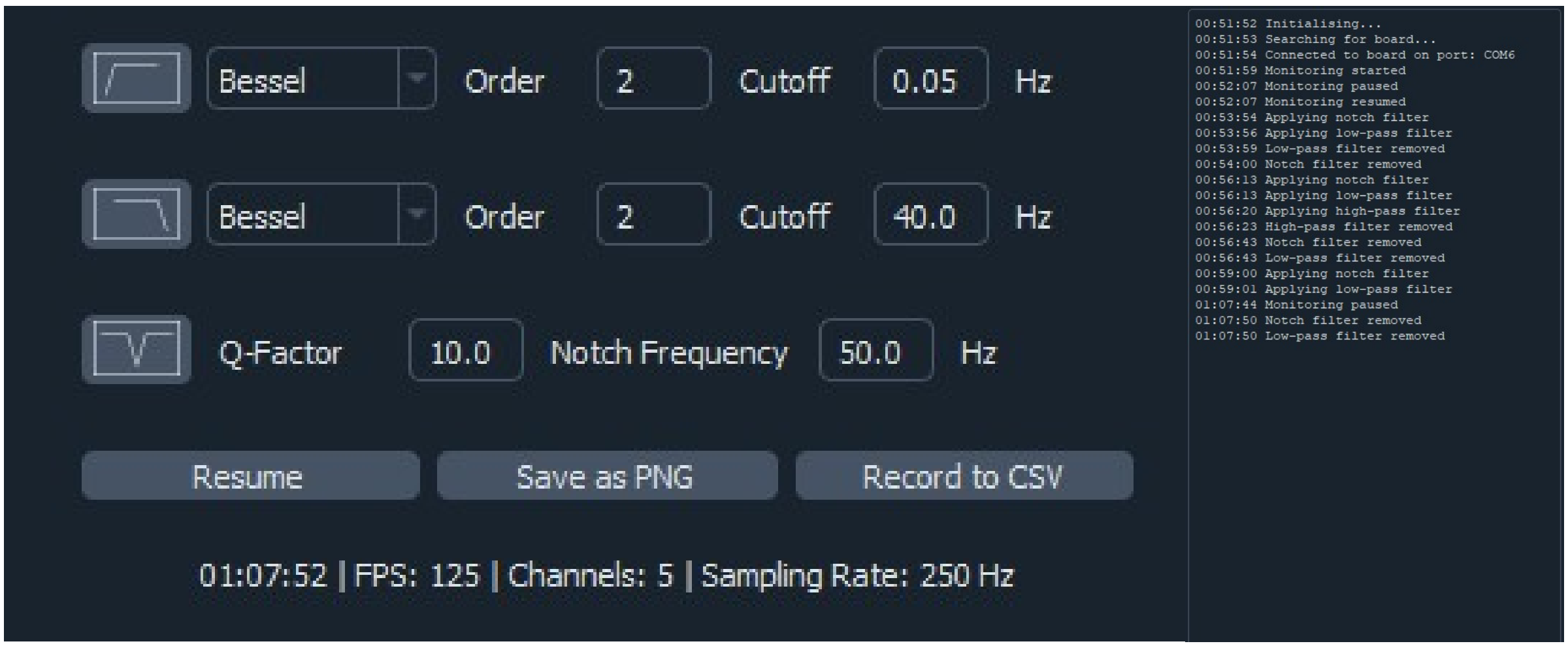

Figure S22. Custom control panel for applying digital filtering and saving data along with events control panel

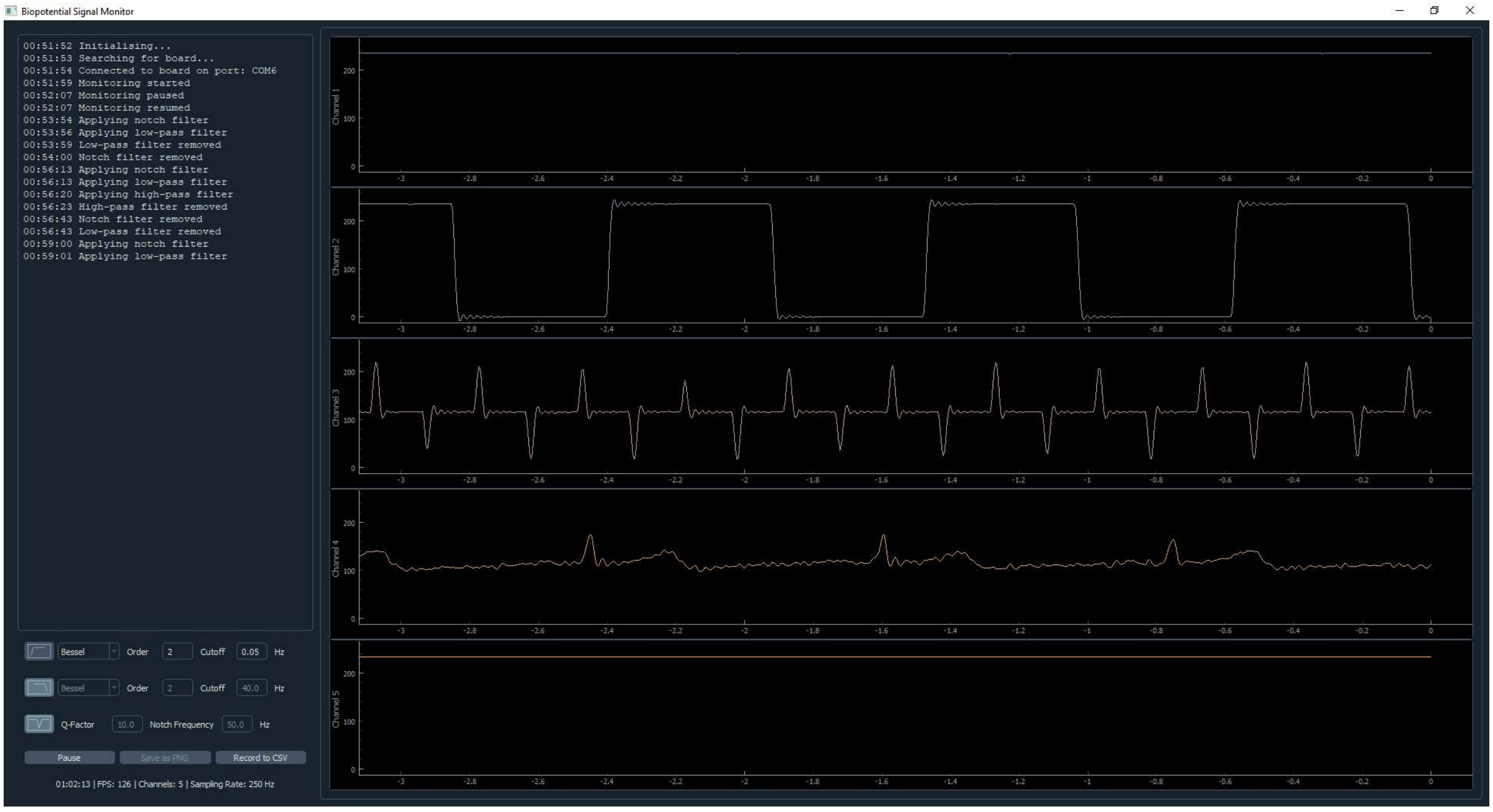

Figure S23. Overall view of custom-made biopotential signal monitor application and user interface

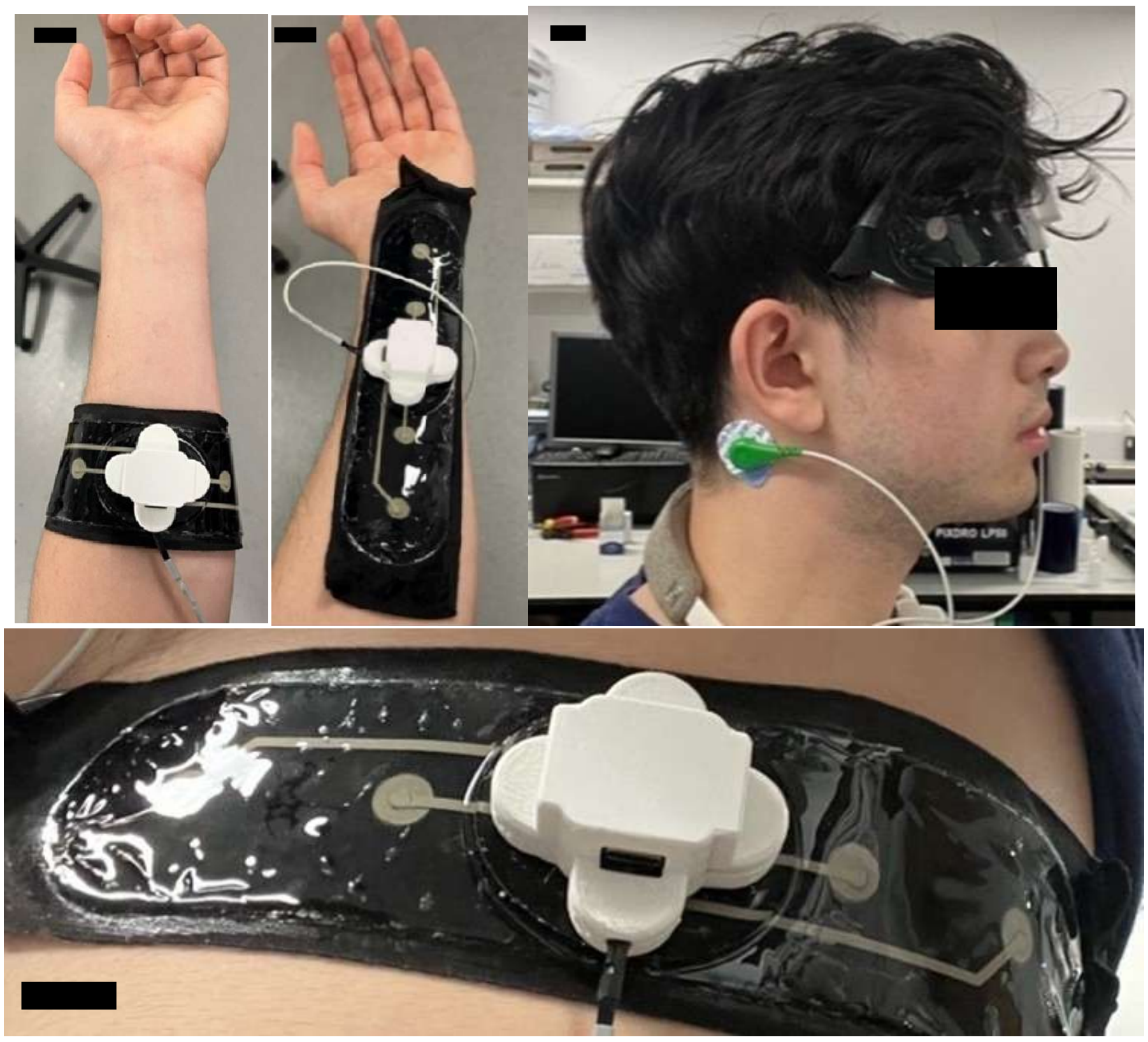

Figure S24. Recording setups for 5 channel electrode array. From top left to bottom right: EMG recordings, limb ECG recordings, EEG and EOG recordings and precordial ECG recordings. Scale bar = 1cm

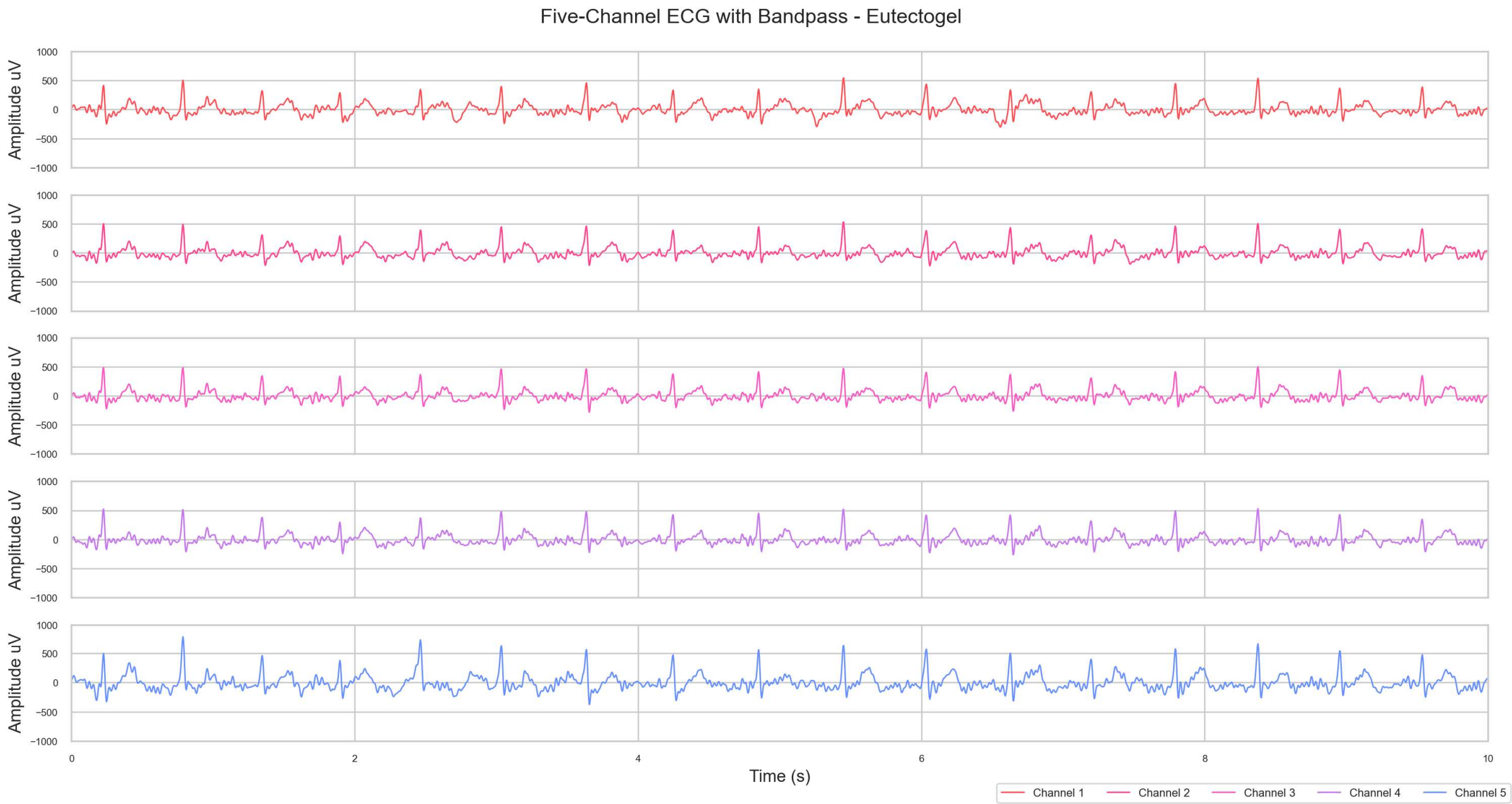

Figure S25. Simultaneous recording of ECG signals from linear 5 channel electrode array

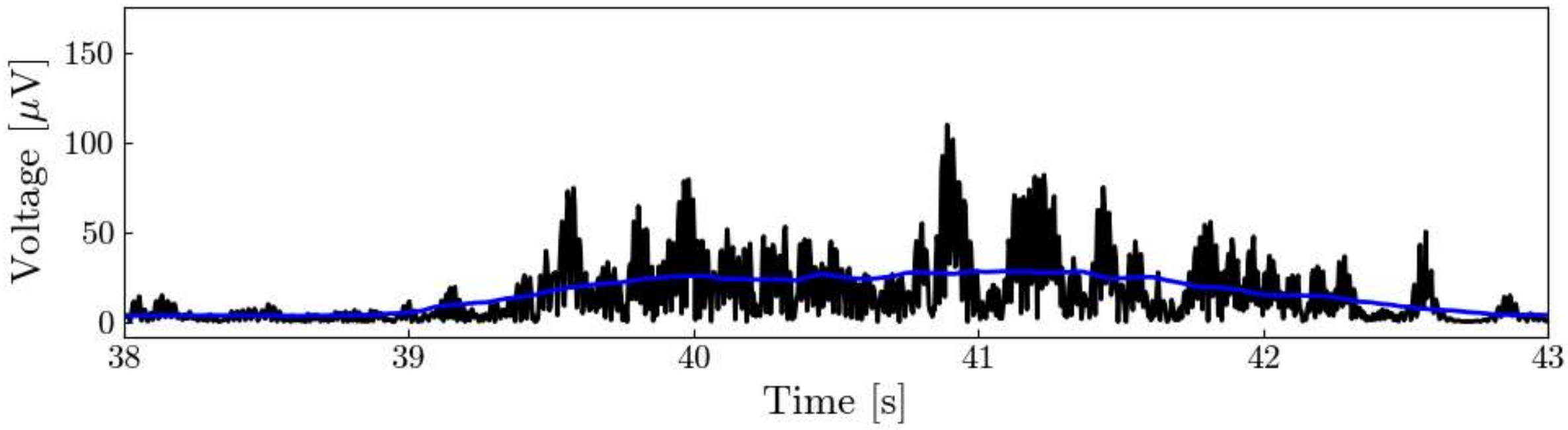

Figure S26. EMG envelope of 4-second sustained wrist flexion recorded with the ETG textile electrode array. Rectified EMG (black) and envelope (blue) illustrate muscle activation during the task

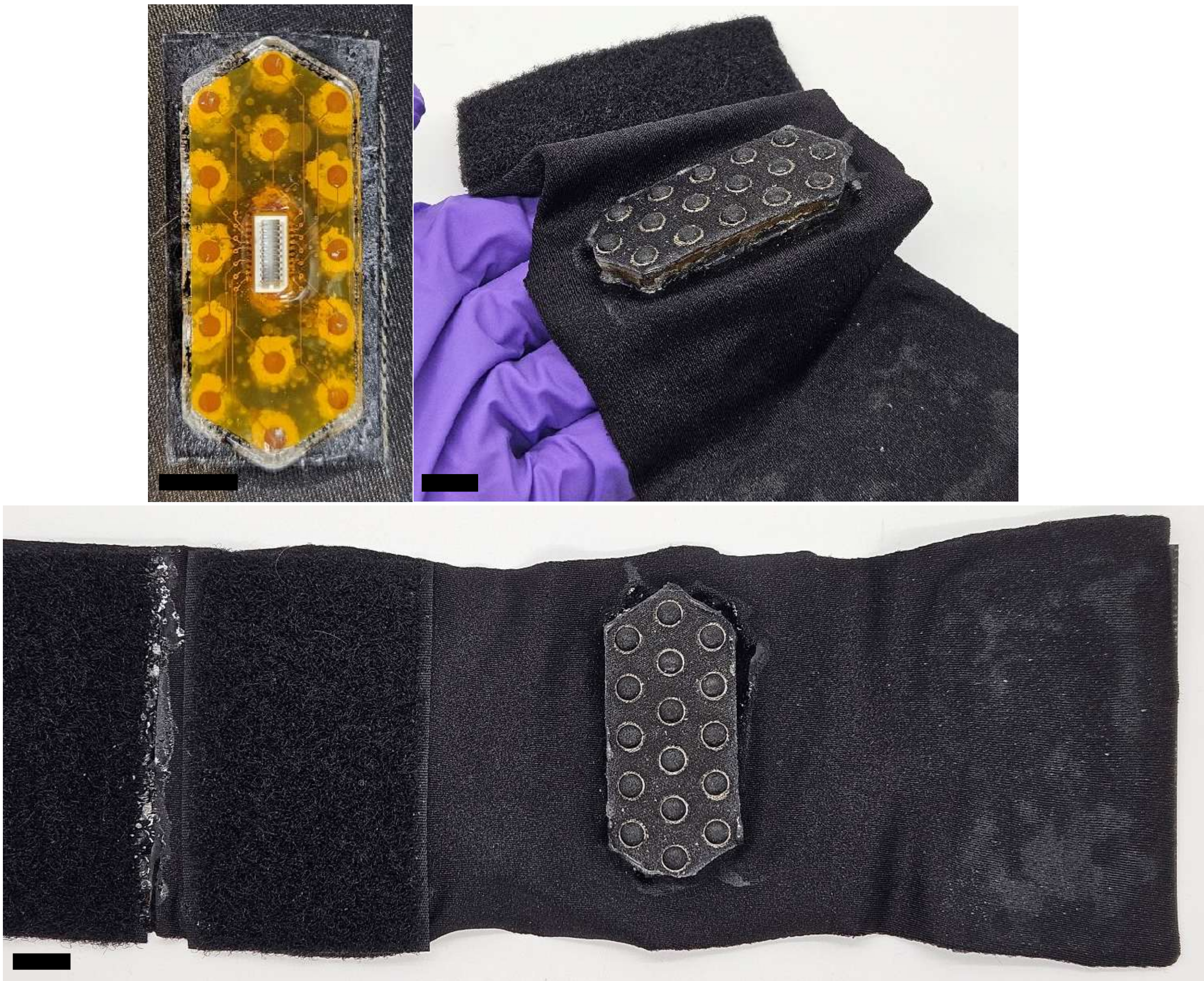

Figure S27. Structure and mounting of the 16-electrode EMG BSPM array. Top left: top view of the EMG array without the Velcro strap, showing electrode layout and inter-electrode spacing. Top right: bottom (skin-contact) view of the device attached to the Velcro strap. Bottom: top view of the skin-contact side of the EMG array. Scale bar = 1 cm

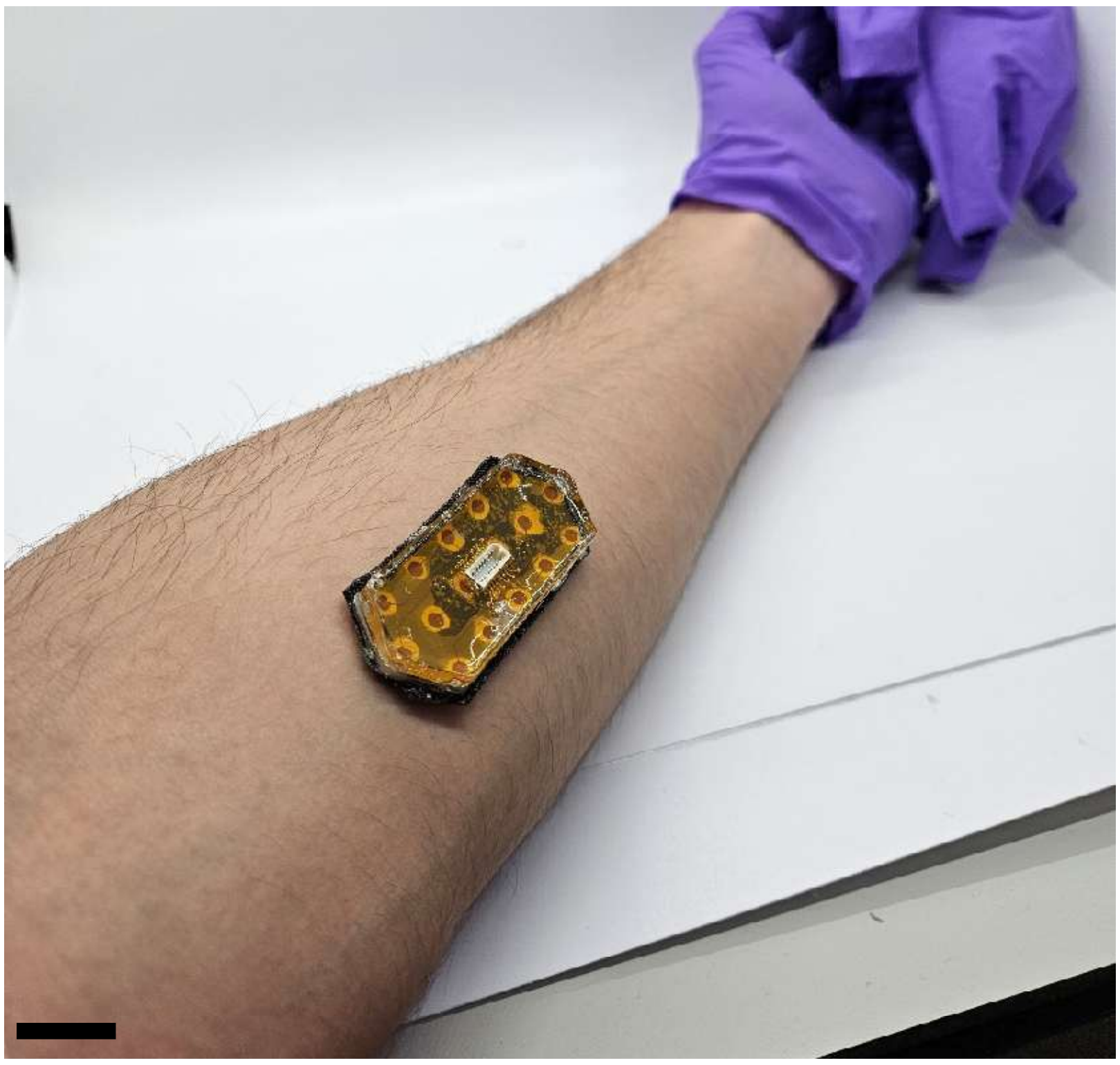

Figure S28. Relative size and placement of the 16-electrode EMG BSPM array on the forearm. The array is shown without the Velcro strap to illustrate electrode coverage and anatomical alignment with the underlying musculature. Scale bar = 2 cm

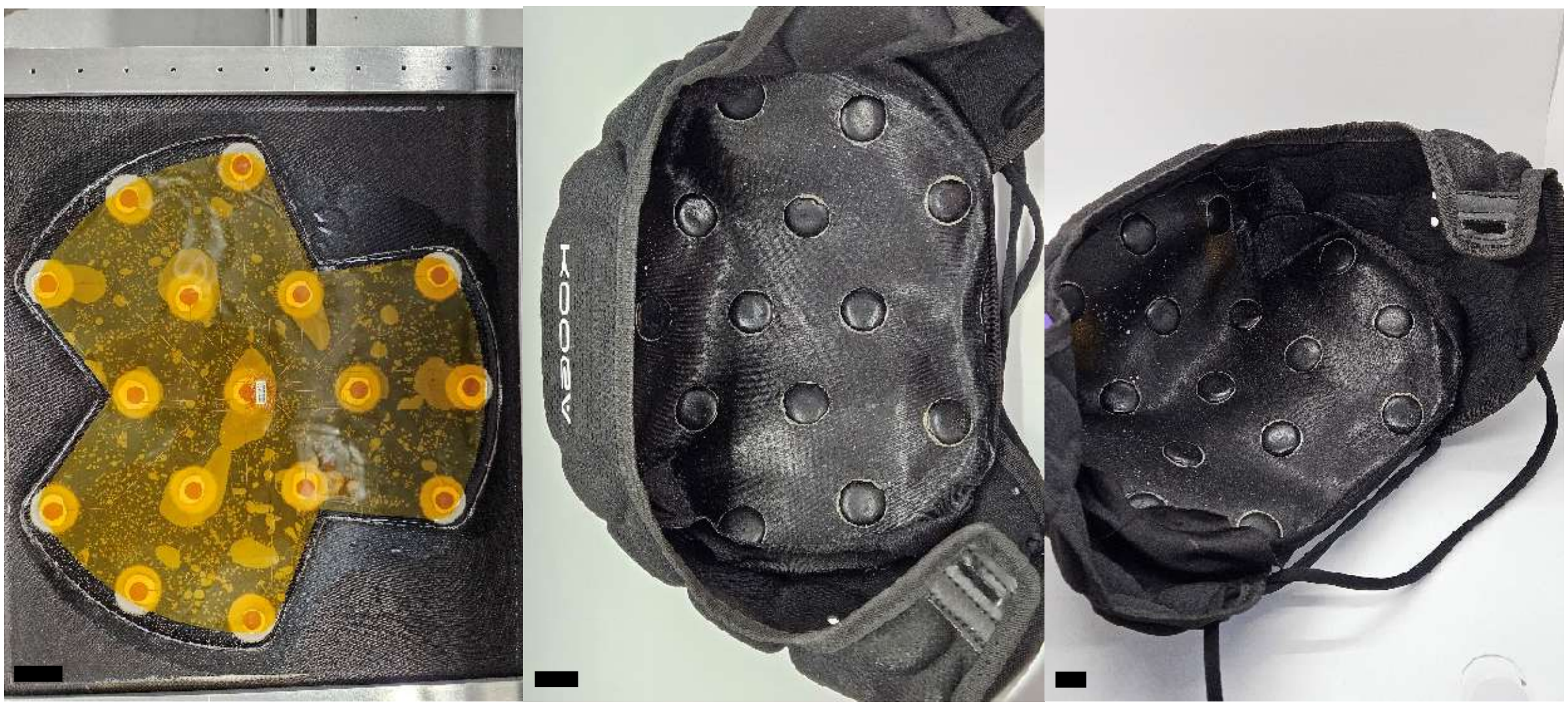

Figure S29. Structure and integration of the 16-electrode EEG BSPM array in a rugby headcap. Left: top view of the EEG array embedded within the headcap. Middle: bottom (skin-contact) view showing electrode distribution. Right: internal view of the array within the headcap, illustrating electrode placement and routing. Scale bar = 2 cm

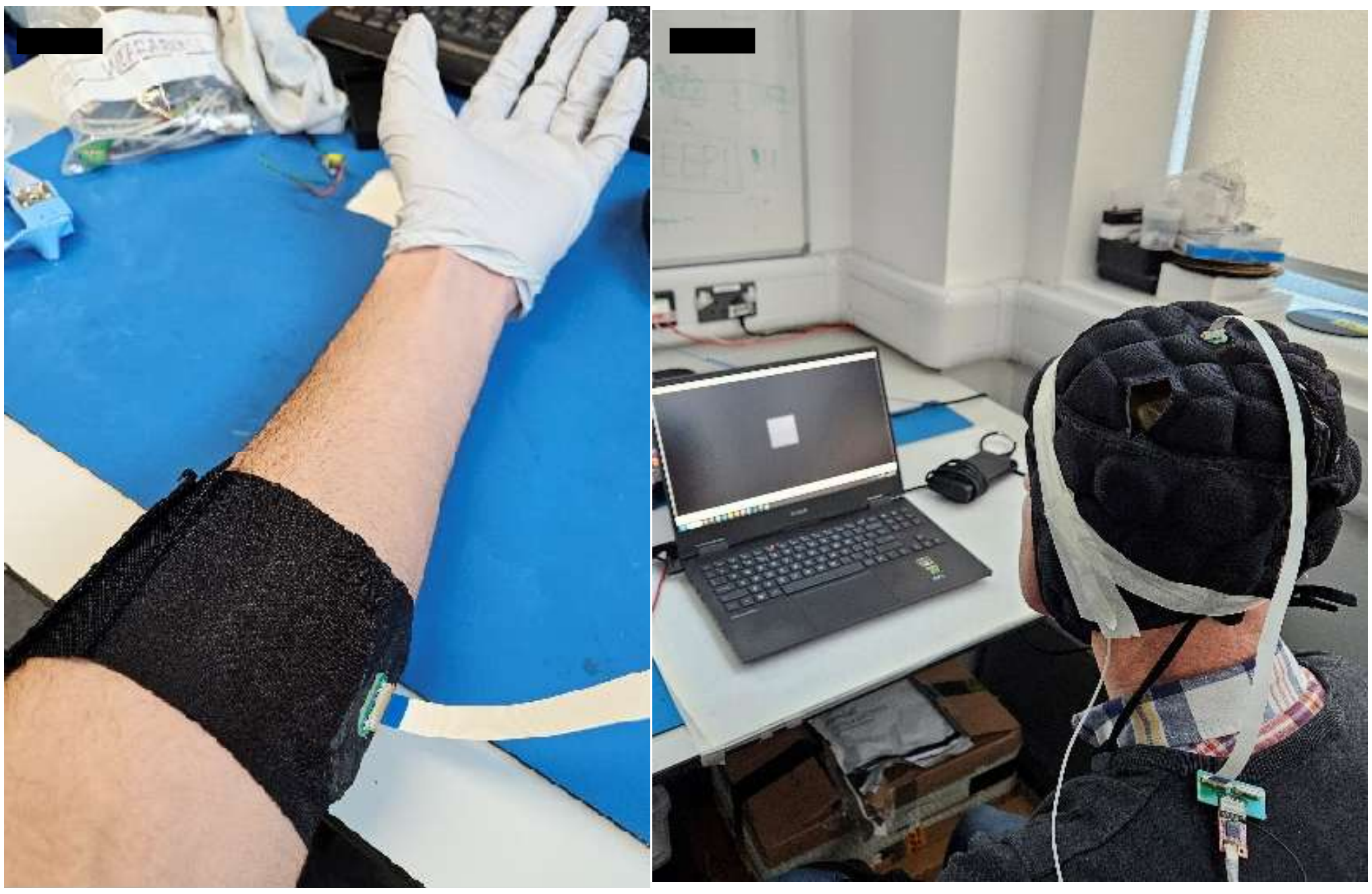

Figure S30. Recording setup for 16 channel electrode arrays. Left: muscular BSPM recordings, right: cerebral BSPM recordings. Scale bar = 3cm

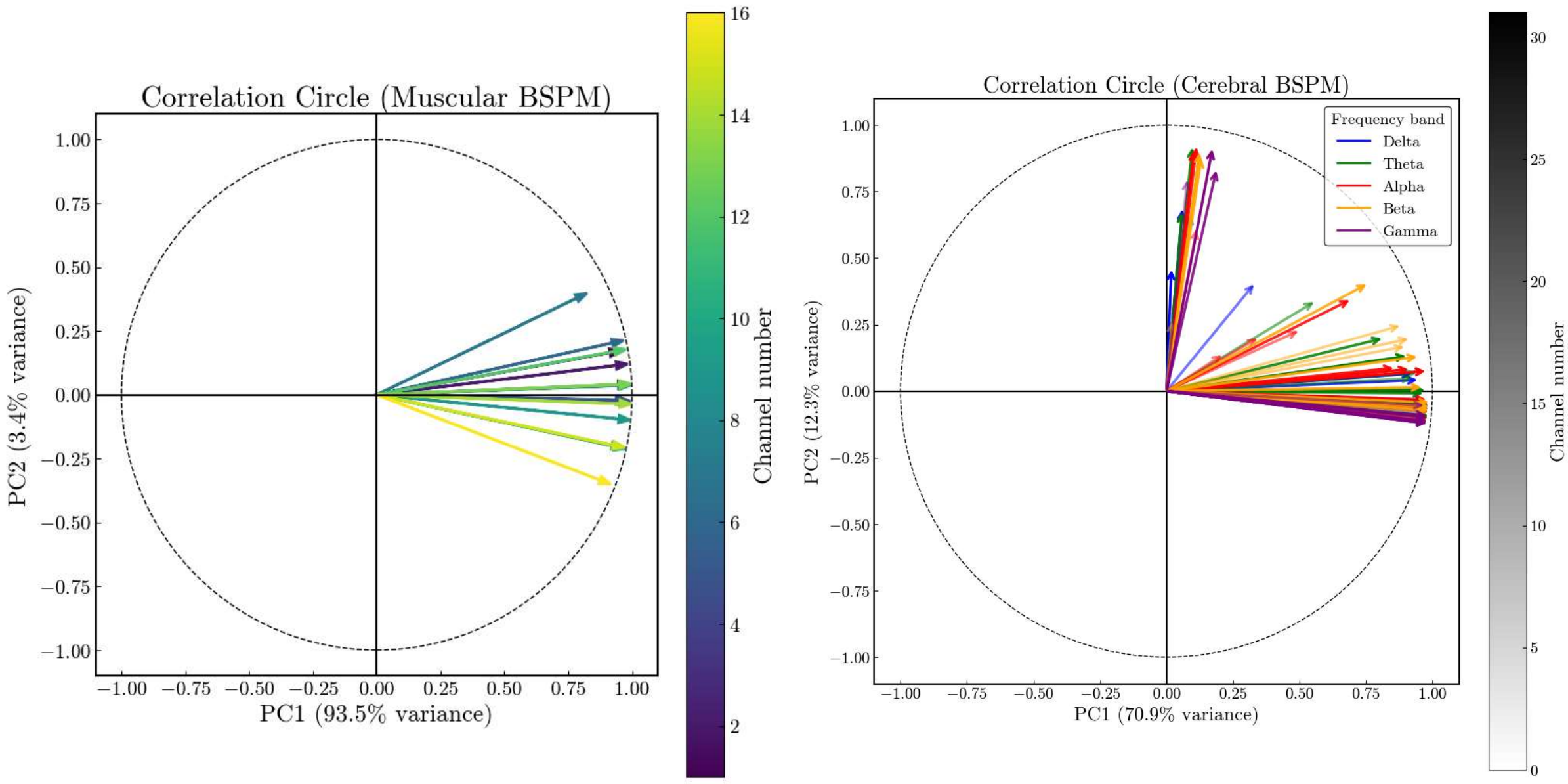

Figure S31. Correlation circles of muscular BSPM SNR features and cerebral BSPM EEG features. Left: Muscular SNR features. The correlation circle shows that the first principal component (PC1) is dominant, with all features exhibiting magnitudes close to 1 and clustered tightly together. The vectors form approximately a 60° opening relative to the x-axis, indicating that the features are highly correlated and contribute similarly to PC1. Right: Cerebral EEG features. The EEG correlation circle demonstrates that most features across all canonical frequency bands (Delta, Theta, Alpha, Beta, Gamma) have magnitudes near 1. The weaker features are randomly distributed across bands. There is a slightly greater contribution of PCA2 compared to the muscular features. Features from all bands are distributed across both PC1 and PC2, forming a relatively uniform spread. The vector cluster spans roughly 30°

along PC1 and 15° along PC2, highlighting a moderate correlation among features but with more nuanced variance than the muscular SNR data.

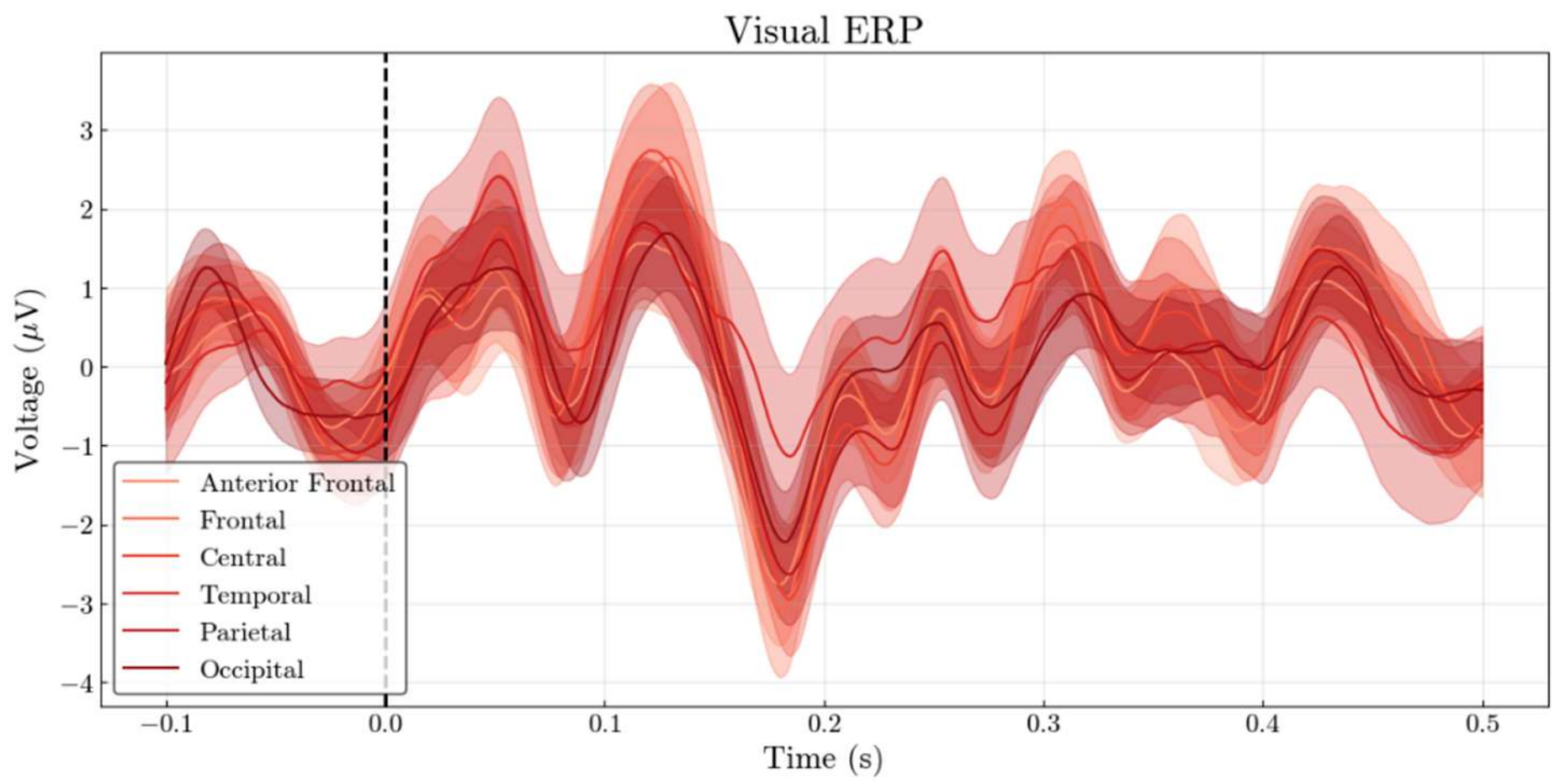

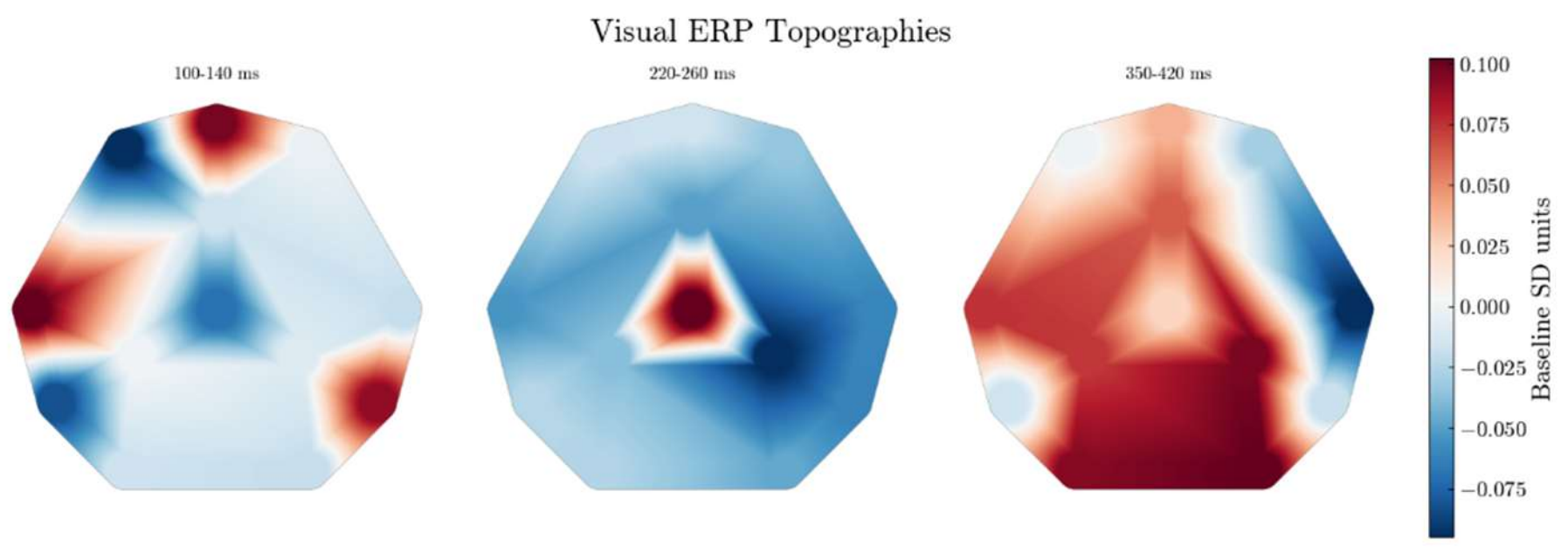

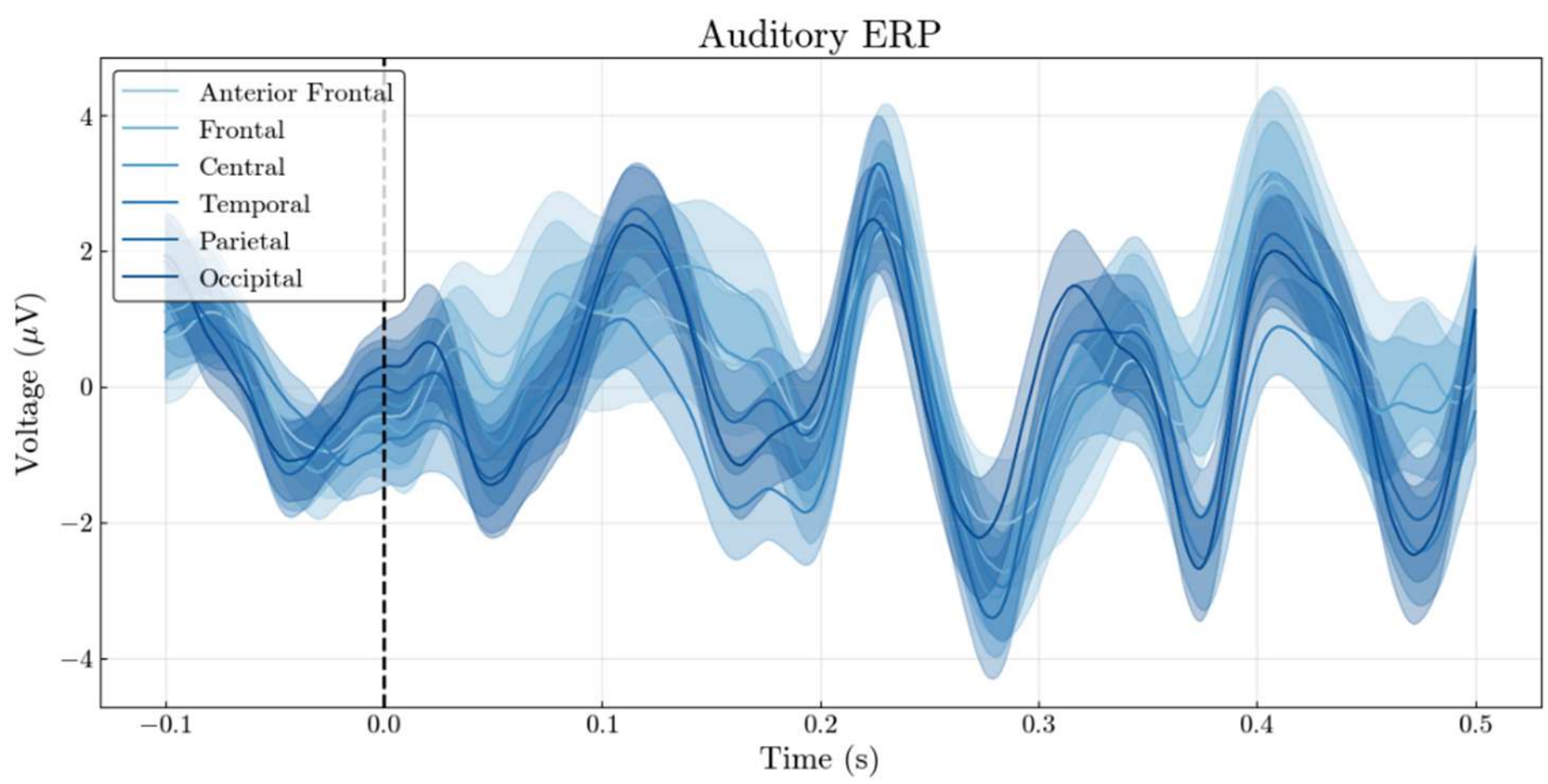

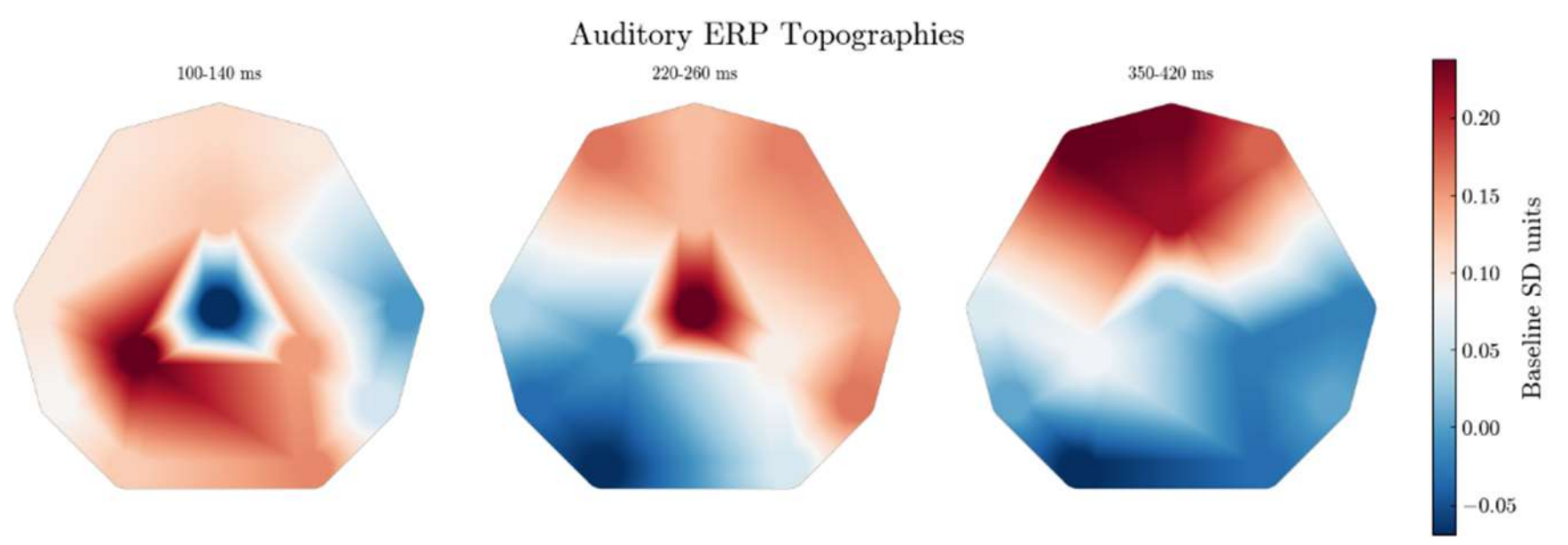

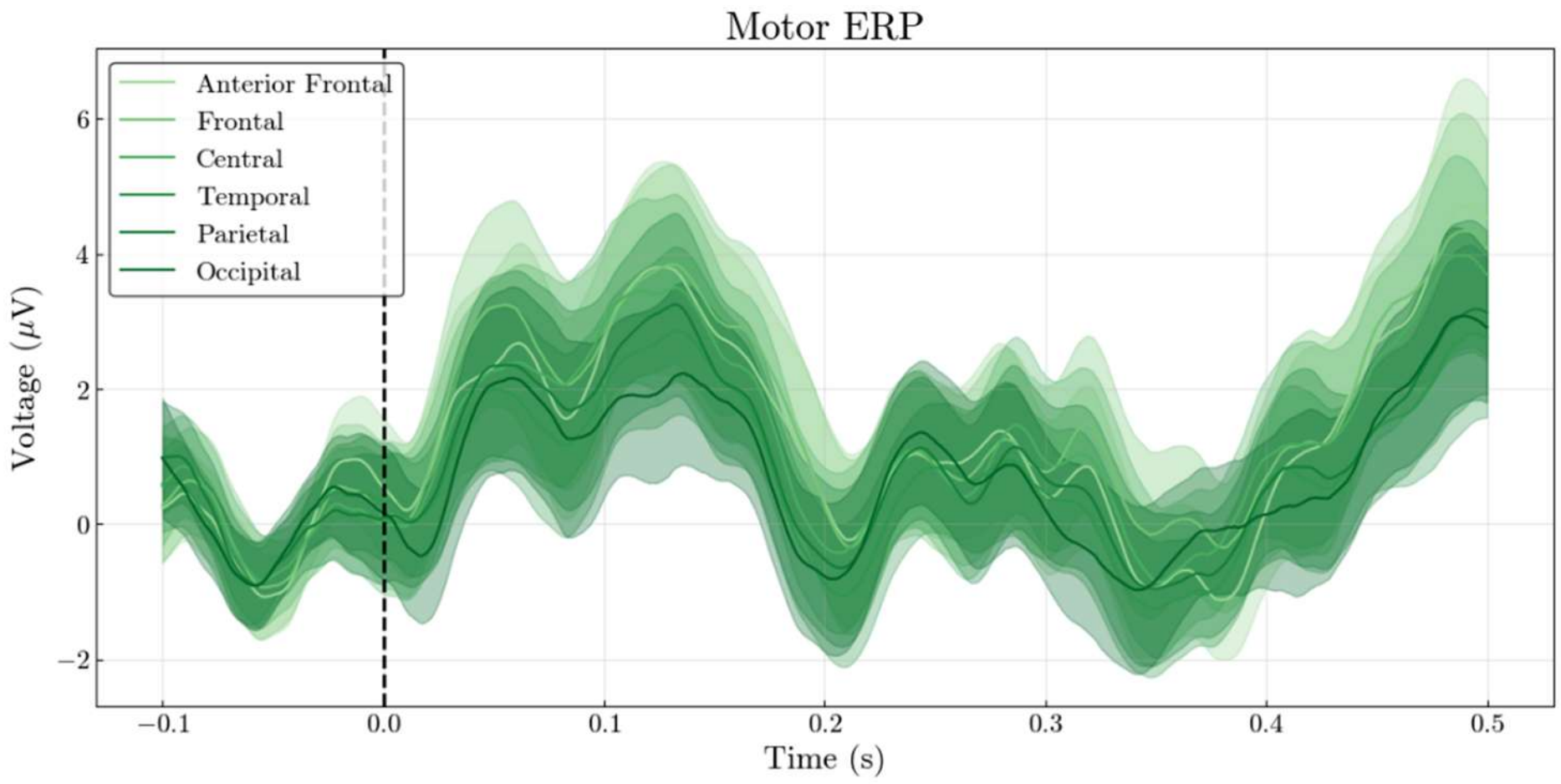

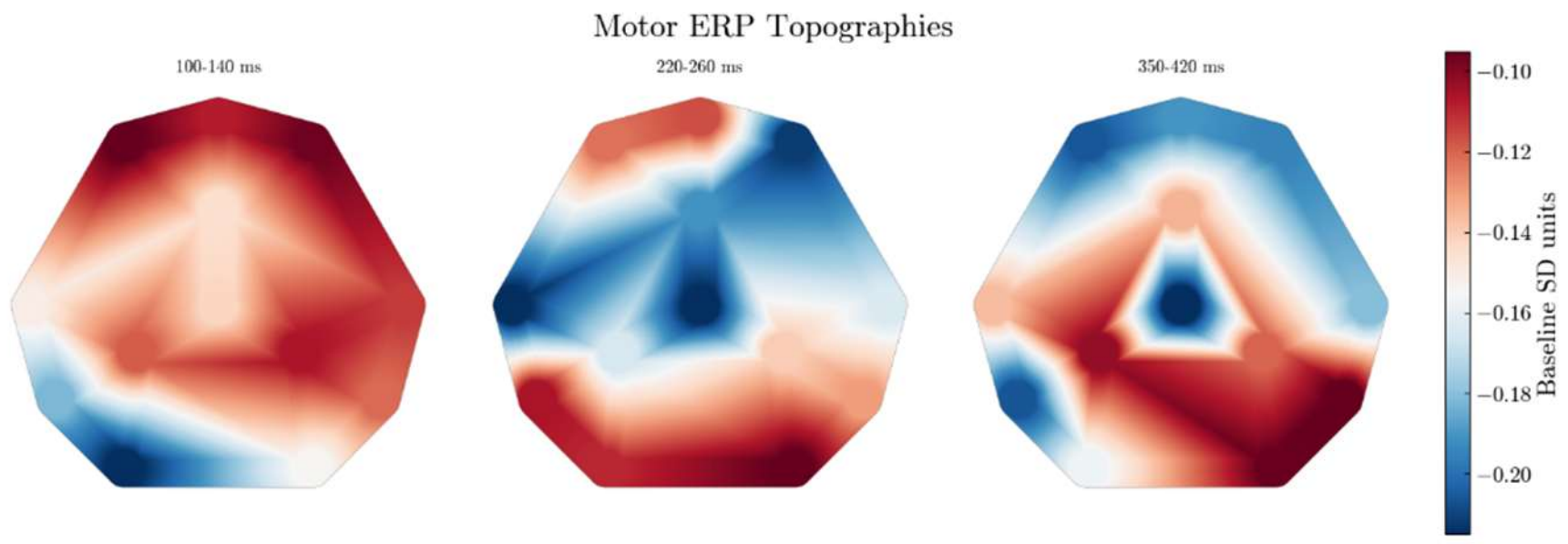

Figure S32. ERPs and topographies for visual, auditory, and motor stimuli. Mean ERP amplitudes were computed by averaging EEG signals across trials time-locked to stimulus onset (0 s) for anatomically defined cortical regions (anterior frontal, frontal, central, temporal, parietal, and occipital). ERP waveforms show the mean response per region in μV, with shaded areas indicating ±SEM. Topographical maps display ERP amplitudes normalised to baseline SD units across early, mid, and late post-stimulus windows (visual: 0.10–0.14 s, 0.22–0.26 s, 0.35–0.42 s; auditory and motor: same windows), highlighting spatial distribution and reliability of responses. Visual responses were initially distributed across electrodes, became focused on central electrodes at mid-latency, and predominantly engaged occipital regions at late latency. Auditory responses showed early temporal and frontal activation, mid-latency temporal with remaining frontal activity, and late frontal dominance. Motor responses were primarily frontal early, shifted to occipital with residual frontal mid-latency, and late activity engaged occipital, parietal, and temporal regions.

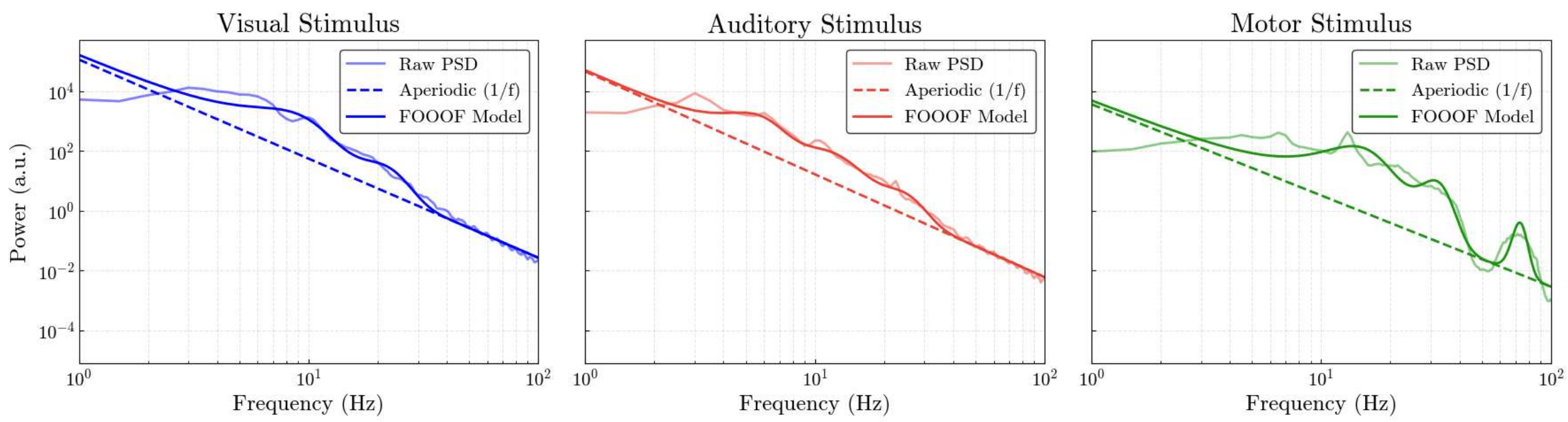

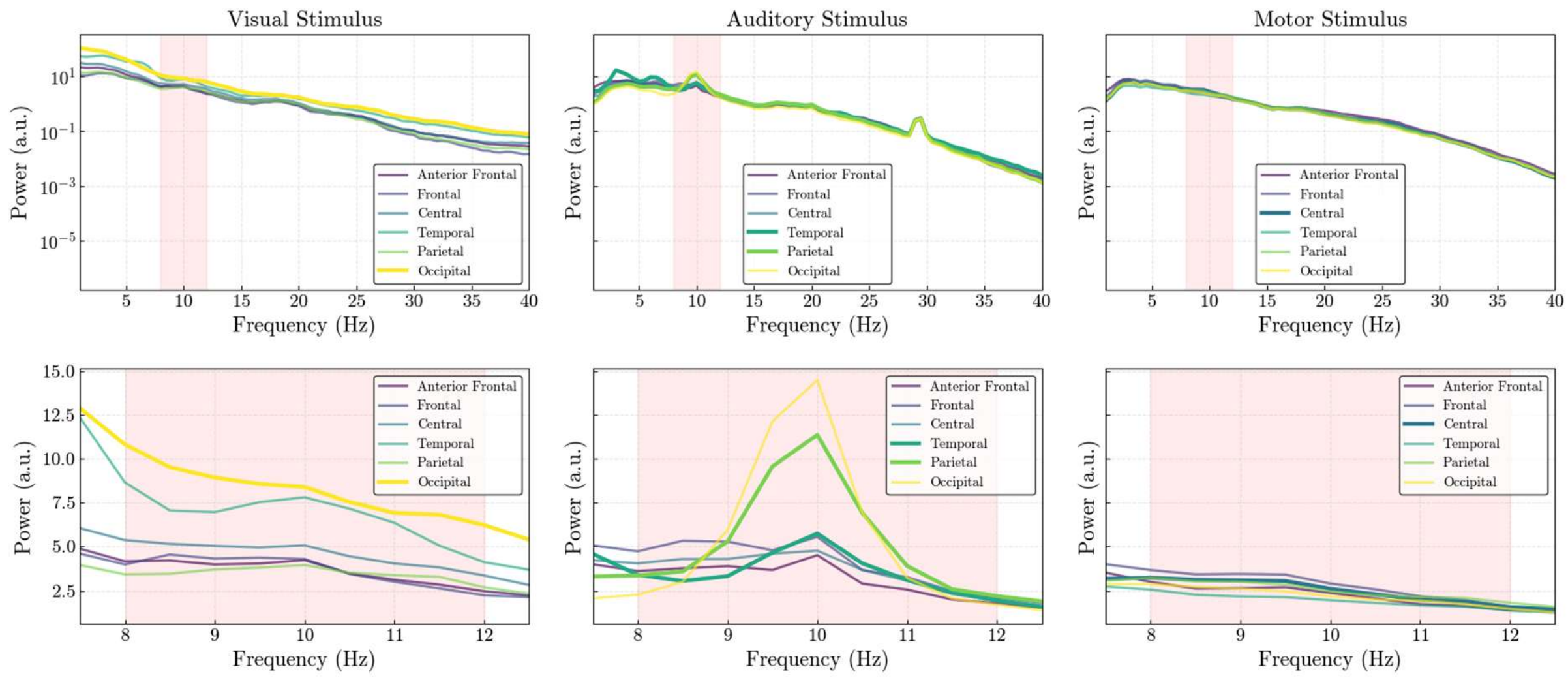

Figure S33. EEG spectral structure across frequency bands and regions, showing periodic and aperiodic components as well as alpha-band dynamics across tasks. EEG power spectral densities (PSDs) were computed from all channels and averaged across trials for visual, auditory, and motor stimulation conditions. The top row shows full-spectrum PSDs (1–100 Hz) on a logarithmic scale with FOOOF decomposition, where the solid line represents the observed spectrum and the dashed line the estimated aperiodic 1/f component. Across conditions, spectra show consistent broadband structure with prominent low-frequency activity and smaller periodic peaks in the alpha and beta ranges, while motor activity additionally shows relatively increased higher-frequency power. The bottom row shows region-specific PSDs for anterior frontal, frontal, central, temporal, parietal, and occipital regions, with full spectra displayed on a logarithmic scale (1–40 Hz) and a zoomed view of the alpha range (7.5–12.5 Hz) on a linear scale. Stimulus-relevant regions are subtly emphasised by increased line thickness. Alpha-band activity (8–12 Hz) shows clear spatial variability across conditions, with strongest posterior expression during visual stimulation, more distributed patterns during auditory stimulation, and relatively weaker and more anteriorly distributed expression during motor stimulation. This combined presentation highlights both aperiodic and oscillatory structure in the EEG and their spatial heterogeneity across regions and tasks.

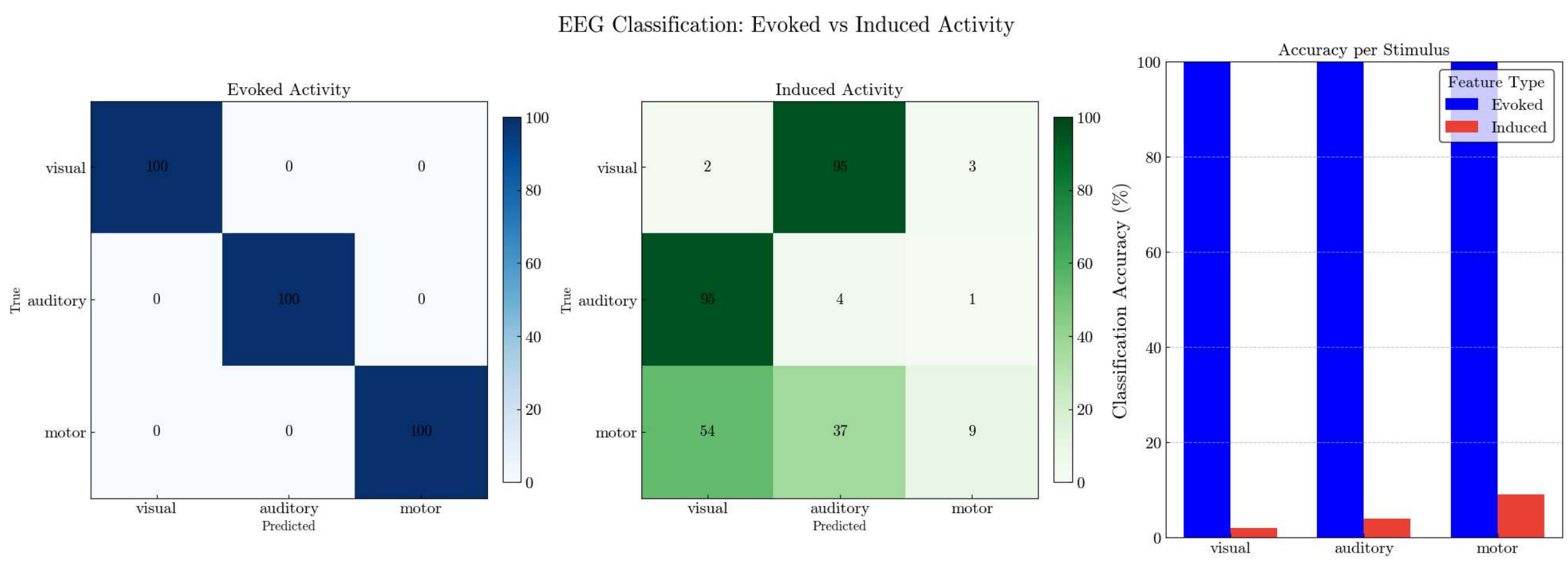

Figure S34. EEG Classification: Evoked vs. Induced Activity. Left and middle panels show confusion matrices for classification based on evoked and induced EEG features, respectively, with rows representing true stimulus labels and columns representing predicted labels. Right panel shows classification accuracy for each stimulus type, comparing evoked (blue) and induced (orange) features. Evoked activity provided near-perfect classification across all stimuli (100% overall), indicating strong time-locked responses to visual, auditory, and motor events. In contrast, induced features, representing non-phase-locked oscillatory activity, yielded low classification performance, ranging between ~5–10% depending on stimulus, with motor-related activity achieving the highest accuracy.

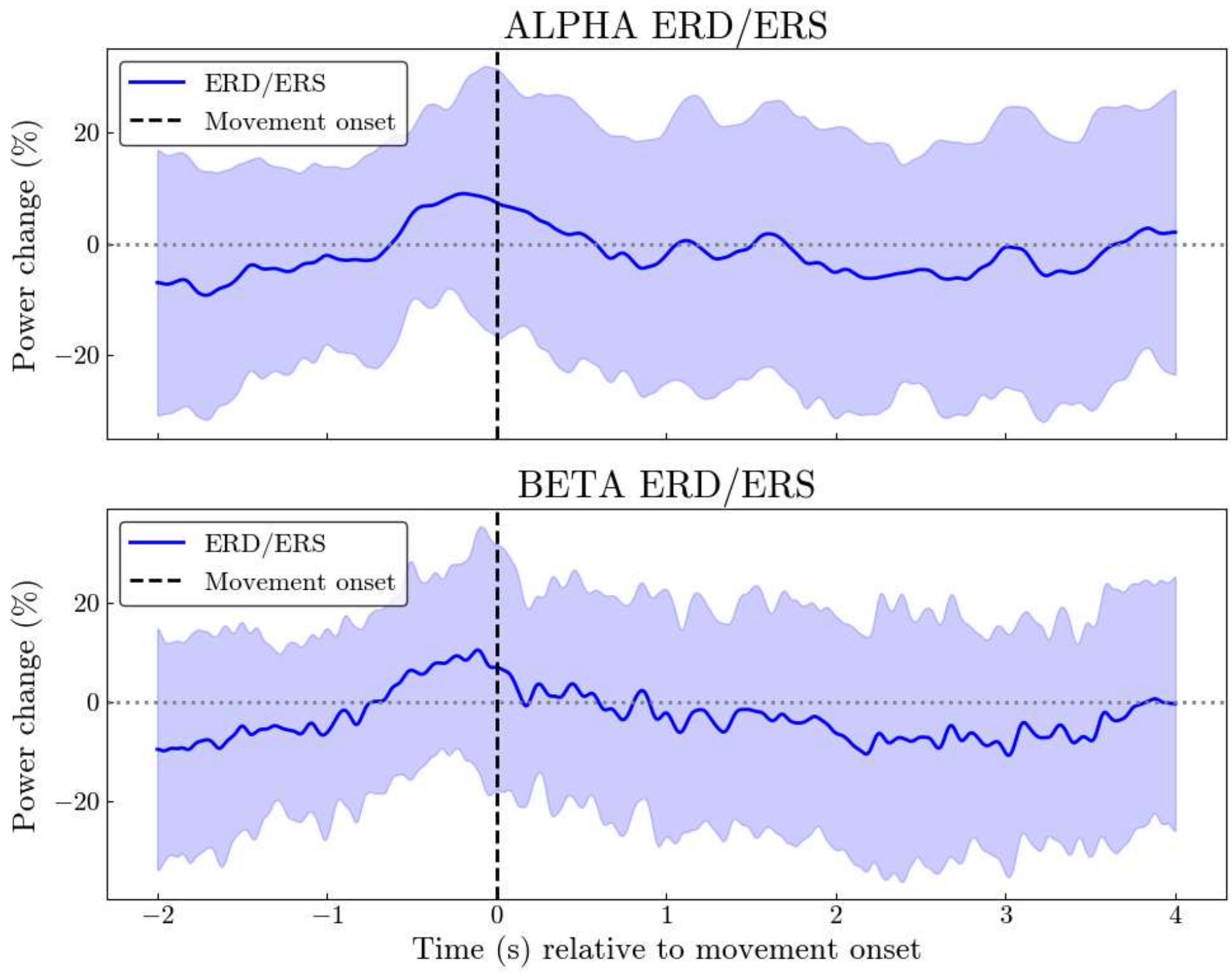

Figure S35. Movement-related ERD/ERS in motor cortex. EEG signals from motor-related channels were bandpass filtered in the alpha (8–12 Hz) and beta (13–30 Hz) bands, Hilbert-transformed, and normalised to a pre-movement baseline (-1.5 to -0.5 s). Shaded regions indicate ±1 standard deviation across trials. The dashed vertical line denotes the approximate movement onset. Both frequency bands exhibit a negative deflection ~2 s before movement, a pronounced positive peak at movement onset (reflecting synchronization with motor execution), and a subsequent negative peak ~2–3 s post-onset returning to baseline. These patterns reflect movement-related cortical oscillatory dynamics, illustrating anticipatory, execution, and post-movement phases.

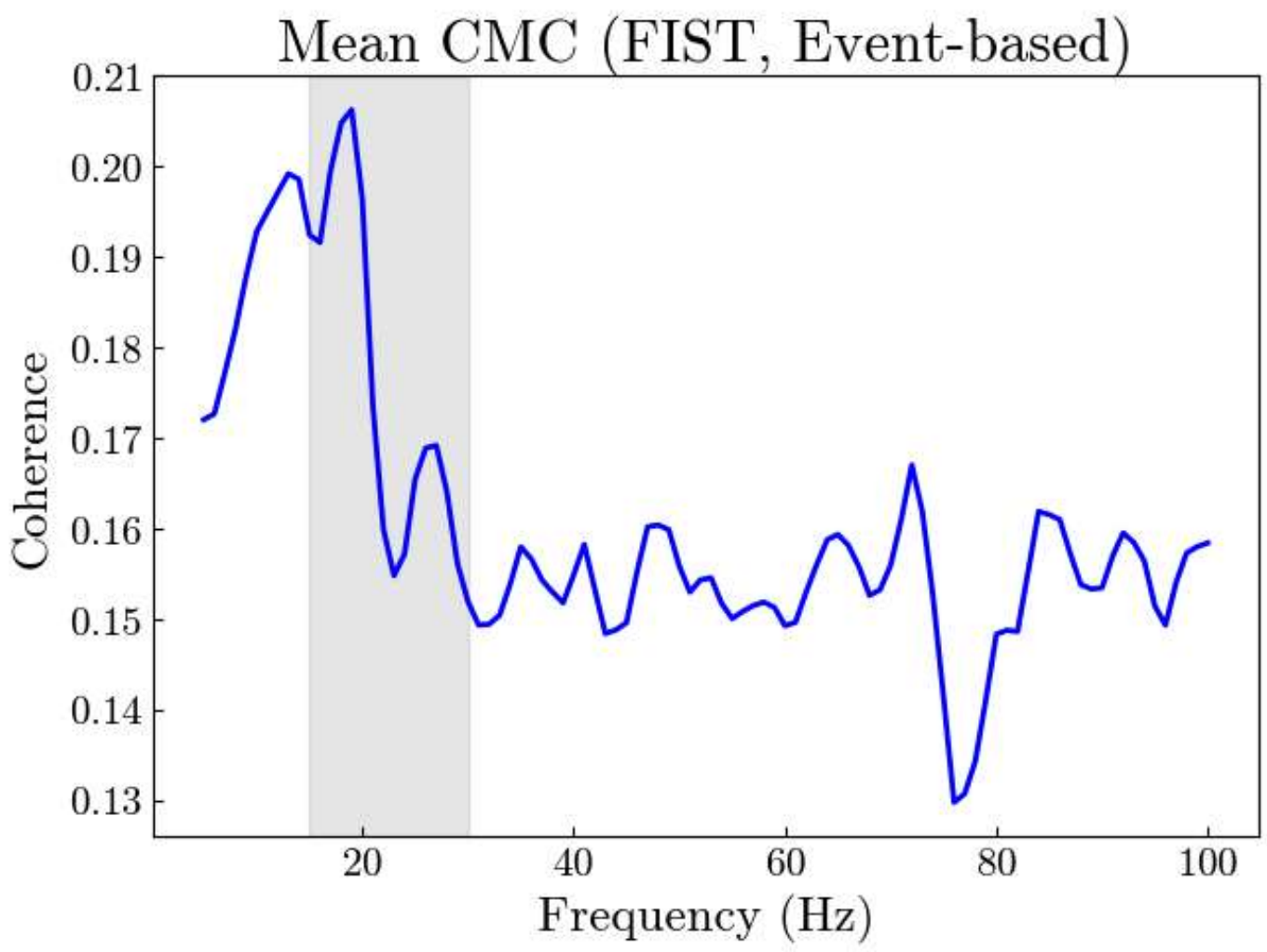

Figure S36. Event-based cortico-muscular coherence (CMC) during hand flexion movement. Mean CMC was computed between EEG and EMG signals across all segments corresponding to hand flexion events. The grey shaded region marks the beta frequency band (15–30 Hz), where cortico-muscular coupling is strongest. Low-frequency components (<8 Hz) were removed to avoid contamination by slow drifts or movement artifacts. This figure demonstrates that the electrode array captures physiologically relevant EEG–EMG coherence during voluntary hand movements.

References

[1] Coosemans, J.; Hermans, B.; Puers, R. , Integrating wireless ECG monitoring in textiles, *Sensors and Actuators. A. Physical.* 2006, *130*, 48–53, https://dx.doi.org/10.1016/j.sna.2005.10.052.

[2] Puurtinen, M. M.; Komulainen, S. M.; Kauppinen, P. K.; Malmivuo, J. A. V.; Hyttinen, J. A. K. , Measurement of noise and impedance of dry and wet textile electrodes, and textile electrodes with hydrogel, *2006 International Conference of the IEEE Engineering in Medicine and Biology Society.* 2006, *2006*, 6012–6015, https://doi.org/10.1109/IEMBS.2006.260155.

[3] Finni, T.; Hu, M.; Kettunen, P.; Vilavuo, T.; Cheng, S. , Measurement of EMG activity with textile electrodes embedded into clothing, *Physiological Measurement.* 2007, *28*, 1405–1419, https://doi.org/10.1088/0967-3334/28/11/007.

[4] Zheng, J. W.; Zhang, Z. B.; Wu, T. H.; Zhang, Y. , A wearable mobihealth care system supporting real-time diagnosis and alarm, *Medical & Biological Engineering & Computing.* 2007, *45*, 877–885, https://doi.org/10.1007/s11517-007-0221-y.

[5] Bouwstra, S.; Feijs, L.; Wei Chen; Oetomo, S. B. , Smart Jacket Design for Neonatal Monitoring with Wearable Sensors, *2009 Sixth International Workshop on Wearable and Implantable Body Sensor Networks.* 2009, 162–167, https://doi.org/10.1109/BSN.2009.40.

[6] Silva, M.; Catarino, A.; Carvalho, H.; Rocha, A.; Monteiro, J.; Montagna, G. , Study of vital sign monitoring with textile sensors in swimming pool environment, *2009 35th Annual Conference of IEEE Industrial Electronics.* 2009, 4426–4431, https://doi.org/10.1109/IECON.2009.5414898.

[7] Farina, D.; Lorrain, T.; Negro, F.; Jiang, N. , High-density EMG E-Textile systems for the control of active prostheses, *2010 Annual International Conference of the IEEE Engineering in Medicine and Biology.* 2010*, 2010*, 3591–3593, https://doi.org/10.1109/iembs.2010.5627455.

[8] Hoi-Jun Yoo; Yoo, J.; Long Yan , Wireless fabric patch sensors for wearable healthcare, *2010 Annual International Conference of the IEEE Engineering in Medicine and Biology.* 2010*, 2010*, 5254–5257, https://doi.org/10.1109/iembs.2010.5626295.

[9] Marozas, V.; Petrenas, A.; Daukantas, S.; Lukosevicius, A. , A comparison of conductive textile-based and silver/silver chloride gel electrodes in exercise electrocardiogram recordings, *Journal of Electrocardiology.* 2011*, 44*, 189–194, https://doi.org/10.1016/j.jelectrocard.2010.12.004.

[10] Cho, G.; Jeong, K.; Paik, M. J.; Kwun, Y.; Sung, M. , Performance Evaluation of Textile-Based Electrodes and Motion Sensors for Smart Clothing, *IEEE Sensors Journal.* 2011*, 11*, 3183–3193, https://doi.org/10.1109/JSEN.2011.2167508.

[11] Löfhede, J.; Seoane, F.; Thordstein, M. , Textile Electrodes for EEG Recording — A Pilot Study, *Sensors (Basel, Switzerland).* 2012*, 12*, 16907–16919, https://doi.org/10.3390/s121216907.

[12] Cömert, A.; Honkala, M.; Hyttinen, J. , Effect of pressure and padding on motion artifact of textile electrodes, *Biomedical Engineering Online.* 2013*, 12*, 26, https://doi.org/10.1186/1475-925X-12-26.

[13] Nelson, A.; Schmandt, J.; Shyamkumar, P.; Wilkins, W.; Lachut, D.; Banerjee, N.; Rollins, S.; Parkerson, J.; Varadan, V. , Wearable multi-sensor gesture recognition for paralysis patients, *2013 Ieee Sensors.* 2013, 1–4, https://doi.org/10.1109/ICSENS.2013.6688531.

[14] Paul, G.; Torah, R.; Beeby, S.; Tudor, J. , The development of screen printed conductive networks on textiles for biopotential monitoring applications, *Sensors and Actuators. A. Physical.* 2014*, 206*, 35–41, https://doi.org/10.1016/j.sna.2013.11.026.

[15] Paul, G. M.; Fan Cao; Torah, R.; Kai Yang; Beeby, S.; Tudor, J. , A Smart Textile Based Facial EMG and EOG Computer Interface, *IEEE Sensors Journal.* 2014*, 14*, 393–400, https://doi.org/10.1109/JSEN.2013.2283424.

[16] Zhou, Y.; Ding, X.; Zhang, J.; Duan, Y.; Hu, J.; Yang, X. , Fabrication of conductive fabric as textile electrode for ECG monitoring, *Fibers and Polymers.* 2014*, 15*, 2260–2264, https://doi.org/10.1007/s12221-014-2260-y.

[17] Yapici, M. K.; Alkhidir, T.; Samad, Y. A.; Liao, K. , Graphene-clad textile electrodes for electrocardiogram monitoring, *Sensors and Actuators. B, Chemical.* 2015*, 221*, 1469–1474, https://doi.org/10.1016/j.snb.2015.07.111.

[18] Takamatsu, S.; Lonjaret, T.; Crisp, D.; Badier, J.; Malliaras, G. G.; Ismailova, E. , Direct patterning of organic conductors on knitted textiles for long-term electrocardiography, *Scientific Reports.* 2015*, 5*, 15003, https://doi.org/10.1038/srep15003.

[19] Liang, S.; Kuo, C.; Lee, Y.; Lin, W.; Liu, Y.; Chen, P.; Cherng, F.; Shaw, F. , Development of an EOG-Based Automatic Sleep-Monitoring Eye Mask, *IEEE Transactions on Instrumentation and Measurement.* 2015*, 64*, 2977–2985, https://doi.org/10.1109/TIM.2015.2433652.

[20] Matiko, J. W.; Wei, Y.; Torah, R.; Grabham, N.; Paul, G.; Beeby, S.; Tudor, J. , Wearable EEG headband using printed electrodes and powered by energy harvesting for emotion monitoring in

ambient assisted living, *Smart Materials and Structures.* 2015*, 24*, 125028–11, https://doi.org/10.1088/0964-1726/24/12/125028.

[21] Weder, M.; Hegemann, D.; Amberg, M.; Hess, M.; Boesel, L.; Abächerli, R.; Meyer, V.; Rossi, R. , Embroidered Electrode with Silver/Titanium Coating for Long-Term ECG Monitoring, *Sensors (Basel, Switzerland).* 2015*, 15*, 1750–1759, https://doi.org/10.3390/s150101750.

[22] Rui Zhang; Bernhart, S.; Amft, O. , Diet eyeglasses: Recognising food chewing using EMG and smart eyeglasses, *Proceedings (International Conference on Wearable and Implantable Body Sensor Networks : Print).* 2016, 7–12, https://doi.org/10.1109/BSN.2016.7516224.

[23] Boehm, A.; Yu, X.; Neu, W.; Leonhardt, S.; Teichmann, D. , A Novel 12-Lead ECG T-Shirt with Active Electrodes, *Electronics (Basel).* 2016*, 5*, 75, https://doi.org/10.3390/electronics5040075.

[24] Pani, D.; Dessi, A.; Saenz-Cogollo, J. F.; Barabino, G.; Fraboni, B.; Bonfiglio, A. , Fully Textile, PEDOT:PSS Based Electrodes for Wearable ECG Monitoring Systems, *IEEE Transactions on Biomedical Engineering.* 2016*, 63*, 540–549, https://doi.org/10.1109/TBME.2015.2465936.

[25] Karim, N.; Afroj, S.; Malandraki, A.; Butterworth, S.; Beach, C.; Rigout, M.; Novoselov, K. S.; Casson, A. J.; Yeates, S. G. , All inkjet-printed graphene-based conductive patterns for wearable e-textile applications, *Journal of Materials Chemistry C.* 2017*, 5*, 11640–11648, https://doi.org/10.1039/C7TC03669H.

[26] Fleury, A.; Alizadeh, M.; Stefan, G.; Chau, T. , Toward fabric-based EEG access technologies: Seamless knit electrodes for a portable brain-computer interface, *LSC : 2017 IEEE Life Sciences Conference : 13-15 December 2017.* 2017, 35–38, https://doi.org/10.1109/LSC.2017.8268137.

[27] Bihar, E.; Roberts, T.; Ismailova, E.; Saadaoui, M.; Isik, M.; Sanchez-Sanchez, A.; Mecerreyes, D.; Hervé, T.; De Graaf, J. B.; Malliaras, G. G. , Fully Printed Electrodes on Stretchable Textiles for Long-Term Electrophysiology, *Advanced Materials Technologies.* 2017*, 2*, n/a, https://doi.org/10.1002/admt.201600251.

[28] Guo, J.; Yu, S.; Li, Y.; Huang, T.; Wang, J.; Lynn, B.; Fidock, J.; Shen, C.; Edwards, D.; Su, H. , A soft robotic exo-sheath using fabric EMG sensing for hand rehabilitation and assistance, *2018 IEEE International Conference on Soft Robotics (RoboSoft).* 2018, 497–503, https://doi.org/10.1109/ROBOSOFT.2018.8405375.

[29] La, T.; Qiu, S.; Scott, D. K.; Bakhtiari, R.; Kuziek, J. W. P.; Mathewson, K. E.; Rieger, J.; Chung, H. , Two-Layered and Stretchable e-Textile Patches for Wearable Healthcare Electronics, *Advanced Healthcare Materials.* 2018*, 7*, e1801033–n/a, https://doi.org/10.1002/adhm.201801033.

[30] Golparvar, A. J.; Yapici, M. K. , Electrooculography by Wearable Graphene Textiles, *IEEE Sensors Journal.* 2018*, 18*, 8971–8978, https://doi.org/10.1109/JSEN.2018.2868879.

[31] Lee, S.; Kim, M.; Kang, T.; Park, J.; Choi, Y. , Knit Band Sensor for Myoelectric Control of Surface EMG-Based Prosthetic Hand, *IEEE Sensors Journal.* 2018*, 18*, 8578–8586, https://doi.org/10.1109/JSEN.2018.2865623.

[32] Ankhili, A.; Tao, X.; Cochrane, C.; Coulon, D.; Koncar, V. , Washable and Reliable Textile Electrodes Embedded into Underwear Fabric for Electrocardiography (ECG) Monitoring, *Materials.* 2018*, 11*, 256, https://doi.org/10.3390/ma11020256.

[33] Qin, H.; Li, J.; He, B.; Sun, J.; Li, L.; Qian, L. , Novel Wearable Electrodes Based on Conductive Chitosan Fabrics and Their Application in Smart Garments, *Materials.* 2018*, 11*, 370, https://doi.org/10.3390/ma11030370.

[34] An, X.; Stylios, G. K. , A Hybrid Textile Electrode for Electrocardiogram (ECG) Measurement and Motion Tracking, *Materials.* 2018*, 11*, 1887, https://doi.org/10.3390/ma11101887.

[35] Lidón-Roger, J.; Prats-Boluda, G.; Ye-Lin, Y.; Garcia-Casado, J.; Garcia-Breijo, E. , Textile Concentric Ring Electrodes for ECG Recording Based on Screen-Printing Technology, *Sensors (Basel, Switzerland).* 2018*, 18*, 300, https://doi.org/10.3390/s18010300.

[36] Pino, E. J.; Arias, Y.; Aqueveque, P. , Wearable EMG Shirt for Upper Limb Training, *Annual International Conference of the IEEE Engineering in Medicine and Biology Society. IEEE Engineering in Medicine and Biology Society. Annual International Conference.* 2018*, 2018*, 4406–4409, https://doi.org/10.1109/embc.2018.8513107.

[37] Alizadeh-Meghrazi, M.; Ying, B.; Schlums, A.; Lam, E.; Eskandarian, L.; Abbas, F.; Sidhu, G.; Mahnam, A.; Moineau, B.; Popovic, M. R. , Evaluation of dry textile electrodes for long-term electrocardiographic monitoring, *Biomedical Engineering Online.* 2021*, 20*, 68–20, https://doi.org/10.1186/s12938-021-00905-4.

[38] Ohiri, K. A.; Pyles, C. O.; Hamilton, L. H.; Baker, M. M.; McGuire, M. T.; Nguyen, E. Q.; Osborn, L. E.; Rossick, K. M.; McDowell, E. G.; Strohsnitter, L. M.; Currano, L. J. , E-textile based modular sEMG suit for large area level of effort analysis, *Scientific Reports.* 2022*, 12*, 9650–14, https://doi.org/10.1038/s41598-022-13701-4.

[39] Dong, J.; Wang, D.; Peng, Y.; Zhang, C.; Lai, F.; He, G.; Ma, P.; Dong, W.; Huang, Y.; Parkin, I. P.; Liu, T. , Ultra-stretchable and superhydrophobic textile-based bioelectrodes for robust self-cleaning and personal health monitoring, *Nano Energy.* 2022*, 97*, 107160, https://doi.org/10.1016/j.nanoen.2022.107160.

[40] Maithani, Y.; Singh, A.; Mehta, B. R.; Singh, J. P. , PEDOT: PSS treated cotton-based textile dry electrode for ECG sensing, *Materials Today: Proceedings.* 2022*, 62*, 4052–4057, https://doi.org/10.1016/j.matpr.2022.04.611.

[41] Guler, S.; Golparvar, A.; Ozturk, O.; Yapici, M. K. , Ear Electrocardiography With Soft Graphene Textiles for Hearable Applications, *IEEE Sensors Letters.* 2022*, 6*, 1–4, https://doi.org/10.1109/LSENS.2022.3198279.

[42] Etana, B. B.; Malengier, B.; Kwa, T.; Krishnamoorthy, J.; Langenhove, L. V. , Evaluation of Novel Embroidered Textile-Electrodes Made from Hybrid Polyamide Conductive Threads for Surface EMG Sensing, *Sensors (Basel, Switzerland).* 2023*, 23*, 4397, https://doi.org/10.3390/s23094397.

[43] Ali, B.; Cheraghi Bidsorkhi, H.; D'Aloia, A. G.; Laracca, M.; Sarto, M. S. , Wearable graphene-based fabric electrodes for enhanced and long-term biosignal detection, *Sensors and Actuators Reports.* 2023*, 5*, 100161, https://doi.org/10.1016/j.snr.2023.100161.

[44] Yang, J.; Nithyanandam, P.; Kanetkar, S.; Kwon, K. Y.; Ma, J.; Im, S.; Oh, J.; Shamsi, M.; Wilkins, M.; Daniele, M.; Kim, T.; Nguyen, H. N.; Truong, V. K.; Dickey, M. D. , Liquid Metal Coated Textiles with Autonomous Electrical Healing and Antibacterial Properties, *Advanced Materials Technologies.* 2023*, 8*, n/a, https://doi.org/10.1002/admt.202202183.

[45] Lim, T.; Kim, H. J.; Won, S.; Kim, C. H.; Yoo, J.; Lee, J. H.; Son, K. S.; Nam, I.; Kim, K.; Yeo, S. Y.; Yeang, B. J.; Kim, J. H.; Zhang, H.; Lee, S. , Liquid Metal-Based Electronic Textiles Coated with Au Nanoparticles as Stretchable Electrode Materials for Healthcare Monitoring, *ACS Applied Nano Materials.* 2023*, 6*, 8482–8494, https://doi.org/10.1021/acsanm.3c00761.

[46] Zhao, J.; Kim, C.; Li, W.; Wen, Z.; Xiao, Z.; Wang, Y.; Chakrabartty, S.; Wang, C. , 3D E-textile for Exercise Physiology and Clinical Maternal Health Monitoring, *ArXiv.Org.* 2024, https://doi.org/10.48550/arXiv.2407.07954.

[47] Komal, K.; Cleary, F.; Narayanan, R. P.; Wells, J.; Buiatti, M.; Bennett, L. , Design and Validation of a Modular Smart Headband with Embroidered Electrodes for Comfortable EEG Monitoring, 2026, https://arxiv.org/abs/2511.19348.

[48] Qiu, S.; Li, Y.; Dai, C.; Wu, S.; Chen, X.; Ji, N.; Fang, G.; Yam, Y.; Wang, C. C. L.; Zhao, N. , A textile-based alignment-free electrophysiological sensing sleeve for comprehensive cardiovascular monitoring, *Microsystems & Nanoengineering.* 2025*, 11*, 228–11, https://doi.org/10.1038/s41378-025-01088-x.
