# Supplementary material for: Body surface potential mapping of the cortico-muscular axis using smart textile electrode arrays
Source: Nat Commun. 2026 Jul 8;17:8441. doi: 10.1038/s41467-026-75134-1 (PMC13478180; doi:10.1038/s41467-026-75134-1)
Supplement: Supplementary file 2 [file 41467_2026_75134_MOESM2_ESM.pdf]

nature portfolio

Peer Review File

Body Surface Potential Mapping of the Cortico-Muscular Axis Using Smart Textile Electrode Arrays

Corresponding Author: Professor George Malliaras

This file contains all reviewer reports in order by version, followed by all author rebuttals in order by version.

Version 0:

Reviewer comments:

Reviewer #1

(Remarks to the Author)
The authors present a multimodal electrophysiology platform enabled by a new scalable smart-textile fabrication process. They integrate conformable EEG, EMG, ECG, and EOG electrodes into flexible arrays, validate recordings across modalities, and demonstrate advanced applications including machine-learning–based classification and simultaneous mapping of cortical and muscular activity. The ability to predict spatial EMG activation patterns from cortical spectral features and to extract reaction-time distributions along the cortico–muscular axis is presented as the first demonstration using independently optimized textile EEG and EMG arrays. The work addresses an important challenge in wearable neurotechnology: unifying multiple electrophysiological sensing modalities in a single fabric-based platform. The results are promising and have potential impact for motor-intent decoding, neuroprosthetic development, and broader multimodal physiological monitoring. That said, significant clarifications and additional substantiation are needed before the manuscript can be considered for publication.
1.The authors claim the fabrication approach is “novel” and “scalable,” but the manuscript does not sufficiently differentiate it from existing smart-textile, dry EEG, or flexible EMG/electrode technologies. It is essential that the authors provide: 1. A clear comparison to previous textile-based electrophysiology systems. 2. Evidence that the proposed process offers superior mechanical robustness, recording stability, or manufacturability. Without this contextualization, the conceptual advance remains difficult to evaluate.
2.The manuscript lacks rigorous characterization of electrode performance: 1. Long-term stability under mechanical strain, laundering, perspiration, or temperature changes needs more details. 2. Variability across electrodes and between batches of fabricated arrays is not quantified. 3. Comparisons to standard Ag/AgCl electrodes are limited.
3.While the authors demonstrate recordings of ECG, EMG, EOG, and EEG, the signal quality assessment is incomplete: 1. Motion artefacts, especially for EEG on a textile substrate, need systematic characterization.2. Potential cross-talk between modalities is neither quantified nor discussed. 3. It is unclear how referencing schemes, filtering, or preprocessing were optimized for each modality. 4. The authors should include SNR metrics, impedance profiles, and artefact rejection performance for each modality.
4.The authors report machine-learning–based shape and sensorimotor cue classification, yet: The sample size is small, and cross-validation procedures are not fully described. It is unclear whether results generalize across subjects or only within subjects. No ablation studies are performed to identify feature importance or impact of electrode positioning. The machine-learning pipeline requires more transparency to ensure reproducibility. These analyses are required to substantiate the claims of “high fidelity” classification.
5.The reported ability to map reaction-time distributions and predict EMG spatial activation from EEG spectral features is compelling, but the manuscript lacks: A clear explanation of the causal assumptions underlying these analyses. Quantitative metrics of prediction accuracy and how they compare to baseline or chance levels. Controls to exclude confounds (e.g., motion artefacts driving both EEG and EMG). Given the significance of this claim, stronger statistical and mechanistic evidence is needed.
6.Several grammatical and typographical errors are present throughout the manuscript. A thorough proofreading and polishing of the English language are necessary before resubmission. Additionally, reference formatting should be standardized according to the journal's guidelines, ensuring consistency across all entries. Where possible, update DOIs with page numbers for completeness.

(Remarks on code availability)

Reviewer #2

(Remarks to the Author)
The paper presents the development of a wearable flexible hybrid e-textile based sensors platform for the simultaneous mapping of electrical activity along the cortico-muscular axis. The device fabrication process involves multiple coating steps of conducting and insulating flexible materials onto fabric to create a multi-layered and vertical stacked system that includes: electrode array, tracks, and interconnection system to plug-in the customized PCB. The system is completed with a customized wireless transmission unit. The authors used this platform for multi-modal electrophysiological cutaneous recording. Specifically, the authors used electrodes to record EMG, EOG and EEG. Multi electrodes arrays were used to extract spatiotemporal features from EMG, from extrinsic hand muscles, and EEG to accurately classify the grasped object shapes and somatosensory stimuli. The authors then showed the multimodal simultaneous recording of the hand muscular activity and the brain electrical activity: the cortico-muscular axis was studied by generating spatial maps of reaction time distribution when hand performed specific tasks. By employing machine learning algorithms, the EEG cortical power features were used to predict muscle activation maps, demonstrating the collected data can be used not only for classification tasks, but also to develop predictive modelling of neuromuscular coordination and cortico-muscular reaction time.

The study is very interesting and timely, addressing some unmet needs in neurotechnologies, with a very hogh potential impact in applications like neurorehabilitation, prosthetics and human-machine interfaces. Particulary important is the use of BSPM and the development of training models.

Despite these positive notes the manuscript presents several weaknesses. First, the experimental description is quite poor on various aspects, regarding the fabrication and the recording protocols: these are listed in detail. Some claims about conformability, wearability are not supported by clear evidences and characterization measurements. With respect to state of the art, what are the unique characteristics that distinguish this platform? The authors should highlight more and indicate which features (Fabrication? Materials? Wearability? Other?) allow to overcome limitations of current technologies (a clear and quantitative comparison would be important). More care overall is needed for the figures (Especially figure 1) and for the captions that not always describe clearly the figures content, making the understanding difficult and leaving ambiguities.

Overall we suggest to consider making major revisions to the current manuscript before to accept it for publication.

Comments/questions:

1. Which are the limitations of the standard methods (It is not well described to which standard methods are you comparing your technology to)? Does your technology overcome their spatial resolution and performance, wiring complexity, wearability, data transmission and interpretability limitations? Comparison with the SoA?

2. The use of different designs for different type of recording/locations on the body is very important. However, the manuscript does not clearly decsribe each design and explain the choices made (e.g. electrode size and spacing, number, use of flexible or rigid PCB, etc). There is not a clear description of the fabrication steps: figure 1 unfortunately is very generic and the photos there do not clearly indicate the steps. We suggest to number the steps/figures and accompany with a schematics/cartoon. Also, the other designs (head, forearm, forehead) are not decsribed in detail: what are the differences there? How do they look when worn on skin? Even in Supp Info this is not really clear, as the arrays are covered by unknown “flexible band” (what is that?).

3. Similar ambiguity in experimental part is about materials. For some of them (e.g. PEDOT:LS) the origin is unknown. Also the steps for synthesis and fabrication of ETG electrodes is unsufficient: how they are fabricated? What is their size? In the result section there is this description: “To deposit the coating, the fabric is inverted, a fixed amount of ETG is dispensed onto each electrode site and the coating is melted in an oven and cooled to solidify. The completed array, including top and bottom views, can be seen in Fig. 1c” This is unsufficient: which quantity? What temperature? And so on. Also, the benefit of ETG should be highlighted, in comparison with standard sensor surfaces.

4. Still in Experimental the description of protocols used in recordings is unsufficient: how the various electrode arrays are placed and fixed on the various body locations? Where? On how many subjects? No evidence of statistics is reported! Adhesion on the skin: how is it provided and maintained? How do you ensure the relative displacement of the system during movement?

5. Reread carefully all the experimental and complete the missing information: it is hard to assess the quality of your work and/or to replicate your experiment without a proper description.

6. The benefits of your method to deposit ink on the textile and layering with rubber are not clear. What do you mean with the sentence “signals, enabling high-density layouts without damaging the textile or being constrained by track spacing”? Also the claim of “high density” is questionable. Can you argument on that?

7. The layered structure depicted in Figure 1 comprise several layers of PDMS, Epoxy Ag, Ag paste, ETG, textile, PCB. Nothing is said about their thickness and about their machanical properties: is the overall system stretchable? Or flexible? How much? From the pictures the system looks pretty bulky and rigid. So also the claim about conformability is

questionable.

8. In figure 1 a it is not clear which images are the real photos (and they are quite small!). It seems one image is repeated 3 times. Consider change the figure with something more clear and readable.

9. The quality of the ECG trace in fig.2 it is not so high. These electrodes are novel, you may add a trace recorded with standard electrodes for comparison.

10. In the EMG trace in Fig.2 it is not clear when the wrist is flexed and for how many times. Did the subject continuously flex and extend the wrist for 4 seconds? If yes, at which rate? The description of the task should be better reported. Where is the EMG envelope graph?

11. In the EOG trace in Fig.2 it is not clear either the subject only blinked or kept periodically the eye closed and opened.

12. For the EEG recorded from 16 electrodes, did you use any gel electrolyte? Does the Gly:ChCl and gelatine system remains hydrated over time?

13. The lower muscular SNR in EMG recording during hand flexion is ascribable to what?

14. How many recording/subjects did you use for training the model?

15. A quantification about the conformability and robustness of the electrode platform would be need to position your device among other SoA devices.

(Remarks on code availability)

Reviewer #3

(Remarks to the Author)
I co-reviewed this manuscript with one of the reviewers who provided the listed reports. This is part of the Nature Communications initiative to facilitate training in peer review and to provide appropriate recognition for Early Career Researchers who co-review manuscripts.

(Remarks on code availability)

Reviewer #4

(Remarks to the Author)
In this study, the authors present a novel system integrating advanced electrode coatings and a flexible e-textile design, enabling separate or simultaneous recordings along the cortico-muscular axis. While initial testing is provided, it remains unclear whether the system offers novel features or advantages over existing multichannel EEG/EMG setups.

The authors recorded brain responses to visual, motor, and auditory stimuli, which inherently have well-defined temporal structures. However, no evoked responses are shown, and I strongly recommend including them.

Since EEG spectra have both periodic and aperiodic components—where the aperiodic part often corresponds to noise—it would be beneficial to present spectra with noise estimates.
The relationship between the provided confusion matrices and induced versus evoked EEG activity is unclear; clarification and separate classification results for both types would strengthen the study.

For demonstrating EEG modulation by movement, I suggest including ERD/ERS analyses of alpha and beta oscillations in time domain, as such data are highly informative for neuroscientists and physiologists.

Notably, despite discussing cortico-muscular coherence (CMC) in the introduction, the authors do not compute it directly, instead analyzing power spectra. Since CMC, especially from raw EEG and EMG signals, could highlight the benefits of the new electrode arrays by revealing distinct activation patterns, including it would significantly enhance the manuscript. This is particularly important given that similar recordings exist (Vidaurre et al., 2019), so the authors should clarify the novelty of their approach. Lastly, presenting direct comparisons showing that their system outperforms current multielectrode methods would provide persuasive evidence of its value.

Vidaurre, C., Nolte, G., de Vries, I. E. J., Gómez, M., Boonstra, T. W., Müller, K. R., Villringer, A., & Nikulin, V. V. (2019). Canonical maximization of coherence: A novel tool for investigation of neuronal interactions between two datasets. Neuroimage, 201, 116009. doi:10.1016/j.neuroimage.2019.116009

(Remarks on code availability)

I was asked to assess neurophysiological plausibility of the study

Version 1:

Reviewer comments:

Reviewer #1

(Remarks to the Author)
The authors well addressed all my previous concerns that I would like to recommend it to be accepted.

(Remarks on code availability)

Reviewer #2

(Remarks to the Author)
Authors replied extensively and satisfactorily to the various reviewers' comments. They also implemented relevant changes which improved considerably the quality and clarity of the manuscript.
Therefore I recommend accepting it for publication.

(Remarks on code availability)

Reviewer #3

(Remarks to the Author)
I co-reviewed this manuscript with one of the reviewers who provided the listed reports. This is part of the Nature Communications initiative to facilitate training in peer review and to provide appropriate recognition for Early Career Researchers who co-review manuscripts.

(Remarks on code availability)

Reviewer #4

(Remarks to the Author)
Thank you for addressing many of my concerns. However, I am surprised that the authors did not record triggers for the evoked responses. Since evoked responses represent one of the most important measures obtained with EEG, it is very important to show typical waveforms for auditory and visual responses. Bar plots do not provide sufficient information and I am surprised for instance that visual responses have the strongest amplitude over the central areas while they should be largest over parieto-occipital areas (Figure S32). I strongly encourage authors to provide traditional waveforms and topographies of the evoked responses.

Figure S33. Is it only one subject? It would be nice to see classic alpha oscillations in the recordings. At the moment I do not understand whether it is just one specific example or whether electrodes can’t record traditional pronounced alpha oscillations.

(Remarks on code availability)

Open Access This Peer Review File is licensed under a Creative Commons Attribution 4.0 International License, which permits use, sharing, adaptation, distribution and reproduction in any medium or format, as long as you give appropriate credit to the original author(s) and the source, provide a link to the Creative Commons license, and indicate if changes were made.
In cases where reviewers are anonymous, credit should be given to 'Anonymous Referee' and the source.
The images or other third party material in this Peer Review File are included in the article's Creative Commons license, unless indicated otherwise in a credit line to the material. If material is not included in the article's Creative Commons license and your intended use is not permitted by statutory regulation or exceeds the permitted use, you will need to obtain permission directly from the copyright holder.
To view a copy of this license, visit https://creativecommons.org/licenses/by/4.0/

RESPONSE TO REVIEWER COMMENTS

The authors would like to thank all reviewers for their insightful and very relevant comments.

Reviewer #1 (Remarks to the Author):

1. The authors claim the fabrication approach is "novel" and "scalable," but the manuscript does not sufficiently differentiate it from existing smart-textile, dry EEG, or flexible EMG/electrode technologies. It is essential that the authors provide: 1. A clear comparison to previous textile-based electrophysiology systems. 2. Evidence that the proposed process offers superior mechanical robustness, recording stability, or manufacturability. Without this contextualization, the conceptual advance remains difficult to evaluate.

   We have revised the main text to clarify claims on novelty, emphasising that the contribution of this work lies not in individual fabrication steps but in the practical integration of multimodal EEG and EMG recording in a single, wearable platform. We also highlight that the fabrication processes we use, blade coating, PDMS lamination, laser-patterned masking, and eutectogel deposition, are scalable and compatible with standard industrial techniques such as screen printing and additive manufacturing. This ensures that our approach is not only technically feasible but also practically deployable for larger-scale production (pages 2-5).

   Regarding the comparison to previous textile-based electrophysiology systems, we have included an extensive literature review in Supplementary Table S1. This table situates our work within the landscape of existing wearable technologies, comparing single-modality versus multimodal designs, sparse versus dense electrode arrays, and the presence of multilayer or via-based interconnects. While stacked conductors and vertical interconnects are standard in semiconductor fabrication, they are rarely realised on textile substrates. Our platform translates these concepts to a gel-free, wearable format, enabling simultaneous multimodal recordings across cortical and muscular domains with integrated multilayer interconnects that are not accessible with conventional textile methods.

   With respect to mechanical robustness and signal quality, the orthogonal, multi-material fabrication process produces electrodes that are resilient under bending and repeated handling. We have performed mechanical characterisation, including 4-point bending and minimum bending radius tests, inter-array and inter-device impedance measurements, and simulated motion artifact experiments comparing our ETG-coated electrodes to commercial Ag/AgCl electrodes (Supporting Information Figure S5-S10). These tests demonstrate stable recordings under realistic motion conditions, confirming that the integrated platform maintains signal quality in practical scenarios. Combined with the compatibility of all fabrication steps with scalable techniques, these features underscore a practical, spatially dense, multimodal wearable system that overcomes limitations of prior textile-based platforms in wiring complexity, wearability, and recording stability.

2. The manuscript lacks rigorous characterization of electrode performance: 1. Long-term stability under mechanical strain, laundering, perspiration, or temperature changes needs

more details. 2. Variability across electrodes and between batches of fabricated arrays is not quantified. 3. Comparisons to standard Ag/AgCl electrodes are limited.

We have expanded the characterisation of electrode performance in the revised manuscript and Supplementary Information to provide a clearer assessment of signal quality and stability. For mechanical robustness, electrodes were subjected to controlled 4-point bending tests, including cyclic loading at a bending radius of (MBR + 1 mm) for 100 cycles, as well as simulated motion artifact experiments. These tests demonstrate stable connectivity, impedance, and signal quality under repeated deformation. Inter-electrode variability within arrays and reproducibility across independently fabricated batches have also been quantified, showing consistent electrical performance. Evaluation under laundering, perspiration exposure, and temperature cycling was not included in this study, as the focus is on short- to medium-duration experimental use; these aspects will be addressed in future work aimed at chronic and daily-life deployment, as noted in the Conclusion (pages 2-5 and 14).

Regarding comparisons to standard Ag/AgCl electrodes, our previous work has extensively characterised the PEDOT:LS ETG electrode material in terms of impedance and SNR for cutaneous recordings in healthy volunteers, demonstrating that the SNR obtained is effectively equivalent to that of conventional Ag/AgCl electrodes with approximately fourfold larger surface area for both EMG and ECG (DOI:10.1002/admt.202101680, DOI:10.1002/advs.202301176). These earlier studies also showed stable signal quality across recordings spaced over multiple weeks. In response to the reviewer's request for additional characterisation, we have clarified reference to these previous works and performed complementary experiments to evaluate mechanical robustness and signal stability under realistic motion conditions. These results, detailed in Supplementary Information Figure S9 and S10, demonstrate that the electrodes maintain consistent, gel-free signal acquisition with high spatial resolution during typical experimental use, thereby addressing concerns regarding short-term stability and reliability compared to commercial electrodes.

3. While the authors demonstrate recordings of ECG, EMG, EOG, and EEG, the signal quality assessment is incomplete: 1. Motion artefacts, especially for EEG on a textile substrate, need systematic characterization.2. Potential cross-talk between modalities is neither quantified nor discussed. 3. It is unclear how referencing schemes, filtering, or preprocessing were optimized for each modality. 4. The authors should include SNR metrics, impedance profiles, and artefact rejection performance for each modality.

We have now systematically addressed signal quality and artefact resilience in the revised manuscript. Motion artefacts were evaluated by performing simulated motion experiments comparing our ETG-coated electrodes with commercial Ag/AgCl electrodes and bare gold electrodes, demonstrating that our system maintains stable recordings under realistic conditions (Supporting Information Figure S10).

Regarding cross-talk, we have included clarifications in the main text that for single-array recordings (Fig. 2), multiple modalities (ECG, EMG, EOG and EEG) were acquired in separate trials, with only EEG and EOG recorded simultaneously. Potential cross-talk between EEG and EOG signals were minimised by filtering EEG to reduce ocular artefacts, ensuring minimal interference. For simultaneous EEG-EMG experiments,

independent arrays were used with separate digitisation and transmission paths, eliminating the possibility of any cross-talk between modalities (page 7).

We have expanded the Methods section to provide detailed descriptions of referencing schemes, filtering, and preprocessing steps for each modality. For the array in Fig. 2, the size of the electrodes was optimised for ECG recordings, and the corresponding SNR for commercial and eutectogel electrodes has been computed and included in the main text (page 7). The same array was subsequently used to record EMG, EEG, and EOG signals to demonstrate the platform's capability for multimodal acquisition. SNR values for EMG and EEG/EOG are not reported, as they would not be representative: for EMG, the relatively large electrode size attenuates high-frequency components of the signal, and for EEG/EOG, the same footprint increases impedance relative to commercial electrodes. The intention of these recordings is to illustrate the versatility of the system across multiple bio-signals rather than to provide absolute benchmarking of SNR for each modality.

As the same electrodes were used across modalities, impedance profiles are consistent with previously published characterisations of PEDOT:LS ETG electrodes, and a clearer reference to these prior measurements has been added (page 5).

4. The authors report machine-learning–based shape and sensorimotor cue classification, yet: The sample size is small, and cross-validation procedures are not fully described. It is unclear whether results generalize across subjects or only within subjects. No ablation studies are performed to identify feature importance or impact of electrode positioning. The machine-learning pipeline requires more transparency to ensure reproducibility. These analyses are required to substantiate the claims of "high fidelity" classification.

We have clarified in the main text that all machine-learning experiments were performed on a single participant, and thus our results demonstrate within-subject performance only; generalisation across subjects cannot be claimed at this stage. For each movement or stimulus class, we collected 100 samples, which is sufficient to ensure stable estimates of classifier performance under the central limit theorem, though not to claim broader subject-level generalisation. This has been clarified throughout the main text and methods (page 11).

To provide further insight into feature relevance, we have included correlation circles highlighting which spatially distributed electrodes contributed most to classification in the Supplementary Information Figure S31. Electrode position can indeed affect model performance because different electrodes record activity from distinct muscle fibres, producing variations in BSP maps. During repeated measurements, array placement was maintained as consistently as possible, but small displacements of approximately 1 cm, particularly along the forearm axis, occurred. The trained algorithms were able to account for these minor positional variations, demonstrating robustness to minor electrode displacement. A similar principle applies to EEG recordings. This discussion has been added to the main text (page 11).

Finally, we have expanded the Methods section to provide more details on the machine-learning pipeline, including feature extraction, preprocessing, and cross-validation procedures, to improve transparency and reproducibility of the analyses.

5. The reported ability to map reaction-time distributions and predict EMG spatial activation from EEG spectral features is compelling, but the manuscript lacks: A clear explanation of the causal assumptions underlying these analyses. Quantitative metrics of prediction accuracy and how they compare to baseline or chance levels. Controls to exclude confounds (e.g., motion artefacts driving both EEG and EMG). Given the significance of this claim, stronger statistical and mechanistic evidence is needed.

   We have revised the manuscript to address the reviewer's concerns regarding causal assumptions, prediction metrics, and potential confounds. The causal framework has been clarified: EEG activity is interpreted as cortical motor planning preceding EMG activation, and analyses align EEG segments to EMG events to map temporal relationships while acknowledging limitations in causal inference. Furthermore, Supplementary Figure S34 provides a classification performance comparison between evoked and induced activity for EEG classification, further demonstrating the causal nature of the experiments performed (evoked classification is near-perfect, whereas induced classification performs very poorly) (pages 11-12).

   Prediction accuracy is quantified using three complementary metrics: Pearson correlation, Spearman rank correlation, and cosine similarity. We have now compared to baseline chance levels estimated via trial permutation. Chance performance produces near-zero correlations and cosine similarity of ~0.5. These additions demonstrate that predicted EMG maps reproduce both amplitude and spatial patterns well above chance (Pearson: 0.80–0.83, Spearman: 0.73–0.80, Cosine similarity: 0.95–0.99) (pages 13-14).

   To address potential confounds from motion artefacts, we performed simulated motion tests, comparing textile electrodes to metal and Ag/AgCl electrodes (Supplementary Information Figure S10), showing stable connectivity and signal quality. Preprocessing with rolling mean smoothing and Z-score normalisation further mitigates baseline drift and high-frequency noise.

6. Several grammatical and typographical errors are present throughout the manuscript. A thorough proofreading and polishing of the English language are necessary before resubmission. Additionally, reference formatting should be standardized according to the journal's guidelines, ensuring consistency across all entries. Where possible, update DOIs with page numbers for completeness.

   We have thoroughly revised the manuscript for clarity, grammar and formatting consistency, as requested.

Reviewers #2 and #3 (Remarks to the Author):

1. Which are the limitations of the standard methods (It is not well described to which standard methods are you comparing your technology to)? Does your technology overcome their spatial resolution and performance, wiring complexity, wearability, data transmission and interpretability limitations? Comparison with the SoA?

   To clarify the reference frame and limitations of existing approaches, we have added an extensive comparison with representative textile-based and flexible wearable electrophysiology systems in Supplementary Table S1. In the revised manuscript, "standard methods" refers to commonly reported knitted, embroidered, sewn, or surface-

printed textile electrodes and flexible dry or gel-based sensors, which are typically optimised for a single modality or anatomical site, lack spatially dense electrode arrays, and rarely support simultaneous multimodal recording. As highlighted in the table, most prior systems also rely on external wiring, sparse interconnect strategies, or rigid components that limit scalability, wearability, and experimental flexibility. Our approach addresses this specific combination of limitations by integrating spatially resolved EEG and EMG arrays within a unified, gel-free textile architecture featuring multilayer routing and vertical interconnects, enabling simultaneous multimodal body surface potential mapping. The Introduction has been revised to explicitly reference Supplementary Table S1 and to clarify this positioning relative to the current state of the art (page 2).

2. The use of different designs for different type of recording/locations on the body is very important. However, the manuscript does not clearly describe each design and explain the choices made (e.g. electrode size and spacing, number, use of flexible or rigid PCB, etc). There is not a clear description of the fabrication steps: figure 1 unfortunately is very generic and the photos there do not clearly indicate the steps. We suggest numbering the steps/figures and accompany with a schematics/cartoon. Also, the other designs (head, forearm, forehead) are not described in detail: what are the differences there? How do they look when worn on skin? Even in Supp Info this is not really clear, as the arrays are covered by unknown "flexible band" (what is that?).

   We have included a detailed rationale for the linear 5-electrode array design in the main text. Additionally, the optimal design parameters for both 16-electrode EEG and EMG arrays were computed strictly following the framework and analytical procedure described in DOI:10.1002/advs.202411087. The Methods section now provides full details of these parameter choices and this is explicitly referenced in the main text (page 7).

   Figure 1 has been modified to increase readability and provide clarity on the fabrication process. As suggested, individual steps in the fabrication are shown. The fabrication steps have been described briefly in the main text and a more detailed description of the fabrication process has been provided in the Methods section (pages 3-5).

   In addition, we have clarified the nature of the "flexible band" used in the wearable configurations. The band is a Velcro strap to which the textile electrode array is sewn, providing stable fixation and uniform contact pressure during use. For the experiments reported here, the strap was designed to cover the full surface of the array while leaving the insertion region for the recording electronics exposed. We have added new supplementary figures showing the 16-electrode EMG BSPM array without the strap to clearly illustrate electrode layout and spacing (Figures S27-S28). For the EEG measurements, the arrays were integrated into commercially available rugby headcaps to ensure even pressure distribution across the scalp; as a result, photographs of the EEG arrays without the headcap during wear are not available (Figure S29).

3. Similar ambiguity in experimental part is about materials. For some of them (e.g. PEDOT:LS) the origin is unknown. Also the steps for synthesis and fabrication of ETG electrodes is insufficient: how are they fabricated? What is their size? In the result section there is this description: "To deposit the coating, the fabric is inverted, a fixed amount of

ETG is dispensed onto each electrode site and the coating is melted in an oven and cooled to solidify. The completed array, including top and bottom views, can be seen in Fig. 1c" This is insufficient: which quantity? What temperature? And so on. Also, the benefit of ETG should be highlighted, in comparison with standard sensor surfaces.

We have now included a more detailed description of the deposition process of ETG in the main text. Each electrode received approximately 0.15 g of ETG per 10 mm diameter site (≈0.00191 g/mm²). The material was placed in its solid state at room temperature over the electrode surface and melted in a convection oven at 105 °C, allowing it to reflow into a stable, drop-like morphology confined to the electrode footprint, and cooled at room temperature to solidify. The resulting protrusion above the textile surface was measured as 1 mm ± 5 µm with a Veeco Dektak 6M Stylus Surface Profilometer (pages 5-6).

The Methods section has been expanded to provide detailed quantitative and procedural information on ETG synthesis and deposition. Specifically, we now report the exact component concentrations, temperatures, stirring conditions, and storage steps used to prepare the PEDOT:LS-based eutectogel. Additionally, we specify the mass of ETG deposited per electrode and the electrode dimensions, ensuring reproducibility and clarity for future replication of the fabrication process.

Since our group began studying ionic materials for electrophysiology in 2015, a wide range of material systems has been investigated, including pure cholinium-based ionic materials (10.1039/c5tc01888a), PEDOT combined with ionic liquids and polysaccharides (10.1021/acsmacrolett.7b00104; 10.1021/acsomega.8b00458), polycarbonates combined with ionic liquids (10.3390/polym10090989), and PEDOT blended with ionic liquids (10.1002/adhm.202100374; 10.1039/d2tc03248a). Building on this accumulated know-how, we have identified deep eutectic solvent (DES)–based systems as the most advantageous platform for wearable electrophysiological interfaces. When combined with PEDOT, DES form mixed ionic–electronic conductive eutectogels that exhibit high ionic and electronic conductivity, exceptional mechanical flexibility and stretchability, strong adhesion energy, and long-term signal stability exceeding 90 days without SNR degradation (10.1002/advs.202306424; 10.1016/j.biomaterials.2024.122624). In particular, gelatin-based PEDOT eutectogels have demonstrated the best overall performance among the material systems studied to date (10.1002/advs.202301176), owing to their soft, tissue-mimetic nature and their ability to conform intimately to the skin, thereby improving electrode–skin coupling and reducing motion artefacts compared to conventional dry electrodes or gel-based metal sensors. The non-volatile, low-cost, and biocompatible nature of DES enables stable, gel-free operation, while the compatibility of eutectogels with additive manufacturing and printing techniques (10.1016/j.biomaterials.2024.122624; 10.1039/D3MH00310H) supports scalable fabrication of high-density electrode arrays with improved spatial resolution. Collectively, these properties justify the use of eutectogels as a coating for textile electrode arrays, providing a robust and environmentally friendly alternative to standard sensor surfaces for long-term, high-quality electrophysiological recordings. We have expanded the main text to include this information (page 5).

4. Still in Experimental the description of protocols used in recordings is insufficient: how the various electrode arrays are placed and fixed on the various body locations? Where? On how many subjects? No evidence of statistics is reported! Adhesion on the skin: how is it provided and maintained? How do you ensure the relative displacement of the system during movement?

   We have now substantially revised and expanded the "Experimental protocols and data collection" section to provide a detailed and explicit description of how each electrode array was positioned, fixed, and used for the different recording modalities and figures. The revised text specifies anatomical placement, fixation methods (Velcro strap, or headcap), task timing, trial structure, stimulus characteristics, and the number of repetitions. We also clarify that all experiments were performed on a single participant with repeated trials to assess within-subject consistency, in line with the scope of this study.

5. Reread carefully all the experimental and complete the missing information: it is hard to assess the quality of your work and/or to replicate your experiment without a proper description.

   We have thoroughly revised and expanded the methods section, as requested.

6. The benefits of your method to deposit ink on the textile and layering with rubber are not clear. What do you mean with the sentence "signals, enabling high-density layouts without damaging the textile or being constrained by track spacing"? Also the claim of "high density" is questionable. Can you argument on that?

   The original sentence mentioned was meant to convey that our hybrid e-textile fabrication strategy, blade-coating conductive ink into the porous textile and encapsulating tracks with elastomeric lamination, allows vertical electrical interconnects through the fabric without mechanically damaging the fibres or relying on rigid via structures. This approach decouples electrode placement from lateral routing constraints imposed by conventional stitched, embroidered, or surface-printed interconnects. In other words, the electrodes can be positioned more freely across the textile area without being limited by track routing geometry, while maintaining robust electrical connectivity. We have modified the sentence and provided a more detailed explanation (page 3).

   Regarding "high-density", we have replaced this term with "spatially dense" and clarified that it is used relative to conventional textile interconnect strategies: the layouts we achieve exceed what is practically achievable with stitched, embroidered, or surface-printed electrodes for a given textile area, without implying the densities typical of silicon microelectrode arrays. The revised text explicitly states that "spatially dense" refers to layouts that allow more electrodes per unit area than traditional textile-based approaches, facilitated by the vertical ink infiltration and elastomeric encapsulation, which prevent lateral shorting, delamination, or cracking (page 3).

7. The layered structure depicted in Figure 1 comprise several layers of PDMS, Epoxy Ag, Ag paste, ETG, textile, PCB. Nothing is said about their thickness and about their mechanical properties: is the overall system stretchable? Or flexible? How much? From the pictures the system looks pretty bulky and rigid. So also the claim about conformability is questionable.

The thickness of each layer has now been provided in the Methods section, with key values mentioned in the main text (e.g., total device thickness of 2 mm ± 0.5 mm and ETG electrode protrusion of 1 mm ± 5 µm) (page 3).

We clarify that the system is flexible but intentionally non-stretchable. The non-stretchable design is intentional to preserve fixed inter-electrode distances (IEDs), ensuring reproducible spatial sampling and reliable mapping of distributed bioelectric signals across the body. This maintains high fidelity in spatial resolution, which would be compromised if the substrate were elastically deformable (page 6).

Flexibility has been quantified via the minimum bending radius at which electrical performance remains stable, measured as 0.5 mm for silver-track arrays and 1 mm for FPCB-track arrays. Both values fall within the standard conformability range for wearable devices, typically defined as bending radii between 0.5 and 2 mm (10.3390/bios12080630, 10.1002/advs.202509934, 10.70401/bmeh.2025.0004). These bending tolerances allow the device to conform to typical anatomical surfaces, such as the forearm, chest, or forehead, without compromising electrical performance, which we adopt here as a practical definition of conformability (page 6).

8. In Figure 1a, it is not clear which images are the real photos (and they are quite small!). It seems one image is repeated 3 times. Consider change the figure with something clearer and more readable.

   We agree with the reviewer's comment and have revised Figure 1a to make the fabrication workflow clearer.

9. The quality of the ECG trace in Fig.2 it is not so high. These electrodes are novel, you may add a trace recorded with standard electrodes for comparison.

   To provide a direct comparison, we have included a reference ECG recording obtained with a commercial Ag/AgCl electrode (Meditrace) connected to the same acquisition system, as suggested. The SNR was computed for both electrodes: 19.5 dB for our ETG textile electrode, compared to 15.4 dB for the commercial Ag/AgCl electrode. This demonstrates that, despite the smaller size and gel-free application, the ETG electrodes achieve comparable signal quality to standard clinical electrodes (page 8).

   Furthermore, our previous work has extensively characterised the PEDOT:LS ETG electrode material in terms of impedance and SNR for cutaneous recordings in healthy volunteers, demonstrating that the SNR obtained is effectively equivalent to that of conventional Ag/AgCl electrodes with approximately fourfold larger surface area for both EMG and ECG (DOI:10.1002/admt.202101680, DOI:10.1002/advs.202301176). We have clarified this comparison in the main text to emphasise that the quality of the ECG trace is consistent with standard clinical electrodes (page 5).

10. In the EMG trace in Fig.2 it is not clear when the wrist is flexed and how many times. Did the subject continuously flex and extend the wrist for 4 seconds? If yes, at which

rate? The description of the task should be better reported. Where is the EMG envelope graph?

We have clarified the description of the EMG task in the Methods section. During the recording shown in Fig. 2c, the participant rested their right arm on a table with the palm facing up. After a 10 s baseline (only the last second is displayed in Fig. 2), they performed a single, continuous wrist flexion of 4 s duration, followed by 5 s of rest (only the first second is displayed in Fig. 2). This procedure was repeated for a total of three trials. The rate of motion was approximately 90° wrist flexion over 4 s, corresponding to a slow, controlled contraction.

The EMG trace in Fig. 2c shows the raw EMG signal. The processed EMG envelope has now been included in the Supplementary Fig. S26. The envelope was obtained by rectifying the raw signal and applying a 500-sample moving average, as described in the Methods section.

11. In the EOG trace in Fig.2 it is not clear either the subject only blinked or kept periodically the eye closed and opened.

    We have now clarified this in the main text and Methods. The participant performed voluntary, unsustained blinks during the EOG/EEG recording, alternating with natural eye opening. Each trial consisted of 30 s of blinking at self-paced intervals followed by 30 s of rest, across three trials, as now described in the Methods (page 8).

12. For the EEG recorded from 16 electrodes, did you use any gel electrolyte? Does the Gly:ChCl and gelatine system remains hydrated over time?

    No external gel electrolyte was used for any electrophysiological recording. All electrodes were operated in a gel-free configuration using PEDOT-based eutectogels composed of PEDOT:LS, glycerol/choline chloride (Gly:ChCl) and gelatin. Although gel-free in use, these electrodes function as “wet-dry” interfaces rather than purely dry electrodes.

    The long-term hydration and stable electrochemical performance arise from the deep eutectic solvent (Gly:ChCl), which is non-volatile and hygroscopic. Unlike water-based hydrogels or conventional electrolytic gels, the DES does not evaporate under ambient conditions and remains confined within the polymer matrix. When combined with PEDOT:LS and gelatin, the DES forms a mixed ionic–electronic conductive eutectogel that retains ionic mobility and mechanical compliance over extended periods without requiring rehydration (page 5).

13. The lower muscular SNR in EMG recording during hand flexion is ascribable to what?

    The lower EMG signal amplitude and SNR observed during hand flexion are attributable to the anatomical placement of the electrode array. Electrodes were positioned on the right lateral side of the forearm with the palm facing up, placing them closer to muscle fibres responsible for wrist extension (extensor carpi radialis longus and brevis, extensor carpi ulnaris) than to the flexor muscles, which lie deeper on the ventral side of the forearm. This anatomical arrangement naturally leads to stronger EMG signals during

wrist extension and smaller signals during hand flexion. This explanation has been added to the main text, along with a reference to Supplementary Figure S28 showing the array placement (page 8).

14. How many recording/subjects did you use for training the model?

    All models were trained and evaluated using data from a single subject. For the classification experiments, we acquired 100 movement recordings per movement type from this subject, which were used to construct the training and evaluation datasets. This information has now been explicitly clarified in the main text (at different locations) to avoid any ambiguity.

15. A quantification about the conformability and robustness of the electrode platform would be needed to position your device among other SoA devices.

    We have expanded the characterisation of the electrode platform in the revised manuscript and Supplementary Information Figures S5-S10. Mechanical robustness is now evaluated through controlled bending, minimum bending radius and cyclic strain tests, as well as simulated motion artefact measurements, demonstrating stable impedance and signal quality under repeated deformation. We also quantify inter-electrode variability within arrays and reproducibility across independently fabricated batches, showing consistent electrical performance.

Reviewer #4 (Remarks to the Author):

1. While initial testing is provided, it remains unclear whether the system offers novel features or advantages over existing multichannel EEG/EMG setups.

   We have clarified in the revised manuscript that the novelty of our platform lies in the integration of spatially dense, gel-free, multilayer textile arrays capable of simultaneous multimodal EEG and EMG recording in a single wearable system. Unlike conventional multichannel setups, which often require separate wired electrodes, gels, or rigid connectors, our approach enables concurrent acquisition with independent digitisation paths, eliminating cross-talk while maintaining high spatial resolution (page 3).

   The fabrication workflow, combining blade-coated silver ink, PDMS lamination, laser-patterned masking, silver epoxy interconnects, and PEDOT:LS-based eutectogel deposition, is scalable and compatible with industrial printing or additive manufacturing, producing reproducible, mechanically robust, multilayer arrays. Mechanical and electrical characterisation, including minimum bending radius, cyclic loading, and simulated motion artefacts, demonstrates stable connectivity and signal quality comparable to commercial Ag/AgCl electrodes, while avoiding gels and reducing wiring complexity (Supplementary Figures S5-S10).

   To contextualise these advantages relative to existing technologies, Supplementary Table S1 now provides a comprehensive comparison with representative textile- and flexible-electrode systems, highlighting improvements in multimodality, spatial density, mechanical robustness, and wearable integration. Together, these elements substantiate

the practical and conceptual advance of our approach over standard multichannel EEG/EMG methods.

2. The authors recorded brain responses to visual, motor, and auditory stimuli, which inherently have well-defined temporal structures. However, no evoked responses are shown, and I strongly recommend including them.

   We have now included stimulus-locked evoked responses for the visual, auditory, and motor paradigms in the Supplementary Information (Supplementary Fig. S32). ERPs were computed by averaging EEG segments time-locked to the expected stimulus onset based on the experimental timing structure (10 s baseline followed by repeated 2 s stimulation and 2 s rest periods) and across anatomically grouped electrode regions to improve interpretability. The resulting plots show low-amplitude, consistent responses across cortical regions, providing a qualitative visualisation of stimulus-related temporal dynamics. Because explicit event markers were not recorded and stimulus timing was reconstructed from the paradigm, these evoked responses should be interpreted as illustrative confirmation of time-locked activity rather than precise, latency-resolved ERP components.

3. Since EEG spectra have both periodic and aperiodic components—where the aperiodic part often corresponds to noise—it would be beneficial to present spectra with noise estimates.

   We appreciate the reviewer's suggestion regarding separating periodic and aperiodic components of the EEG spectra. In Supplementary Figure S33, we provide PSDs computed from all channels and averaged across trials for visual, auditory, and motor tasks, with decomposition using FOOOF into the aperiodic 1/f background and periodic peaks. For visual and auditory stimuli, the spectra are very similar, showing a broad spectral peak spanning approximately 6–30 Hz, with modest periodic peaks around 9 Hz, 20 Hz, and 30 Hz, corresponding roughly to alpha and beta frequency ranges. The aperiodic 1/f background is shown as a dashed line. In the case of motor activity, these peaks are more pronounced, and an additional high-frequency power elevation at ~80 Hz is clearly visible, reflecting increased high-frequency activity during motor execution. This noise-aware spectral representation confirms that structured oscillatory components exist above the aperiodic background and complements the feature extraction used in the machine learning analyses.

4. The relationship between the provided confusion matrices and induced versus evoked EEG activity is unclear; clarification and separate classification results for both types would strengthen the study.

   The confusion matrices presented in the main text correspond to classification using evoked, stimulus-locked EEG features. To further investigate the contribution of different types of EEG activity, we additionally performed classification using induced (non-phase-locked) EEG features. The classifier achieved near-perfect classification accuracy (100% overall) for evoked responses, whereas performance based on induced activity remained poor across all three stimuli, between 5-10%. This outcome is consistent with the

expectation that evoked features carry temporally aligned, stimulus-specific information, while induced activity is asynchronous and does not provide discriminative power across trials. These additional analyses have been included in the Supplementary Information as Figure S34, showing separate confusion matrices for evoked and induced EEG.

5. For demonstrating EEG modulation by movement, I suggest including ERD/ERS analyses of alpha and beta oscillations in time domain, as such data are highly informative for neuroscientists and physiologists.

   We have now included ERD/ERS analyses of alpha (8–12 Hz) and beta (13–30 Hz) oscillations for motor-related EEG activity in the Supplementary Information (Figure S35). The analyses reveal a temporally structured pattern: a negative peak ~2 s before movement (potentially reflecting anticipatory cortical activity), a positive peak at movement onset corresponding to synchronisation during motor execution, and a subsequent negative peak ~2–3 s post-onset returning to baseline. Shading shows trial-to-trial variability.

6. Notably, despite discussing cortico-muscular coherence (CMC) in the introduction, the authors do not compute it directly, instead analysing power spectra. Since CMC, especially from raw EEG and EMG signals, could highlight the benefits of the new electrode arrays by revealing distinct activation patterns, including it would significantly enhance the manuscript. This is particularly important given that similar recordings exist (Vidaurre et al., 2019), so the authors should clarify the novelty of their approach. Lastly, presenting direct comparisons showing that their system outperforms current multielectrode methods would provide persuasive evidence of its value.

   Vidaurre, C., Nolte, G., de Vries, I. E. J., Gómez, M., Boonstra, T. W., Müller, K. R., Villringer, A., & Nikulin, V. V. (2019). Canonical maximization of coherence: A novel tool for investigation of neuronal interactions between two datasets. Neuroimage, 201, 116009. doi:10.1016/j.neuroimage.2019.116009

   We thank the reviewer for highlighting the importance of cortico-muscular coherence (CMC) analysis. In response, we have computed the event-based CMC for hand flexion from raw EEG and EMG recordings. The resulting figure in the supporting information (Figure S36) shows the mean coherence spectrum with the beta band (15–30 Hz) highlighted. For clarity, we present only the mean CMC in the figure, which sufficiently demonstrates the beta-band cortico-muscular coupling.

   This analysis confirms that our new electrode arrays capture physiologically meaningful CMC patterns, comparable in magnitude and spectral location to prior reports (e.g., Vidaurre et al., 2019), while offering higher spatial resolution across the forearm muscles. This complements the power spectral analyses already included in the supporting information and supports the novelty of our approach.

While a formal head-to-head comparison with conventional multielectrode arrays is beyond the scope of this manuscript, our recordings demonstrate that the new array resolves distinct EMG activation patterns across multiple forearm muscles simultaneously, which is difficult to achieve with standard arrays. This enhanced spatial coverage and signal specificity supports the potential of our system to reveal cortico-muscular interactions more comprehensively.

RESPONSE TO REVIEWER COMMENTS

The authors thank the reviewer for their constructive comments and for the careful evaluation of the revised manuscript.

Reviewer #4 (Remarks to the Author):

1. Thank you for addressing many of my concerns. However, I am surprised that the authors did not record triggers for the evoked responses. Since evoked responses represent one of the most important measures obtained with EEG, it is very important to show typical waveforms for auditory and visual responses. Bar plots do not provide sufficient information and I am surprised for instance that visual responses have the strongest amplitude over the central areas while they should be largest over parieto-occipital areas (Figure S32). I strongly encourage authors to provide traditional waveforms and topographies of the evoked responses.

   We apologise for the confusion in our previous response, which may have implied that no event triggers were recorded at all. Triggers were recorded, but not within the same software used for EEG acquisition. For visual and auditory stimuli, the start time of the programmed stimulus sequence was recorded, while for motor stimuli, the timing was derived from simultaneously recorded muscle responses.

   We agree that bar plots do not adequately convey the temporal dynamics of evoked responses. Accordingly, we have removed the bar plots in the previous Supplementary Figure S32 and now present standard ERP waveforms for all three stimulus types, alongside topographical maps at early, mid, and late post-stimulus windows. The previously observed dominant amplitude in central electrodes for visual stimuli resulted from an isolated voltage peak in a single central electrode. To improve robustness, we have normalised each electrode to its baseline SD units, reducing the influence of outliers and providing a more accurate view of spatial patterns.

2. Figure S33. Is it only one subject? It would be nice to see classic alpha oscillations in the recordings. At the moment I do not understand whether it is just one specific example or whether electrodes can't record traditional pronounced alpha oscillations.

   The original figure presented channel-averaged spectra from a single subject, which may have obscured spatially localised oscillatory activity. To address this, we have extended Supplementary Figure S33 to show region-specific power spectral densities across anterior frontal, frontal, central, temporal, parietal, and occipital electrode groups, along with a zoomed view of the alpha band (7.5–12.5 Hz). This revised analysis shows alpha-band activity (8–12 Hz) with spatial heterogeneity across conditions. In particular, alpha-band power shows relatively posterior weighting during visual stimulation, a more distributed spatial pattern during auditory stimulation, and comparatively weaker and more distributed expression during motor stimulation, with a slight anterior weighting in some regions. These results indicate that the absence of a clear alpha peak in the original figure was due to spatial averaging rather than a lack of measurable alpha-band activity in the recordings.
